# Transcriptome analysis reveals mucin 4 to be highly associated with periodontitis and identifies pleckstrin as a link to systemic diseases

**DOI:** 10.1038/srep18475

**Published:** 2015-12-21

**Authors:** Anna Lundmark, Haleh Davanian, Tove Båge, Gunnar Johannsen, Catalin Koro, Joakim Lundeberg, Tülay Yucel-Lindberg

**Affiliations:** 1 Department of Dental Medicine, Division of Periodontology, Karolinska Institutet, SE-141 04 Huddinge, Sweden; 2 KTH Royal Institute of Technology, Science for Life Laboratory, School of Biotechnology, Division of Gene Technology, SE-171 65 Solna, Sweden

## Abstract

The multifactorial chronic inflammatory disease periodontitis, which is characterized by destruction of tooth-supporting tissues, has also been implicated as a risk factor for various systemic diseases. Although periodontitis has been studied extensively, neither disease-specific biomarkers nor therapeutic targets have been identified, nor its link with systemic diseases. Here, we analyzed the global transcriptome of periodontitis and compared its gene expression profile with those of other inflammatory conditions, including cardiovascular disease (CVD), rheumatoid arthritis (RA), and ulcerative colitis (UC). Gingival biopsies from 62 patients with periodontitis and 62 healthy subjects were subjected to RNA sequencing. The up-regulated genes in periodontitis were related to inflammation, wounding and defense response, and apoptosis, whereas down-regulated genes were related to extracellular matrix organization and structural support. The most highly up-regulated gene was mucin 4 (*MUC4*), and its protein product was confirmed to be over-expressed in periodontitis. When comparing the expression profile of periodontitis with other inflammatory diseases, several gene ontology categories, including inflammatory response, cell death, cell motion, and homeostatic processes, were identified as common to all diseases. Only one gene, pleckstrin (*PLEK*), was significantly overexpressed in periodontitis, CVD, RA, and UC, implicating this gene as an important networking link between these chronic inflammatory diseases.

Periodontitis, a common chronic inflammatory disease characterized by tissue and bone destruction that ultimately leads to tooth loss, is initiated when oral pathogens gain access to the gingival tissue and stimulate a host immune and inflammatory response. Approximately 49% of the population suffers from generalized periodontitis^1^,^2^. Individual susceptibility to this disease involves interactions between multiple genes and environmental factors, such as smoking and stress. Periodontitis shares many characteristics with other chronic inflammatory diseases and has been associated with several systemic inflammatory disorders, including cardiovascular disease (CVD), the inflammatory bowel disease ulcerative colitis (UC), and Rheumatoid Arthritis (RA)^3^,^4^,^5^.

The hallmarks of periodontitis are host inflammatory events, including the release and activation of inflammatory mediators and cytokines such as interleukins (ILs) and tumor necrosis factor α (TNF-α), as well as proteolytic enzymes such as matrix metalloproteinases (MMPs). The chronic inflammatory process in the gingival tissue is also characterized by infiltration of inflammatory cells such as cytotoxic T lymphocytes, B lymphocytes, and macrophages^6^,^7^. In patients with periodontitis, the levels of the inflammatory mediators IL-1β, TNF-α, and prostaglandin E_2_ are elevated in the gingival crevicular fluid (GCF), which reflects serum and local tissue conditions^8^,^9^,^10^. In addition, the proteolytic MMP enzymes – especially MMP2, MMP8, and MMP9 – have been detected in gingival tissues, saliva, and GCF, and associated with the progression of periodontal diseases^11^,^12^,^13^,^14^. However, to date no diagnostic and prognostic biomarkers or therapeutic targets for periodontitis have been identified.

Among the few studies on the gingival tissue transcriptome of patients with periodontitis^15^, Demmer *et al.*^16^ found that the processes that were expressed differentially in inflamed and non-inflamed tissue were associated with apoptosis, the humoral antimicrobial response, antigen presentation, regulation of metabolic processes, signal transduction, and angiogenesis. Another study, which included only a limited number of patients, showed that immune processes were more highly up-regulated in patients with periodontitis in comparison to healthy controls^17^. These investigations involved microarrays, which are characterized by lower sensitivity, a more limited dynamic range, and higher technical variation than RNA sequencing (RNA-Seq)^18^,^19^,^20^. We are the only group so far to have applied RNA-seq to examine the transcriptome associated with periodontitis, using inflamed and non-inflamed tissue from patients with periodontitis, and showed that the genes up-regulated in inflamed tissue were mainly related to immune and inflammatory responses^21^.

Now, for the first time, we have applied massively parallel RNA-seq to a large number of biopsies from patients with periodontitis and from healthy subjects in order to characterize the global transcriptome of periodontitis, as well as its association to other chronic inflammatory disorders. The analysis identified 1268 genes differentially expressed between patients with periodontitis and healthy controls. The up-regulated genes in periodontitis were primarily related to inflammation, response to wounding, and apoptosis, whereas genes associated with extracellular matrix organization and structural support were down-regulated. The most highly up-regulated gene was *MUC4* and the second was *MMP7*. Further characterization of the association between periodontitis and the chronic inflammatory disorders CVD, RA, and UC identified only one gene, pleckstrin (*PLEK*), that was commonly up-regulated in all four diseases, suggesting this gene as a key link between periodontitis and various systemic diseases associated with periodontitis.

## Results

### Subject characteristics

The characteristics of the 124 participants, 62 patients with periodontitis and 62 healthy subjects without periodontitis, are shown in Table 1. As expected, the patients with periodontitis exhibited a greater probing depth (mean 8 mm versus ≤3 mm) and more bleeding upon probing (79% versus 21%) than healthy subjects.

### Higher degree of inflammation in gingival tissue samples from patients with periodontitis

**Figure 1:**
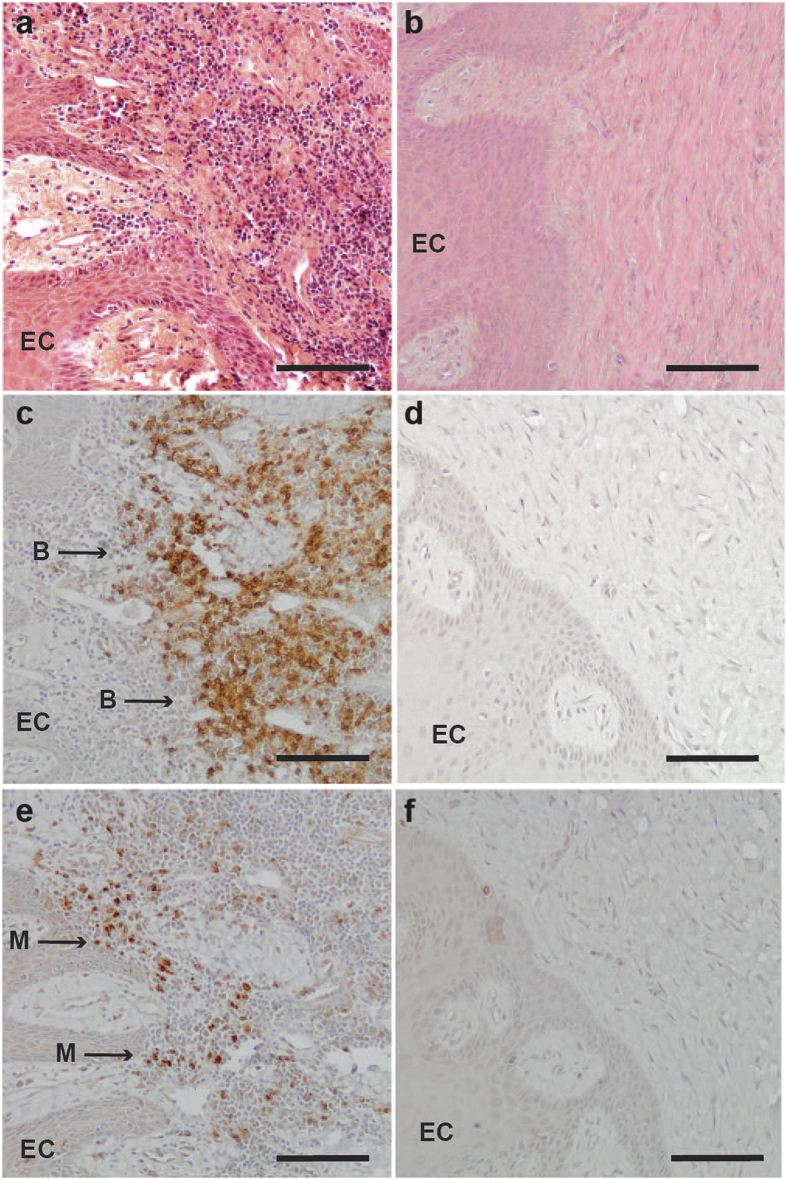
Histological and immunohistological staining of gingival biopsies from patients with periodontitis and healthy control subjects. (**a**) Representative Hematoxylin-Eosin (H&E) staining of gingival tissue from a patient with periodontitis and (**b**) a healthy control subject. Representative immunohistological staining with (**c,d**) CD20, a B plasma cell marker, and (**e,f**) CD68, a macrophage marker, of gingival tissue from a patient with periodontitis and a healthy control subject. EC = epithelial cells, B = B cells, M = macrophages. Scale bars = 100 μm.

To assess gingival inflammation, the biopsies from all 124 subjects were stained both with H&E (Fig. 1a,b) and cell-type-specific antibodies (Fig. 1c–f). Evaluation of inflammatory cell infiltration into gingival biopsies by three “blinded” observers, revealed significantly more inflammation (*P* < 0.01) in the group with periodontitis (Table 1). Immunohistochemical staining revealed that 85% of the biopsies from periodontitis patients expressed the B-cell marker CD20, as opposed to only 40% of those from controls (Fig. 1c,d; Supplementary Table S1). This represents a significantly higher (*P* < 0.01) expression in patients with periodontitis than healthy controls. The corresponding values for the macrophage marker CD68 were 85% and 45% (Fig. 1e,f; Supplementary Table S1; *P* = 0.02).

### Identification of significant patterns of gene expression using principal component analysis (PCA) and orthogonal projections to latent structures discriminant analysis (OPLS-DA)

**Figure 2:**
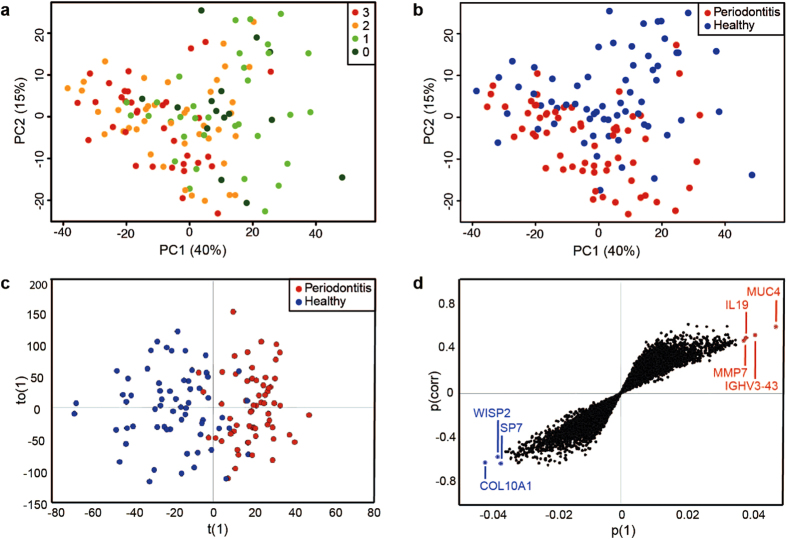
Multivariate analysis of gene expression data from gingival biopsies. (**a**) Principal component analysis (PCA) score plot with the two first principal components (PC1 and PC2) plotted on the x- and y-axis. Each data point representing one sample is color-coded according to the extent of inflammatory cell infiltration (evaluated in biopsies adjacent to those used for gene expression analysis and harvested at the same time). Dark green = 0 (no evidence of inflammatory infiltration), light green = 1 (slight inflammatory infiltration), orange = 2 (moderate inflammatory infiltration) and red = 3 (severe inflammatory infiltration). (**b**) The PCA score plot with data points from patients with periodontitis and healthy control subjects represented in red and blue, respectively. (**c**) Orthogonal projections to latent structures discriminant analysis (OPLS-DA) score plot for gingival biopsies from patients with periodontitis (red) and healthy control subjects (blue). The between-group variation predictive component, t(1), is plotted against the within-group variation orthogonal component, to (1). (**d**) S-plot of the OPLS-DA data showing the magnitude of each gene’s contribution to the separation, p(1), in relationship to its significance, p(corr). The genes contributing the most to the periodontitis group (highlighted in red) are, from left to right, *MMP7*, *IL19*, *IGHV3-43*, and *MUC4*. The genes contributing the most to the healthy control group (highlighted in blue) are *COL10A1*, *WISP2*, and *SP7*.

Multivariate analyses were performed to summarize and obtain an overview of the gene expression data. To identify causes of variation and potential outliers or biases within the dataset, an unsupervised PCA model was created. This model revealed that the largest variation, known as the first principal component (PC1), explained 40% of the variance within the dataset, while the second principal component (PC2) explained 15%. Under this model, the samples aligned along the PC1 according to their degree of inflammation (Fig. 2a), whereas the PC2 tended to separate the periodontitis and healthy control samples from each other (Fig. 2b).

Furthermore, the supervised OPLS-DA approach was applied to the dataset to identify genes whose expression in the two groups differed most. A model with one predictive and two orthogonal components successfully distinguished between the periodontitis and healthy samples along the first predictive component (Fig. 2c). The degree of variation explained by this model (R^2^) was 0.629 and the value describing the model’s predictive ability (Q^2^) was 0.304. In addition, to identify potential biomarkers, an S-plot was created on the basis of the OPLS-DA (Fig. 2d). The magnitude of the contribution of each gene to the separation in the OPLS-DA model (p[1]) was plotted against the significance of this same gene (p[corr]). The genes contributing most to the periodontitis group, with highest significance, were *MUC4*, *MMP7*, interleukin 19 (*IL19*), and immunoglobulin heavy variable 3–43 (*IGHV3-43*), while the corresponding genes in the healthy subjects were collagen type X alpha 1 (*COL10A1*), WNT1-inducible signaling pathway protein 2 (*WISP2*), and Sp7 transcription factor (*SP7*) (Fig. 2d).

### Identification of up- and down-regulated genes and gene ontology (GO) categories in periodontitis

**Figure 3:**
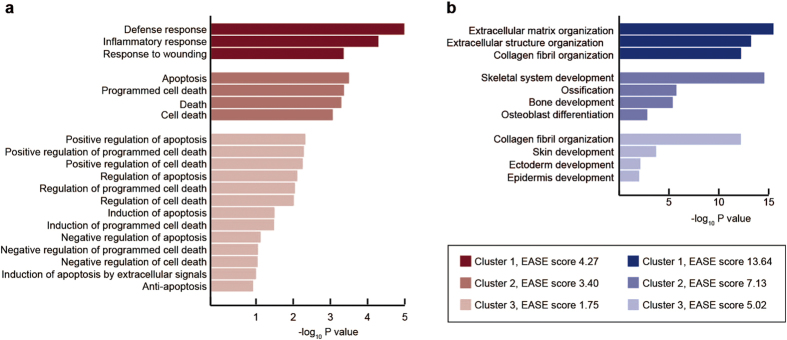
Gene ontology (GO) categories enriched among the genes up- and down-regulated in association with periodontitis. Biological processes that are up- or down-regulated in connection with periodontitis were identified using the annotation tool DAVID and related processes clustered using the functional annotation clustering tool. (**a**) The up-regulated processes with the highest enrichment (EASE) scores included items related to defense, inflammation and wounding responses as well as apoptosis and cell death. (**b**) The down-regulated gene clusters with the highest enrichment scores were related to organization of the extracellular matrix and structure, organization of collagen fibrils, as well as development of the skeleton, skin, ectoderm and epidermis.

Of the 22,810 protein-coding genes identified by the RNA-seq analysis, 665 were up-regulated and 633 down-regulated in periodontitis in comparison to healthy tissue (with the threshold q-value, an adjusted *P*-value, of 0.01). The functional annotation clustering tool in the Database for Annotation, Visualization and Integrated Discovery (DAVID), which clusters related groups and orders these clusters according to their significance (as determined by their EASE scores, a modified Fisher exact *P*-value), revealed that the three clusters of up-regulated genes with the highest significance included items related to defense, inflammation, and wounding responses as well as apoptosis and cell death (Fig. 3a). On the other hand, the biological processes associated with down-regulated genes were related to organization of the extracellular matrix and structure, organization of collagen fibrils as well as development of the skeleton, skin, ectoderm, and epidermis (Fig. 3b).

The 10 most potently up- and down-regulated genes in gingival tissue from patients with periodontitis are demonstrated in Table 2 together with the Ensembl ID, the gene symbol, a description of the gene product, the fold change, and the log_2_ fold change. The complete lists of up- and down-regulated genes can be found as Supplementary Table S2a,b online. *MUC4* was the most highly up-regulated gene, with a fold change of 7.1, while *MMP7* was the second most, with a fold change of 6.8 (Table 2). The overexpression of these genes was also identified by the OPLS-DA as highly significant. The two most down-regulated genes in periodontitis were keratin 71 (*KRT71*) and *SP7* (fold change <−10 in both cases; Table 2), of which the latter was also identified by the OPLS-DA.

### Upregulation of *MUC4* and *MMP7* at the mRNA and protein levels in periodontitis

**Figure 4:**
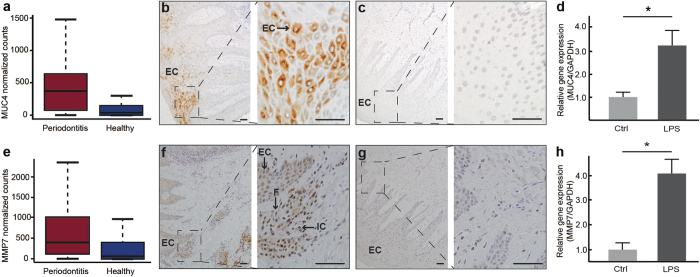
MUC4 and MMP7 mRNA and protein expression in gingival biopsies and gingival cells. (**a**) Boxplot demonstrating the normalized number of transcripts of *MUC4* in gingival tissue from patients with periodontitis and healthy controls. (**b**) Representative staining for MUC4 in the gingival epithelium from a patient with periodontitis and (**c**) a healthy control subject. EC = epithelial cells. Scale bars = 50 μm. (**d**) Relative gene expression of *MUC4* in gingival epithelial cells treated with lipopolysaccharide (LPS), for 24 hours, compared to control cells (Ctrl) treated with medium only, expressed as fold change normalized to the expression of *GAPDH*. Data are shown as mean ± S.D. from triplicates (*P < 0.001) (**e**) Normalized number of transcripts of *MMP7* in gingival tissue from patients with periodontitis and healthy subjects. (**f**) Representative immunostaining for MMP7 in gingival tissue from a patient with periodontitis and (**g**) a healthy subject. EC = epithelial cells, IC = immune cells, F = fibroblast. Scale bars = 50 μm. (**h**) Relative gene expression of *MMP7* in gingival fibroblast cells stimulated with lipopolysaccharide (LPS) (24 hours) compared to control cells treated with medium only (Ctrl), expressed as fold change normalized to the expression of *GAPDH*. Results shown are representative of three experiments performed in gingival fibroblasts obtained from three individuals and data presented as mean ± S.D. from triplicates (*P < 0.001).

The two most up-regulated genes in connection with periodontitis, *MUC4* and *MMP7*, were investigated further. The normalized counts for these genes in patients with periodontitis and healthy controls are demonstrated in Fig. 4a,e. To evaluate the expression of the corresponding proteins, immunohistochemical staining of gingival biopsies from 20 patients and 20 controls was performed. The MUC4 protein was detected in the gingival epithelial cells of 18 (90%) patients with periodontitis but only 2 (10%) of the healthy control subjects (*P* < 0.001; Fig. 4b,c; Supplementary Table S3). Evaluation of the MMP7 protein expression, as assessed by scoring the epithelium and connective tissue of all gingival biopsies, showed a significantly (*P* < 0.01) higher expression of MMP7 in the connective tissue of the periodontitis group, with a mean value of 2.1, compared to that of the non-periodontitis group, with a mean value of 1.0. In contrast, there was no significant difference in protein expression of MMP7 in the epithelium (*P* = 0.40; Fig. 4f,g; Supplementary Table S3).

### Comparative gene expression analysis of periodontitis, CVD, RA, and UC

**Figure 5:**
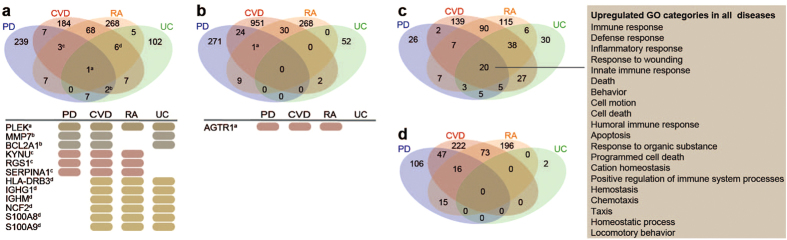
Comparative gene expression and GO analysis for periodontitis, CVD, RA, and UC. Venn diagrams representing the numbers of common and specific (**a**) up-regulated and (**b**) down-regulated genes in periodontitis (PD), cardiovascular disease (CVD), Rheumatoid Arthritis (RA), and ulcerative colitis (UC), together with the gene names for the genes common to all or three diseases. (**c**) Venn diagram demonstrating the numbers of common and specific up-regulated gene ontology (GO) categories including the list of 20 up-regulated GO categories common in PD, CVD, RA, and UC. (**d**) Venn diagram showing the numbers of common and specific down-regulated GO categories in all diseases.

To identify common key genes, we compared the differentially expressed genes acquired through our RNA-seq analysis with differentially expressed genes obtained from the chronic inflammatory diseases CVD, RA, and UC (diseases implicated to be associated with periodontitis). Only one gene, *PLEK*, was identified as commonly up-regulated in all four diseases (Fig. 5a), with fold changes of 1.6 in periodontitis, 1.5 in CVD, 4.1 in RA, and 1.8 in UC. Additionally, *MMP7* and B-cell lymphoma 2-related protein A1 (*BCL2A1)* were up-regulated across periodontitis, CVD, and UC, while the genes kynureninase (*KYNU*), regulator of G-protein signaling 1 (*RGS1*) and serpin peptidase inhibitor, clade A (*SERPINA1*) were up-regulated across periodontitis, CVD, and RA (Fig. 5a). With regard to down-regulated genes, there was no gene common to all four diseases, although one gene, angiotensin II type I receptor (*AGTR1*), was down-regulated in periodontitis, CVD, and RA (Fig. 5b). To further investigate whether the differentially expressed genes in these diseases encode similar processes, GO analysis was performed on all up- and down-regulated genes for all four diseases. There were 20 up-regulated GO categories common to all diseases, including inflammatory response, cell death, cell motion, and homeostatic processes (Fig. 5c). However, there was no down-regulated process common to all four diseases (Fig. 5d).

### Expression of PLEK in gingival tissue

**Figure 6:**
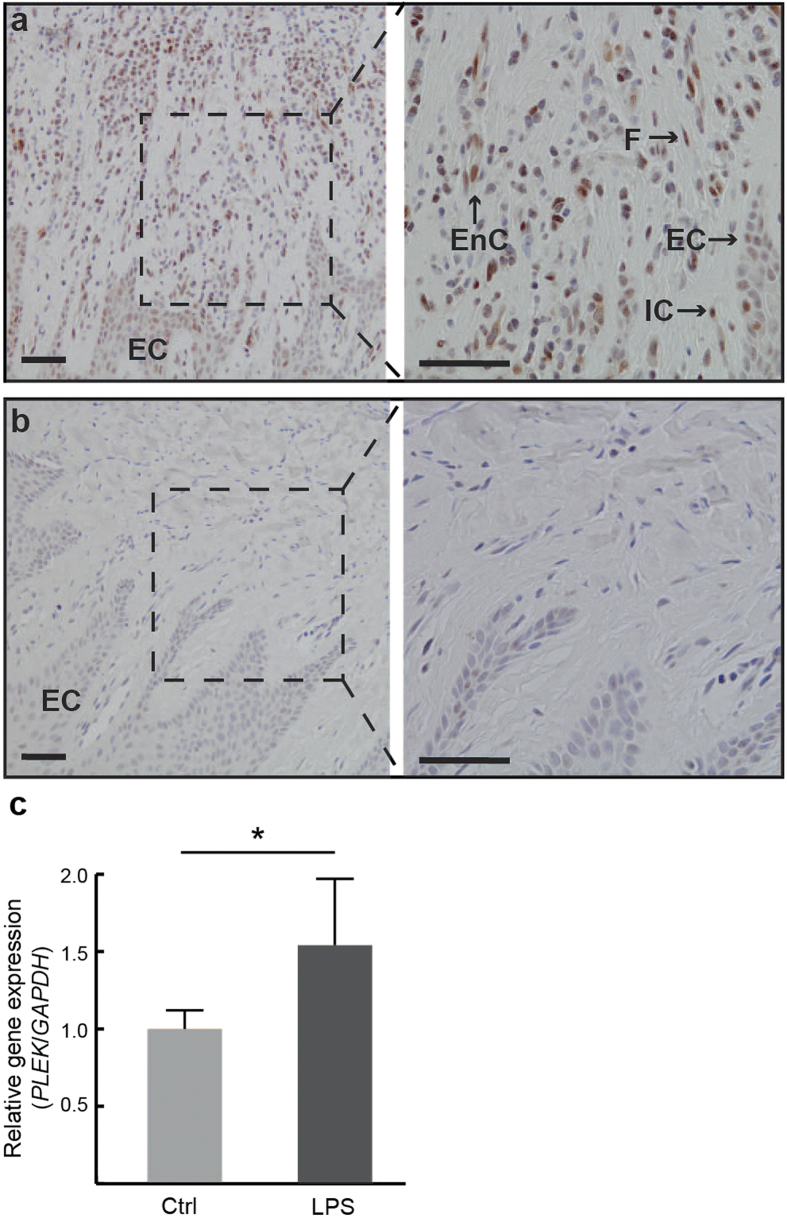
Expression of PLEK in gingival biopsies and gingival fibroblasts. (**a**) Representative PLEK staining of gingival tissue sections from a patient with periodontitis and (**b**) a healthy control subject. EC = epithelial cells, F = fibroblasts, EnC = endothelial cells, IC = immune cells. Scale bars = 50 μm. (**c**) Relative gene expression of *PLEK* in fibroblast cells stimulated with lipopolysaccharide (LPS) compared to control cells treated with medium only (Ctrl), expressed as fold change normalized to the expression of *GAPDH*. Results shown are representative of three experiments performed in gingival fibroblasts obtained from three individuals and data presented as mean ± S.D. from triplicates (*P < 0.01).

Based on our findings identifying *PLEK* as a commonly up-regulated gene in all four diseases, we further investigated the protein expression of this gene in gingival tissue biopsies from patients with periodontitis and healthy controls. Immunohistochemical staining of gingival tissue samples of patients with periodontitis with PLEK showed positively stained fibroblast-like cells, epithelial cells, immune cells, and endothelial cells (Fig. 6a). In contrast, gingival tissue from healthy controls exhibited low proportion of positively stained cells for PLEK (Fig. 6b).

### Regulation of *MUC4*, *MMP7*, and *PLEK* in human gingival fibroblasts and gingival epithelial cells

In the next series of experiments, the regulation of *MUC4, MMP7*, and *PLEK* was investigated *in vitro* using gingival fibroblasts (the predominant cells of gingival connective tissue) and gingival epithelial cells. The regulation of *MUC4*, which was exclusively expressed in the epithelium of gingival tissue of patients with periodontitis, was studied in gingival epithelial cells. Treatment of these cells with lipopolysaccharide (LPS) for 24 hours significantly (*P* < 0.001) increased the mRNA expression of *MUC4* (Fig. 4d). Similarly, LPS treatment of gingival fibroblasts for 24 hours significantly increased the mRNA expression of *MMP7* (*P* < 0.001) and *PLEK* (*P* < 0.01), shown in Figs 4h and 6c, respectively. The mRNA expression of *MMP7* and *PLEK*, in contrast to *MUC4*, was not affected by LPS treatment in epithelial cells (data not shown).

## Discussion

The present RNA-seq study of the global transcriptome of periodontitis identified *MUC4* and *MMP7* as the two most highly up-regulated genes in periodontitis. We also report, for the first time, that *PLEK* is commonly up-regulated in periodontitis and the chronic inflammatory diseases CVD, RA, and UC.

We confirmed that inflammatory cell infiltration into gingival tissue was more extensive in the periodontitis group than in healthy controls. In addition, our PCA model, based on gene expression data, showed the largest variation within all samples to be associated with the degree of inflammation. However, the PCA model also revealed specific patterns distinguishing periodontitis from healthy tissue irrespective of the degree of inflammation, suggesting that other processes may be involved. This suggestion is further supported by our GO category analysis identifying, in addition to up-regulation of immune responses, up-regulation of apoptosis and down-regulation of extracellular matrix organization and structural support. The up- and down-regulation of these processes may contribute to the disruption of tissue homeostasis that characterizes the pathogenesis of periodontitis.

The most highly up-regulated gene associated with periodontitis was *MUC4*, a completely novel finding. Mucins, glycoproteins provided by mucus-producing epithelial cells, form a physical barrier, have anti-microbial properties, and contribute to the clearance of microbes. The production of mucins is stimulated by neutrophils, microbial products, and different inflammatory mediators, including TNF-α and IL-1β. This interaction forms a link between mucins, innate mucosal immunity, and mucosal inflammatory responses^22^. In addition, MUC4 has also been implicated in cancer by mediating the epidermal growth factor family of enzymes, including the tyrosine kinase receptor ERBB2. This results in activation of downstream signal transduction pathways, such as protein kinase C (PKC)^23^,^24^, known to be involved in inflammation^25^. The overexpression of MUC4 in the epithelial gingival tissue of patients with periodontitis suggests that this protein might also be present at elevated levels in saliva and GCF, providing non-invasive diagnostic markers for early detection and monitoring of the progression of periodontitis. Thus, studies are ongoing to investigate the levels of MUC4 in saliva and GCF in a large cohort of patients with periodontitis and healthy controls.

The MMP family of enzymes degrades the extracellular matrix and components of the basement membrane, and the enzymes MMP2, 8, and 9 have previously been shown to be associated with periodontitis^26^,^27^,^28^. In the current study, *MMP7* was identified as the second most highly up-regulated gene in periodontitis. MMP7 is an epithelial matrix metalloproteinase that degrades fibronectin, laminin, type IV collagen, gelatin, elastin, and proteoglycans^29^,^30^. Its overall role in periodontal disease, however, has not previously been characterized. Our study is the first to link MMP7 to periodontitis, showing it to be overexpressed at the protein level in gingival connective tissue, but not in the gingival epithelium of patients with periodontitis. This latter observation supports the proposal that MMP7 is constitutively expressed in epithelia to provide a defense against microorganisms^31^. MMP7 has been proposed to underlie pulmonary injury in mice, possibly by helping to recruit neutrophils whose oxidative burst can destroy connective tissue, while *MMP7*-deficient mice are protected, and we propose a similar defense mechanism in periodontitis^31^,^32^. Thus, our current findings indicate that MMP7 may play a crucial role in the pathogenesis of periodontitis.

To explore the transcriptomic association between periodontitis and systemic diseases, we further compared our gene expression data with equivalent data obtained from patients with various chronic inflammatory conditions, including CVD, RA, and UC. Our analysis revealed that similar processes related to immune responses, cell motion, cell death, and homeostasis were up-regulated in all diseases. However, although several GO categories were commonly up-regulated in all four diseases, only a few genes were commonly up-regulated, suggesting that different genes contribute to similar processes in the various diseases.

Interestingly, when comparing differentially expressed genes from all four diseases, only one gene, *PLEK*, was up-regulated in all inflammatory diseases investigated in the current study. One reason for our identification of a single gene common to all diseases might be the comparison of our RNA-seq data with data sets obtained using microarray technology. Several studies have shown that RNA-seq outperforms microarray technology^33^,^34^,^35^,^36^, mainly due to higher precision in detecting transcript levels because of lower background noise and overcoming problems with probe hybridization bias, which allows for better quantification of differentially expressed genes^37^.

PLEK is a PKC substrate inducibly expressed by macrophages; it is suggested to be an important intermediate in the secretion and activation pathways of the pro-inflammatory cytokines TNF-α and IL-1β^38^,^39^. In addition, it has been shown that the expression of PLEK is induced in macrophages in response to the bacterial stimuli LPS^40^. This is in agreement with our current results showing an up-regulation of *PLEK* in gingival fibroblasts stimulated with LPS. Up-regulation of PLEK by oral bacterial products^22^ in combination with activation of PKC pathways in response to overexpression of MUC4 may contribute to the initiation and maintenance of chronic inflammation. Moreover, *AGTR1* – the only down-regulated gene in CVD, RA and periodontitis – may also be involved in the disruption of tissue homeostasis since it has been reported to stimulate cell proliferation by activation of PKC enzymes^41^. In light of our novel finding that *PLEK* is up-regulated in the conditions investigated here, we hypothesize that this protein may act as an important link associating periodontitis with other chronic inflammatory diseases, possibly through activation via periodontal bacteria translocated into the circulatory system. Thus, the functional relevance of this gene, as well as its contribution to various chronic systemic diseases, needs to be further elucidated.

The genes identified as up-regulated in three out of four diseases – *MMP7*, *BCL2A1*, *KYNU*, *RGS1*, and *SERPINA1* – are all involved in inflammatory responses and are induced by a wide range of external stimuli, including LPS and TNF-α^42^,^43^,^44^,^45^,^46^,^47^. Furthermore, RGS1 and SERPINA1 activate the mitogen-activated protein kinase (MAPK) signaling pathway implicated in the control of inflammatory responses^45^,^48^,^49^, while MMP7 have been reported to be induced by the MAPK signaling pathway^50^. The enzyme KYNU is involved in the kynurenine pathway, which plays a key role in the increased prevalence of cardiovascular disease by regulating inflammation, immune activation and oxidative stress^51^, also reported to activate the MAPK pathway^52^. BCL2A1, reported to act anti-apoptotically, is induced by the transcription factor NF-κB, which controls numerous genes involved in inflammatory diseases, such as atherosclerosis, bowel disease and arthritis^44^,^53^. Thus, the related functions and shared signaling pathways of these commonly up-regulated genes identified here imply that they may act together in maintaining chronic inflammation.

In summary, by applying RNA-seq to a relatively large number of gingival biopsies, both from patients with periodontitis and healthy control subjects, we identified numerous up- and down-regulated genes. The top two most up-regulated genes were *MUC4* and *MMP7*, and the corresponding proteins of these genes were also overexpressed in gingival tissue from patients with periodontitis. Our novel findings suggest MUC4 and MMP7 to be potential biomarkers and therapeutic targets for periodontitis. Finally, we also report that periodontitis shares commonly up-regulated processes with other chronic inflammatory diseases and, for the first time, we identify *PLEK* to be up-regulated in several inflammatory conditions associated with periodontitis, which highlights this gene as an important link between periodontitis and other chronic inflammatory diseases.

## Materials and Methods

### Ethics Statement

This study was performed in accordance with the Declaration of Helsinki and obtained ethical board approval. The clinical dental examination, collection of human gingival tissue samples and experimental procedures were approved by the Regional Ethics Board in Stockholm (number 2008/1935-31/3), and written informed consent was obtained from all participants.

### Subjects and collection of gingival biopsies

A total of 144 participants, 71 patients with periodontitis and 73 healthy controls, were initially included in the study. The patients with periodontitis had tooth sites with probing depth ≥6 mm, clinical attachment level ≥5 mm, and bleeding on probing. Healthy control subjects showed no signs of periodontal disease, with no gingival/periodontal inflammation, a probing depth ≤3.0 mm, a clinical attachment level ≤3.5 mm, and no bleeding on probing. None of the participants used nicotine or nicotine-replacement medications. The healthy controls were all undergoing implant surgery for tooth loss due to other reasons (e.g., caries and accidents), which allowed biopsies to be harvested at the same time. The biopsies were collected from an area distal or mesial to the tooth and close to an edentulous area. Such an area may be harder to clean and thus may have a higher degree of local inflammation. However, none of the healthy controls showed any signs of periodontitis.

Two gingival biopsies of similar size were harvested from the same site in each subject. One was stored in RNA Later (Applied Biosystems, Massachusetts, USA) overnight at 4 °C and thereafter at −80 °C for subsequent RNA isolation, and the second biopsy was fixed in 4% neutral buffered formalin and embedded in paraffin for histological and immunohistochemical analysis.

### Histological and immunohistochemical staining

Serial sections (4 μm thick) of the fixed and embedded biopsies were deparaffinized and stained with Hematoxylin-Eosin (H&E). The degree of inflammatory cell infiltration into the gingival tissues was evaluated by three “blinded” observers, where 0 = no evidence of inflammatory infiltration, 1 = slight inflammatory infiltration, 2 = moderate inflammatory infiltration, and 3 = severe inflammatory infiltration. For each sample, the median value from the three observers was calculated. Then the mean of these values was obtained for each group, and the difference between the groups was assessed using the Mann-Whitney U test. The difference between groups was considered significant at *P* < 0.05. The intraclass correlation coefficient, representing the consistency of the rating of the observers, was 0.64.

The sections were pre-heated at 60 °C for 60 minutes, followed by a deparaffinization procedure. After applying the Dako Target Retrieval solution for CD20, CD68, and MMP7 (Dako Sweden AB) and 10 mM sodium citrate-0.05% Tween 20 (pH 6.0) for MUC4 and PLEK, the sections were blocked in 1% H_2_O_2_ in phosphate-buffered saline (PBS) with Saponin for 60 minutes at room temperature (RT) and then incubated with 3% bovine serum albumin for 30 minutes at RT. Mouse monoclonal antibodies, directed against CD20 (diluted 1:100; Leica Biosystems, Stockholm, Sweden), CD68 (1:50, Dako Sweden AB), PLEK (1:50, Abcam, Cambridge, England), and MUC4 and MMP7 (1:100, Abcam, Cambridge, England) were used.

After incubation overnight at 4 °C with these primary antibodies, sections were blocked with 1% normal goat serum for 15 minutes and thereafter incubated with a horseradish-peroxidase-conjugated secondary polyclonal anti-mouse antibody (Dako Sweden AB, Stockholm, Sweden) for 40 minutes at RT in the dark. Afterwards, the Avidin-Biotin Complex solution (Vector laboratories, Burlingame, CA, USA) was applied for 40 minutes at RT in the dark. The sections were washed with PBS and peroxidase activity visualized. Finally, the slides were dehydrated, mounted, and photographed under a light microscope. In parallel with each immunohistochemical staining, IgG was used as a negative control following the same protocol and all the sections were counterstained with hematoxylin.

The extent of MMP7-positive cells in the epithelium and connective tissue was evaluated by three “blinded” observers using a relative scale from 0 to 3 and the results were analyzed as described above. The intraclass correlation coefficient was 0.94 and 0.91 for the scoring of the epithelium and connective tissue, respectively. The sections stained for MUC4, CD20, or CD68, were simply examined for the presence of positively stained cells. The results for the periodontitis and healthy control groups were compared using Fisher’s exact test and the differences were considered significant at *P* < 0.05.

### RNA extraction

RNA was extracted from the 144 biopsies using the Qiagen RNeasy kit (VWR, Stockholm, Sweden), and its quality was assessed using the RNA 6000 NanoLabChip kit of the Bioanalyzer system from Agilent Technologies (Santa Clara, CA, USA). Samples with an RNA Integrity Number (RIN) <7 and a yield <0.4 μg were excluded from further analysis, leaving 65 samples from patients with periodontitis and 64 from healthy controls with mean RIN values of 8.8 and 8.9, respectively.

### Preparation and sequencing of RNA libraries

RNA libraries were prepared using the TruSeq Stranded mRNA sample prep kit with 96 dual indexes (Illumina, CA, USA) in accordance with the manufacturer’s instructions except for the following changes: The protocols were carried out automatically on an Agilent NGS workstation (Agilent, CA, USA) using the purification steps described by Lundin *et al.*^54^ and Borgström *et al.*^55^. After clustering on cBot, the samples were sequenced on a HiSeq 2500 as recommended by the manufacturer. Demultiplexing and conversion were performed with CASAVA v1.8.2. software (Illumina, CA, USA) employing the Sanger/phred33/Illumina 1.8+ quality scale. On average, each run generated 44.3 million paired-end reads per sample, of which 81% mapped to the human genome. For 5 samples, the sequencing quality was low and these were excluded from further analysis, leaving 62 samples from patients and 62 from healthy controls.

### Sequence alignment and analysis

The paired-end sequences were aligned to the human genome assembly, build GRCh37/hg19, with STAR software version 2.3.1o using standard parameters^56^. The numbers of aligned reads per gene were determined with HTSeq version 0.6.1 (http://www-huber.embl.de/users/anders/HTSeq/doc/overview.html), with standard parameters, except for “–s reverse.” Mapping statistics were calculated from numbers obtained by running “bam_stat.py” (included in rseqc version 2.3.6) on bamfiles.

### Principal component analysis (PCA) and orthogonal projections to latent structures discriminant analysis (OPLS-DA)

The read counts were used to perform PCA (in R version 3.1.0 using the prcomp function) and the OPLS-DA (Simca 13.0.3, Umetrics, Umeå, Sweden). After adjusting the raw counts by a trimmed mean of M values (TMM) scaling in the Bioconductor/edgeR package^57^, followed by a transformation into log_2_ counts per million, the 500 genes with the highest variability were used in the PCA model. When the degree of inflammation was analyzed with this model, one patient was excluded due to the lack of a second biopsy. The data were log-transformed and subjected to unit variance scaling prior to OPLS-DA and estimation of the model complexity by cross-validation. In the case of the S-plot, pareto-scaling was used instead of unit variance scaling.

### Differential gene expression analysis

The R/Bioconductor package samr version 2.0^58^ was used to normalize the data and assess differential expression on the basis of the read counts using a False Discovery Rate (FDR) <0.01 as the threshold. The q-value, obtained by assessing differential expression with SAMSeq in the samr package, describes the *P*-values as corrected for the errors arising from multiple testing in an FDR approach^59^. Through the Ensembl ID’s of the genes found to be differentially expressed, gene names and descriptions were retrieved using the R/Bioconductor package biomaRt^60^ or, in a few cases, from www.ensembl.org.

### Gene Ontology (GO) category analysis

The default parameters of the Database for Annotation, Visualization and Integrated Discovery (DAVID)^61^ were used to achieve functional annotation clustering of the genes that were up-regulated and down-regulated. *Homo sapiens* was used as background and the “GOTERM_BP_FAT” option selected.

### Association analysis of periodontitis, cardiovascular disease (CVD), Rheumatoid Arthritis (RA), and ulcerative colitis (UC)

To obtain differentially expressed genes for diseases previously implicated to be associated with periodontitis, a literature search for transcriptome analyses of these inflammatory conditions was conducted. Studies on tissue biopsies from patients with disease and healthy controls, that reported up- and down-regulated genes, or raw data, were included in the analysis. To obtain corresponding differentially expressed genes in CVD, GSE21545^62^, and GSE13760^63^, datasets containing carotid plaque samples and healthy arterial tissue were downloaded from the National Center for Biotechnology Information’s (NCBI) Gene Expression Omnibus repository (GEO) and analyzed as previously described by Sikorski *et al.*^64^. Genes differentially expressed between synovial tissue from patients with RA and healthy controls were downloaded from Ungethuem *et al.*^65^, and genes differentially expressed between colon biopsies from patients with UC and healthy controls were downloaded from Noble *et al.*^66^. All obtained microarray data were normalized in R version 3.1.0 using Robust Multi-array Average (RMA) and differentially expressed genes were assessed using samr^58^ with an FDR threshold of 0.01. A cutoff fold change of 1.5 was applied to all gene lists. Enrichment analyses of GO categories were performed using DAVID, as described above, except for using the Functional Annotation Chart tool instead of the Functional Annotation Clustering tool. All statistical analyses were performed in R version 3.1.0.

### Culturing and stimulation of human gingival fibroblasts and epithelial cells

Gingival fibroblasts, established from gingival biopsies obtained from three individuals with no clinical signs of periodontitis, were cultured as described previously^67^. Human primary gingival epithelial cells were purchased from CellnTec (CellnTec Advanced Cell Systems AG, Bern, Switzerland) and cultured in CnT-PR cell culture medium (CellnTec) at 37 °C in a 5% CO_2_ incubator. The cells (0.5 × 10^6^) were seeded in Petri dishes (60 mm) in DMEM supplemented with penicillin (50 units/ml), streptomycin (50 μg/ml), and 5% fetal calf serum (FCS, Technologies Europe BV) in case of the fibroblasts, and in iCnT-PR cell culture medium (CellnTec) in case of the epithelial cells, and cultured at 37 °C for 24 hours. The cell layers were rinsed twice with serum-free medium (DMEM or CnT-PR medium) followed by the addition of 2.0 ml of medium in the absence or presence of 5 μg/ml Porphyromonas gingivalis or E.coli LPS (InvivoGen, Toulouse, France). After incubation for 24 hours, the culture medium was collected and the cells washed twice with ice-cold PBS and used for total isolation of RNA.

### Quantitative PCR

Total RNA was extracted from LPS-stimulated gingival fibroblasts/epithelial cells and unstimulated control cells using the RNeasy Mini Kit (Qiagen, Valencia, CA, USA) and quantified at 260/280 nm, with a Nanodrop 2000 spectrophotometer (Thermo Scientific, MA, USA). cDNA synthesis was performed from 1.0 μg RNA using the iScript™ cDNA Synthesis Kit (BioRad, Herkules, CA, USA) according to the manufacturer’s instructions. Analysis of *PLEK, MMP7, MUC4*, and *GAPDH* was performed by quantitative PCR (qPCR) using cDNA from the fibroblast cells and TaqMan Universal PCR Master Mix together with the TaqMan probes Hs001160164_m1 for *PLEK*, Hs01042796_m1 for *MMP7*, Hs02569088_s1 for *MUC4* and Hs02758991_g1 for *GAPDH* (Applied Biosystems, Waltham, MA, USA). Analysis of *MUC4* and *GAPDH* was performed on cDNA isolated from epithelial cells. All samples were run in triplicates. The PCR conditions were 50 °C for 2 minutes, followed by 95 °C for 10 minutes, and then 40 cycles of 95 °C for 15 seconds and 60 °C for 1 minute. Relative gene expression was calculated using the ΔΔCt method^68^ where the mean Ct for *PLEK*, *MMP7*, and *MUC4* were normalized against the mean Ct for *GAPDH* for each sample. All the experiments were performed in cells established from at least three individuals and reproducible data representing one of the experiments is demonstrated. The significance of the difference between groups was assessed by the Student’s *t*-test (two-tailed) with the difference considered significant at *P* < 0.05.

## Additional Information

**How to cite this article**: Lundmark, A. *et al.* Transcriptome analysis reveals mucin 4 to be highly associated with periodontitis and identifies pleckstrin as a link to systemic diseases. *Sci. Rep.* **5**, 18475; doi: 10.1038/srep18475 (2015).

## Supplementary Material

Supplementary Tables

## Figures and Tables

**Table 1:** Characteristics of the study participants and the degree of inflammation in gingival biopsies from patients with periodontitis and healthy controls without periodontal disease.

	Periodontitis (n = 62)	Controls (n = 62)
Gender (female/male)	33/29	37/25
Mean age (±SD, years)	64 ± 11	56 ± 16
Mean probing depth (±SD, mm)	8.2 ± 1.7	≤3
Bleeding on probing (n)	49/62	13/62
Degree of inflammation (0–3)^a^ in gingival samples including the number of subjects (n)^b^ and mean value.	0 (n = 4)	0 (n = 11)
	1 (n = 17)	1 (n = 21)
	2 (n = 19)	2 (n = 17)
	3 (n = 22)	3 (n = 12)
	Mean 2.0^c^	Mean 1.5^c^

^a^0 = no evidence of inflammatory infiltration, 1 = slight inflammatory infiltration, 2 = moderate inflammatory infiltration and 3 = severe inflammatory infiltration.

^b^The degree of inflammation from one patient is excluded due to the lack of a second biopsy.

^c^*P* < 0.01 for the difference between periodontitis and healthy controls as assessed by Mann-Whitney U test.

**Table 2:** The 10 genes up- and down-regulated to the greatest extent in association with periodontitis.

Ensembl ID	Gene symbol	Gene product	Fold change	log_2_ fold change
Up-regulated				
ENSG00000145113	*MUC4*	mucin 4, cell surface associated	7.1	2.8
ENSG00000137673	*MMP7*	matrix metallopeptidase 7 (matrilysin, uterine)	6.8	2.8
ENSG00000211658	*IGLV3-27*	immunoglobulin lambda variable 3–27	6.4	2.7
ENSG00000242515	*UGT1A10*	UDP glucuronosyltransferase 1 family, polypeptide A10	6.0	2.6
ENSG00000240671	*IGKV1-8*	immunoglobulin kappa variable 1–8	5.8	2.5
ENSG00000232216	*IGHV3-43*	immunoglobulin heavy variable 3–43	5.7	2.5
ENSG00000142224	*IL19*	interleukin 19	5.1	2.4
ENSG00000140835	*CHST4*	carbohydrate (N-acetylglucosamine 6-O) sulfotransferase 4	4.5	2.2
ENSG00000211892	*IGHG4*	immunoglobulin heavy constant gamma 4 (G4m marker)	4.4	2.2
ENSG00000211611	*IGKV6-21*	immunoglobulin kappa variable 6–21 (non-functional)	4.2	2.1
Down-regulated				
ENSG00000139648	*KRT71*	keratin 71	<−10	>10
ENSG00000170374	*SP7*	Sp7 transcription factor	<−10	>10
ENSG00000131668	*BARX1*	BARX homeobox 1	−4.5	2.2
ENSG00000105664	*COMP*	cartilage oligomeric matrix protein	−4.3	2.1
ENSG00000104435	*STMN2*	stathmin-like 2	−3.6	1.9
ENSG00000269729	*AC006262.4*	lincRNA	−3.6	1.8
ENSG00000231605	*LINC01363*	long intergenic non-protein coding RNA 1363	−3.5	1.8
ENSG00000178172	*SPINK6*	serine peptidase inhibitor, Kazal type 6	−3.5	1.8
ENSG00000146250	*PRSS35*	protease, serine, 35	−3.4	1.8
ENSG00000130600	*H19*	H19, imprinted maternally expressed transcript (non-protein coding)	−3.3	1.7
ENSG00000211890	*COL1A1*	collagen, type I, alpha 1	−3.1	1.7

Genes with a q-value (*P*-value adjusted for multiple testing^59^) <0.01 were considered to be significantly differentially expressed.

## References

[b1] Kholy K. E., Genco R. J. & Van Dyke T. E. Oral infections and cardiovascular disease. Trends Endocrinol. Metab. 26, 315–321 (2015). 25892452 10.1016/j.tem.2015.03.001

[b2] Eke P. I. *et al.* Prevalence of periodontitis in adults in the United States: 2009 and 2010. J. Dent. Res. 91, 914–920 (2012). 22935673 10.1177/0022034512457373

[b3] Hajishengallis G. Periodontitis: from microbial immune subversion to systemic inflammation. Nat. Rev. Immunol. 15, 30–44 (2014). 25534621 10.1038/nri3785 PMC4276050

[b4] Pihlstrom B. L., Michalowicz B. S. & Johnson N. W. Periodontal diseases. Lancet 366, 1809–1820 (2005). 16298220 10.1016/S0140-6736(05)67728-8

[b5] Sanz M., van Winkelhoff A. J. & Working Group 1 of Seventh European Workshop on, P. Periodontal infections: understanding the complexity–consensus of the Seventh European Workshop on Periodontology. J. Clin. Periodontol. 38 Suppl 11, 3–6 (2011). 21323698 10.1111/j.1600-051X.2010.01681.x

[b6] Darveau R. P. Periodontitis: a polymicrobial disruption of host homeostasis. Nat. Rev. Microbiol. 8, 481–490 (2010). 20514045 10.1038/nrmicro2337

[b7] Yucel-Lindberg T. & Bage T. Inflammatory mediators in the pathogenesis of periodontitis. Expert Rev. Mol. Med. 15, e7 (2013). 23915822 10.1017/erm.2013.8

[b8] Trindade F. *et al.* Uncovering the molecular networks in periodontitis. Proteomics Clin. Appl. 8, 748–761 (2014). 24828325 10.1002/prca.201400028 PMC4426160

[b9] Gorska R. *et al.* Relationship between clinical parameters and cytokine profiles in inflamed gingival tissue and serum samples from patients with chronic periodontitis. J. Clin. Periodontol. 30, 1046–1052 (2003). 15002890 10.1046/j.0303-6979.2003.00425.x

[b10] Kamma J., Mombelli A., Tsinidou K., Vasdekis V. & Giannopoulou C. Cytokines in gingival crevicular fluid of adolescents and young adults. Oral Microbiol. Immunol. 24, 7–10 (2009). 19121063 10.1111/j.1399-302X.2008.00466.x

[b11] Maeso G., Bravo M. & Bascones A. Levels of metalloproteinase-2 and -9 and tissue inhibitor of matrix metalloproteinase-1 in gingival crevicular fluid of patients with periodontitis, gingivitis, and healthy gingiva. Quintessence Int. 38, 247–252 (2007). 17334003

[b12] Rai B., Kharb S., Jain R. & Anand S. C. Biomarkers of periodontitis in oral fluids. J. Oral Sci. 50, 53–56 (2008). 18403884 10.2334/josnusd.50.53

[b13] Romanelli R. *et al.* Activation of neutrophil collagenase in periodontitis. Infect. Immun. 67, 2319–2326 (1999). 10225890 10.1128/iai.67.5.2319-2326.1999 PMC115973

[b14] Sapna G., Gokul S. & Bagri-Manjrekar K. Matrix metalloproteinases and periodontal diseases. Oral Dis. 20, 538–550 (2014). 23849049 10.1111/odi.12159

[b15] Grant M. M. What do ‘omic technologies have to offer periodontal clinical practice in the future? J. Periodontal Res. 47, 2–14 (2012). 21679186 10.1111/j.1600-0765.2011.01387.x

[b16] Demmer R. T. *et al.* Transcriptomes in healthy and diseased gingival tissues. J. Periodontol. 79, 2112–2124 (2008). 18980520 10.1902/jop.2008.080139 PMC2637651

[b17] Becker S. T. *et al.* Peri-implantitis versus periodontitis: functional differences indicated by transcriptome profiling. Clin. Implant Dent. Relat. Res. 16, 401–411 (2014). 22967131 10.1111/cid.12001

[b18] Marioni J. C., Mason C. E., Mane S. M., Stephens M. & Gilad Y. RNA-seq: an assessment of technical reproducibility and comparison with gene expression arrays. Genome Res. 18, 1509–1517 (2008). 18550803 10.1101/gr.079558.108 PMC2527709

[b19] Wang Z., Gerstein M. & Snyder M. RNA-Seq: a revolutionary tool for transcriptomics. Nat. Rev. Genet. 10, 57–63 (2009). 19015660 10.1038/nrg2484 PMC2949280

[b20] Westermann A. J., Gorski S. A. & Vogel J. Dual RNA-seq of pathogen and host. Nat. Rev. Microbiol. 10, 618–630 (2012). 22890146 10.1038/nrmicro2852

[b21] Davanian H. *et al.* Gene expression profiles in paired gingival biopsies from periodontitis-affected and healthy tissues revealed by massively parallel sequencing. PLoS One 7, e46440 (2012). 23029519 10.1371/journal.pone.0046440 PMC3460903

[b22] Linden S. K., Sutton P., Karlsson N. G., Korolik V. & McGuckin M. A. Mucins in the mucosal barrier to infection. Mucosal Immunol. 1, 183–197 (2008). 19079178 10.1038/mi.2008.5 PMC7100821

[b23] Kufe D. W. Mucins in cancer: function, prognosis and therapy. Nat. Rev. Cancer 9, 874–885 (2009). 19935676 10.1038/nrc2761 PMC2951677

[b24] Mukhopadhyay P. *et al.* MUC4 overexpression augments cell migration and metastasis through EGFR family proteins in triple negative breast cancer cells. PLoS One 8, e54455 (2013). 23408941 10.1371/journal.pone.0054455 PMC3569463

[b25] Sobhia M. E. *et al.* Protein kinase C inhibitors: a patent review (2008–2009). Expert Opin. Ther. Pat. 23, 1297–1315 (2013). 10.1517/13543776.2013.805205 23795914

[b26] Uitto V. J., Overall C. M. & McCulloch C. Proteolytic host cell enzymes in gingival crevice fluid. Periodontol. 2000 31, 77–104 (2003). 12656997 10.1034/j.1600-0757.2003.03106.x

[b27] Makela M. *et al.* Matrix metalloproteinase 2 (gelatinase A) is related to migration of keratinocytes. Exp. Cell Res. 251, 67–78 (1999). 10438572 10.1006/excr.1999.4564

[b28] Sorsa T. *et al.* Matrix metalloproteinases: contribution to pathogenesis, diagnosis and treatment of periodontal inflammation. Ann. Med. 38, 306–321 (2006). 16938801 10.1080/07853890600800103

[b29] Saarialho-Kere U. K. Patterns of matrix metalloproteinase and TIMP expression in chronic ulcers. Arch. Dermatol. Res. 290 Suppl, S47–54 (1998). 9710383 10.1007/pl00007453

[b30] Wilson C. L. & Matrisian L. M. Matrilysin: an epithelial matrix metalloproteinase with potentially novel functions. Int. J. Biochem. Cell Biol. 28, 123–136 (1996). 8729000 10.1016/1357-2725(95)00121-2

[b31] Parks W. C., Wilson C. L. & Lopez-Boado Y. S. Matrix metalloproteinases as modulators of inflammation and innate immunity. Nat. Rev. Immunol. 4, 617–629 (2004). 15286728 10.1038/nri1418

[b32] Li Q., Park P. W., Wilson C. L. & Parks W. C. Matrilysin shedding of syndecan-1 regulates chemokine mobilization and transepithelial efflux of neutrophils in acute lung injury. Cell 111, 635–646 (2002). 12464176 10.1016/s0092-8674(02)01079-6

[b33] Fu X. *et al.* Estimating accuracy of RNA-Seq and microarrays with proteomics. BMC Genomics 10, 161 (2009). 19371429 10.1186/1471-2164-10-161 PMC2676304

[b34] Sirbu A., Kerr G., Crane M. & Ruskin H. J. RNA-Seq vs dual- and single-channel microarray data: sensitivity analysis for differential expression and clustering. PLoS One 7, e50986 (2012). 23251411 10.1371/journal.pone.0050986 PMC3518479

[b35] Wang C. *et al.* The concordance between RNA-seq and microarray data depends on chemical treatment and transcript abundance. Nat. Biotechnol. 32, 926–932 (2014). 25150839 10.1038/nbt.3001 PMC4243706

[b36] Xu X. *et al.* Parallel comparison of Illumina RNA-Seq and Affymetrix microarray platforms on transcriptomic profiles generated from 5-aza-deoxy-cytidine treated HT-29 colon cancer cells and simulated datasets. BMC Bioinformatics 14 Suppl 9, S1 (2013). 23902433 10.1186/1471-2105-14-S9-S1 PMC3697991

[b37] Zhao S., Fung-Leung W. P., Bittner A., Ngo K. & Liu X. Comparison of RNA-Seq and microarray in transcriptome profiling of activated T cells. PLoS One 9, e78644 (2014). 24454679 10.1371/journal.pone.0078644 PMC3894192

[b38] Ding Y. *et al.* Phosphorylation of pleckstrin increases proinflammatory cytokine secretion by mononuclear phagocytes in diabetes mellitus. J. Immunol. 179, 647–654 (2007). 17579087 10.4049/jimmunol.179.1.647 PMC2150995

[b39] Hasturk H., Kantarci A. & Van Dyke T. E. Oral inflammatory diseases and systemic inflammation: role of the macrophage. Front. Immunol. 3, 118 (2012). 22623923 10.3389/fimmu.2012.00118 PMC3353263

[b40] Brumell J. H. *et al.* Expression of the protein kinase C substrate pleckstrin in macrophages: association with phagosomal membranes. J. Immunol. 163, 3388–3395 (1999). 10477609

[b41] Greco S. *et al.* Activation of angiotensin II type I receptor promotes protein kinase C translocation and cell proliferation in human cultured breast epithelial cells. J. Endocrinol. 174, 205–214 (2002). 12176659 10.1677/joe.0.1740205

[b42] de Serres F. & Blanco I. Role of alpha-1 antitrypsin in human health and disease. J. Intern. Med. 276, 311–335 (2014). 24661570 10.1111/joim.12239

[b43] Gu J. *et al.* Identification of RGS1 as a candidate biomarker for undifferentiated spondylarthritis by genome-wide expression profiling and real-time polymerase chain reaction. Arthritis Rheum. 60, 3269–3279 (2009). 19877080 10.1002/art.24968 PMC2936922

[b44] Ottina E., Tischner D., Herold M. J. & Villunger A. A1/Bfl-1 in leukocyte development and cell death. Exp. Cell Res. 318, 1291–1303 (2012). 22342458 10.1016/j.yexcr.2012.01.021 PMC3405526

[b45] Pak H. K. *et al.* Regulator of G protein signaling 1 suppresses CXCL12-mediated migration and AKT activation in RPMI 8226 human plasmacytoma cells and plasmablasts. PLoS One 10, e0124793 (2015). 25897806 10.1371/journal.pone.0124793 PMC4405207

[b46] Vecsei L., Szalardy L., Fulop F. & Toldi J. Kynurenines in the CNS: recent advances and new questions. Nat. Rev. Drug Discov. 12, 64–82 (2013). 23237916 10.1038/nrd3793

[b47] Hayden D. M., Forsyth C. & Keshavarzian A. The role of matrix metalloproteinases in intestinal epithelial wound healing during normal and inflammatory states. J. Surg. Res. 168, 315–324 (2011). 20655064 10.1016/j.jss.2010.03.002

[b48] Dabbagh K. *et al.* Alpha-1-antitrypsin stimulates fibroblast proliferation and procollagen production and activates classical MAP kinase signalling pathways. J. Cell. Physiol. 186, 73–81 (2001). 11147816 10.1002/1097-4652(200101)186:1<73::AID-JCP1002>3.0.CO;2-Q

[b49] Arthur J. S. & Ley S. C. Mitogen-activated protein kinases in innate immunity. Nat. Rev. Immunol. 13, 679–692 (2013). 23954936 10.1038/nri3495

[b50] Jia Z. C. *et al.* Tissue factor/activated factor VIIa induces matrix metalloproteinase-7 expression through activation of c-Fos via ERK1/2 and p38 MAPK signaling pathways in human colon cancer cell. Int. J. Colorectal Dis. 27, 437–445 (2012). 22076613 10.1007/s00384-011-1351-0

[b51] Wang Q., Liu D., Song P. & Zou M. H. Tryptophan-kynurenine pathway is dysregulated in inflammation, and immune activation. Front Biosci (Landmark Ed) 20, 1116–1143 (2015). 25961549 10.2741/4363 PMC4911177

[b52] Runchel C., Matsuzawa A. & Ichijo H. Mitogen-activated protein kinases in mammalian oxidative stress responses. Antioxid. Redox Signal. 15, 205–218 (2011). 21050144 10.1089/ars.2010.3733

[b53] Monaco C. *et al.* Canonical pathway of nuclear factor kappa B activation selectively regulates proinflammatory and prothrombotic responses in human atherosclerosis. Proc. Natl. Acad. Sci. USA 101, 5634–5639 (2004). 15064395 10.1073/pnas.0401060101 PMC397455

[b54] Lundin S., Stranneheim H., Pettersson E., Klevebring D. & Lundeberg J. Increased throughput by parallelization of library preparation for massive sequencing. PLoS One 5, e10029 (2010). 20386591 10.1371/journal.pone.0010029 PMC2850305

[b55] Borgstrom E., Lundin S. & Lundeberg J. Large scale library generation for high throughput sequencing. PLoS One 6, e19119 (2011). 21589638 10.1371/journal.pone.0019119 PMC3083417

[b56] Dobin A. *et al.* STAR: ultrafast universal RNA-seq aligner. Bioinformatics 29, 15–21 (2013). 23104886 10.1093/bioinformatics/bts635 PMC3530905

[b57] Robinson M. D., McCarthy D. J. & Smyth G. K. edgeR: a Bioconductor package for differential expression analysis of digital gene expression data. Bioinformatics 26, 139–140 (2010). 19910308 10.1093/bioinformatics/btp616 PMC2796818

[b58] Li J. & Tibshirani R. Finding consistent patterns: a nonparametric approach for identifying differential expression in RNA-Seq data. Stat. Methods Med. Res. 22, 519–536 (2013). 22127579 10.1177/0962280211428386 PMC4605138

[b59] Storey J. D. A direct approach to false discovery rates. Journal of the Royal Statistical Society Series B-Statistical Methodology 64, 479–498 (2002).

[b60] Durinck S. *et al.* BioMart and Bioconductor: a powerful link between biological databases and microarray data analysis. Bioinformatics 21, 3439–3440 (2005). 16082012 10.1093/bioinformatics/bti525

[b61] Huang da W., Sherman B. T. & Lempicki R. A. Systematic and integrative analysis of large gene lists using DAVID bioinformatics resources. Nat. Protoc. 4, 44–57 (2009). 19131956 10.1038/nprot.2008.211

[b62] Folkersen L. *et al.* Prediction of ischemic events on the basis of transcriptomic and genomic profiling in patients undergoing carotid endarterectomy. Mol. Med. 18, 669–675 (2012). 22371308 10.2119/molmed.2011.00479 PMC3388132

[b63] Cangemi C. *et al.* Fibulin-1 is a marker for arterial extracellular matrix alterations in type 2 diabetes. Clin. Chem. 57, 1556–1565 (2011). 21926180 10.1373/clinchem.2011.162966

[b64] Sikorski K., Wesoly J. & Bluyssen H. A. Data mining of atherosclerotic plaque transcriptomes predicts STAT1-dependent inflammatory signal integration in vascular disease. Int. J. Mol. Sci. 15, 14313–14331 (2014). 25196434 10.3390/ijms150814313 PMC4159852

[b65] Ungethuem U. *et al.* Molecular signatures and new candidates to target the pathogenesis of rheumatoid arthritis. Physiol. Genomics 42A, 267–282 (2010). 20858714 10.1152/physiolgenomics.00004.2010

[b66] Noble C. L. *et al.* Regional variation in gene expression in the healthy colon is dysregulated in ulcerative colitis. Gut 57, 1398–1405 (2008). 18523026 10.1136/gut.2008.148395

[b67] Yucel-Lindberg T., Olsson T. & Kawakami T. Signal pathways involved in the regulation of prostaglandin E synthase-1 in human gingival fibroblasts. Cell. Signal. 18, 2131–2142 (2006). 16766159 10.1016/j.cellsig.2006.04.003

[b68] Livak K. J. & Schmittgen T. D. Analysis of relative gene expression data using real-time quantitative PCR and the 2(-Delta Delta C(T)) Method. Methods 25, 402–408 (2001). 11846609 10.1006/meth.2001.1262

