# Supplementary material for: Transcriptome analysis reveals mucin 4 to be highly associated with periodontitis and identifies pleckstrin as a link to systemic diseases
Source: Sci Rep. 2015 Dec 21;5:18475. doi: 10.1038/srep18475 (PMC4685297; doi:10.1038/srep18475)
Supplement: Supplementary Tables [file srep18475-s1.pdf]

Transcriptome analysis reveals *Mucin 4* to be highly associated with periodontitis and identifies *Pleckstrin* as a link to systemic diseases

Anna Lundmark[1†], Haleh Davanian[1†], Tove Båge[1], Gunnar Johannsen[1], Catalin Koro[1], Joakim Lundeberg[2], Tülay Yucel-Lindberg[1*]

[1]Department of Dental Medicine, Division of Periodontology, Karolinska Institutet, SE-141 04 Huddinge, Sweden, [2]KTH Royal Institute of Technology, Science for Life Laboratory, School of Biotechnology, Division of Gene Technology, SE-171 65 Solna, Sweden

[†]These authors contributed equally to this work

[*]Correspondence and requests for materials should be addressed to T.Y.L (e-mail: tulay.lindberg@ki.se)

Table S1 Expression of markers for B plasma cells (CD20) and macrophages (CD68) in gingival biopsies from patients with periodontitis and healthy control subjects.

Periodontitis			Control subjects		
**Subject**	**CD20**	**CD68**	**Subject**	**CD20**	**CD68**
**1**	+	+	**21**	-	+
**2**	-	-	**22**	-	-
**3**	+	+	**23**	+	+
**4**	+	+	**24**	+	-
**5**	+	+	**25**	-	-
**6**	-	+	**26**	+	+
**7**	+	+	**27**	-	-
**8**	+	-	**28**	+	+
**9**	+	+	**29**	-	-
**10**	-	-	**30**	+	+
**11**	+	+	**31**	-	-
**12**	+	+	**32**	+	+
**13**	+	+	**33**	-	+
**14**	+	+	**34**	+	-
**15**	+	+	**35**	+	+
**16**	+	+	**36**	-	-
**17**	+	+	**37**	-	-
**18**	+	+	**38**	-	+
**19**	+	+	**39**	-	-
**20**	+	+	**40**	-	-
**Frequency (+/-)**	**17/3[a]**	**17/3[b]**	**Frequency (+/-)**	**8/12[a]**	**9/11[b]**

(+) evidence of CD20 or CD68 expression, (-) no evidence of CD20 or CD68 expression.
[a]$P < 0.01$ for the difference between periodontitis and controls.
[b]$P < 0.05$ for the difference between periodontitis and controls.

Table S2a. Up-regulated genes in periodontitis.

Ensembl ID	Gene symbol	Gene product	Fold change	log_2 fold change
ENSG00000145113	MUC4	mucin 4. cell surface associated [Source:HGNC Symbol;Acc:7514]	7.08	2.82
ENSG00000137673	MMP7	matrix metallopeptidase 7 (matrilysin. uterine) [Source:HGNC Symbol;Acc:7174]	6.83	2.77
ENSG00000211658	IGLV3-27	immunoglobulin lambda variable 3-27 [Source:HGNC Symbol;Acc:5910]	6.38	2.67
ENSG00000242515	UGT1A10	UDP glucuronosyltransferase 1 family. polypeptide A10 [Source:HGNC Symbol;Acc:12531]	6.02	2.59
ENSG00000240671	IGKV1-8	immunoglobulin kappa variable 1-8 [Source:HGNC Symbol;Acc:5743]	5.84	2.54
ENSG00000232216	IGHV3-43	immunoglobulin heavy variable 3-43 [Source:HGNC Symbol;Acc:5604]	5.72	2.52
ENSG00000142224	IL19	interleukin 19 [Source:HGNC Symbol;Acc:5990]	5.08	2.35
ENSG00000140835	CHST4	carbohydrate (N-acetylglucosamine 6-O) sulfotransferase 4 [Source:HGNC Symbol;Acc:1972]	4.54	2.18
ENSG00000211892	IGHG4	immunoglobulin heavy constant gamma 4 (G4m marker) [Source:HGNC Symbol;Acc:5528]	4.44	2.15
ENSG00000211611	IGKV6-21	immunoglobulin kappa variable 6-21 (non-functional) [Source:HGNC Symbol;Acc:5836]	4.2	2.07
ENSG00000211936	IGHV4-4	immunoglobulin heavy variable 4-4 [Source:HGNC Symbol;Acc:5652]	4.18	2.06
ENSG00000254395	IGHV4-55	immunoglobulin heavy variable 4-55 (pseudogene) [Source:HGNC Symbol;Acc:5653]	4.12	2.04
ENSG00000162877	PM20D1	peptidase M20 domain containing 1 [Source:HGNC Symbol;Acc:26518]	4.06	2.02
ENSG00000248590	GLDCP1	glycine dehydrogenase (decarboxylase) pseudogene 1 [Source:HGNC Symbol;Acc:4314]	3.96	1.99
ENSG00000211945	IGHV1-18	immunoglobulin heavy variable 1-18 [Source:HGNC Symbol;Acc:5549]	3.94	1.98
ENSG00000243955	GSTA1	glutathione S-transferase alpha 1 [Source:HGNC Symbol;Acc:4626]	3.91	1.97
ENSG00000108342	CSF3	colony stimulating factor 3 (granulocyte) [Source:HGNC Symbol;Acc:2438]	3.85	1.94
ENSG00000099985	OSM	oncostatin M [Source:HGNC Symbol;Acc:8506]	3.79	1.92
ENSG00000174226	SNX31	sorting nexin 31 [Source:HGNC Symbol;Acc:28605]	3.73	1.9
ENSG00000211890	IGHA2	immunoglobulin heavy constant alpha 2 (A2m marker) [Source:HGNC Symbol;Acc:5479]	3.67	1.88
ENSG00000142623	PADI1	peptidyl arginine deiminase. type I [Source:HGNC Symbol;Acc:18367]	3.65	1.87
ENSG00000134873	CLDN10	claudin 10 [Source:HGNC Symbol;Acc:2033]	3.61	1.85
ENSG00000134339	SAA2	serum amyloid A2 [Source:HGNC Symbol;Acc:10514]	3.44	1.78

ENSG00000178445	GLDC	glycine dehydrogenase (decarboxylating) [Source:HGNC Symbol;Acc:4313]	3.43	1.78
ENSG00000211630	IGKV1D-13	immunoglobulin kappa variable 1D-13 [Source:HGNC Symbol;Acc:5747]	3.4	1.76
ENSG00000211637	IGLV4-69	immunoglobulin lambda variable 4-69 [Source:HGNC Symbol;Acc:5921]	3.3	1.72
ENSG00000211893	IGHG2	immunoglobulin heavy constant gamma 2 (G2m marker) [Source:HGNC Symbol;Acc:5526]	3.27	1.71
ENSG00000247844	CCAT1	colon cancer associated transcript 1 (non-protein coding) [Source:HGNC Symbol;Acc:45128]	3.27	1.71
ENSG00000211967	IGHV3-53	immunoglobulin heavy variable 3-53 [Source:HGNC Symbol;Acc:5610]	3.25	1.7
ENSG00000144908	ALDH1L1	aldehyde dehydrogenase 1 family. member L1 [Source:HGNC Symbol;Acc:3978]	3.22	1.69
ENSG00000182885	GPR97	G protein-coupled receptor 97 [Source:HGNC Symbol;Acc:13728]	3.19	1.67
ENSG00000228314	CYP4F29P	cytochrome P450. family 4. subfamily F. polypeptide 29. pseudogene [Source:HGNC Symbol;Acc:2647]	3.16	1.66
ENSG00000211970	IGHV4-61	immunoglobulin heavy variable 4-61 [Source:HGNC Symbol;Acc:5655]	3.13	1.65
ENSG00000211668	IGLV2-11	immunoglobulin lambda variable 2-11 [Source:HGNC Symbol;Acc:5887]	3.13	1.64
ENSG00000131355	EMR3	egf-like module containing. mucin-like. hormone receptor-like 3 [Source:HGNC Symbol;Acc:23647]	3.12	1.64
ENSG00000118094	TREH	trehalase (brush-border membrane glycoprotein) [Source:HGNC Symbol;Acc:12266]	3.1	1.63
ENSG00000211897	IGHG3	immunoglobulin heavy constant gamma 3 (G3m marker) [Source:HGNC Symbol;Acc:5527]	3.1	1.63
ENSG00000211976	IGHV3-73	immunoglobulin heavy variable 3-73 [Source:HGNC Symbol;Acc:5623]	3.09	1.63
ENSG00000211660	IGLV2-23	immunoglobulin lambda variable 2-23 [Source:HGNC Symbol;Acc:5890]	3.08	1.62
ENSG00000211964	IGHV3-48	immunoglobulin heavy variable 3-48 [Source:HGNC Symbol;Acc:5606]	3.03	1.6
ENSG00000215559	ANKRD20A11P	ankyrin repeat domain 20 family. member A11. pseudogene [Source:HGNC Symbol;Acc:42024]	3.03	1.6
ENSG00000007062	PROM1	prominin 1 [Source:HGNC Symbol;Acc:9454]	3.02	1.59
ENSG00000173391	OLR1	oxidized low density lipoprotein (lectin-like) receptor 1 [Source:HGNC Symbol;Acc:8133]	2.98	1.58
ENSG00000171049	FPR2	formyl peptide receptor 2 [Source:HGNC Symbol;Acc:3827]	2.97	1.57
ENSG00000170477	KRT4	keratin 4 [Source:HGNC Symbol;Acc:6441]	2.97	1.57
ENSG00000211941	IGHV3-11	immunoglobulin heavy variable 3-11 (gene/pseudogene) [Source:HGNC Symbol;Acc:5580]	2.95	1.56
ENSG00000012223	LTF	lactotransferrin [Source:HGNC Symbol;Acc:6720]	2.93	1.55
ENSG00000211655	IGLV1-36	immunoglobulin lambda variable 1-36 [Source:HGNC Symbol;Acc:5876]	2.87	1.52
ENSG00000211671	IGLV2-8	immunoglobulin lambda variable 2-8 [Source:HGNC Symbol;Acc:5895]	2.86	1.52

ENSG00000203688			2.85	1.51
ENSG00000211949	IGHV3-23	immunoglobulin heavy variable 3-23 [Source:HGNC Symbol;Acc:5588]	2.84	1.51
ENSG00000110777	POU2AF1	POU class 2 associating factor 1 [Source:HGNC Symbol;Acc:9211]	2.82	1.5
ENSG00000224373	IGHV4-59	immunoglobulin heavy variable 4-59 [Source:HGNC Symbol;Acc:5654]	2.81	1.49
ENSG00000257335	MGAM	maltase-glucoamylase (alpha-glucosidase) [Source:HGNC Symbol;Acc:7043]	2.77	1.47
ENSG00000243290	IGKV1-12	immunoglobulin kappa variable 1-12 [Source:HGNC Symbol;Acc:5730]	2.74	1.45
ENSG00000211947	IGHV3-21	immunoglobulin heavy variable 3-21 [Source:HGNC Symbol;Acc:5586]	2.73	1.45
ENSG00000103089	FA2H	fatty acid 2-hydroxylase [Source:HGNC Symbol;Acc:21197]	2.71	1.44
ENSG00000272549			2.71	1.44
ENSG00000211632	IGKV3D-11	immunoglobulin kappa variable 3D-11 [Source:HGNC Symbol;Acc:5823]	2.7	1.43
ENSG00000244437	IGKV3-15	immunoglobulin kappa variable 3-15 [Source:HGNC Symbol;Acc:5816]	2.7	1.43
ENSG00000240382	IGKV1-17	immunoglobulin kappa variable 1-17 [Source:HGNC Symbol;Acc:5733]	2.67	1.41
ENSG00000211966	IGHV5-51	immunoglobulin heavy variable 5-51 [Source:HGNC Symbol;Acc:5659]	2.67	1.41
ENSG00000214711	CAPN14	calpain 14 [Source:HGNC Symbol;Acc:16664]	2.63	1.39
ENSG00000211625	IGKV3D-20	immunoglobulin kappa variable 3D-20 [Source:HGNC Symbol;Acc:5825]	2.63	1.39
ENSG00000102243	VGLL1	vestigial like 1 (Drosophila) [Source:HGNC Symbol;Acc:20985]	2.62	1.39
ENSG00000239951	IGKV3-20	immunoglobulin kappa variable 3-20 [Source:HGNC Symbol;Acc:5817]	2.61	1.39
ENSG00000145287	PLAC8	placenta-specific 8 [Source:HGNC Symbol;Acc:19254]	2.6	1.38
ENSG00000140279	DUOX2	dual oxidase 2 [Source:HGNC Symbol;Acc:13273]	2.59	1.37
ENSG00000170956	CEACAM3	carcinoembryonic antigen-related cell adhesion molecule 3 [Source:HGNC Symbol;Acc:1815]	2.58	1.37
ENSG00000223648	IGHV3-64	immunoglobulin heavy variable 3-64 [Source:HGNC Symbol;Acc:5617]	2.58	1.37
ENSG00000163739	CXCL1	chemokine (C-X-C motif) ligand 1 (melanoma growth stimulating activity. alpha) [Source:HGNC Symbol;Acc:4602]	2.57	1.36
ENSG00000243566	UPK3B	uroplakin 3B [Source:HGNC Symbol;Acc:21444]	2.55	1.35
ENSG00000196549	MME	membrane metallo-endopeptidase [Source:HGNC Symbol;Acc:7154]	2.55	1.35
ENSG00000119535	CSF3R	colony stimulating factor 3 receptor (granulocyte) [Source:HGNC Symbol;Acc:2439]	2.55	1.35
ENSG00000137868	STRA6	stimulated by retinoic acid 6 [Source:HGNC Symbol;Acc:30650]	2.54	1.34

ENSG00000140274	DUOXA2	dual oxidase maturation factor 2 [Source:HGNC Symbol;Acc:32698]	2.53	1.34
ENSG00000007171	NOS2	nitric oxide synthase 2. inducible [Source:HGNC Symbol;Acc:7873]	2.5	1.32
ENSG00000185499	MUC1	mucin 1. cell surface associated [Source:HGNC Symbol;Acc:7508]	2.49	1.32
ENSG00000144460	NYAP2	neuronal tyrosine-phosphorylated phosphoinositide-3-kinase adaptor 2 [Source:HGNC Symbol;Acc:29291]	2.48	1.31
ENSG00000010932	FMO1	flavin containing monooxygenase 1 [Source:HGNC Symbol;Acc:3769]	2.47	1.3
ENSG00000211659	IGLV3-25	immunoglobulin lambda variable 3-25 [Source:HGNC Symbol;Acc:5908]	2.46	1.3
ENSG00000173432	SAA1	serum amyloid A1 [Source:HGNC Symbol;Acc:10513]	2.46	1.3
ENSG00000016082	ISL1	ISL LIM homeobox 1 [Source:HGNC Symbol;Acc:6132]	2.44	1.29
ENSG00000167653	PSCA	prostate stem cell antigen [Source:HGNC Symbol;Acc:9500]	2.44	1.29
ENSG00000109193	SULT1E1	sulfotransferase family 1E. estrogen-preferring. member 1 [Source:HGNC Symbol;Acc:11377]	2.43	1.28
ENSG00000170323	FABP4	fatty acid binding protein 4. adipocyte [Source:HGNC Symbol;Acc:3559]	2.42	1.28
ENSG00000215834	FMO9P	flavin containing monooxygenase 9 pseudogene [Source:HGNC Symbol;Acc:32210]	2.42	1.27
ENSG00000211644	IGLV1-51	immunoglobulin lambda variable 1-51 [Source:HGNC Symbol;Acc:5882]	2.41	1.27
ENSG00000258545			2.37	1.24
ENSG00000224769			2.37	1.24
ENSG00000218357		Novel protein; Uncharacterized protein [Source:UniProtKB/TrEMBL;Acc:B1AJR3]	2.36	1.24
ENSG00000105369	CD79A	CD79a molecule. immunoglobulin-associated alpha [Source:HGNC Symbol;Acc:1698]	2.36	1.24
ENSG00000181143	MUC16	mucin 16. cell surface associated [Source:HGNC Symbol;Acc:15582]	2.36	1.24
ENSG00000235097	LINC00330	long intergenic non-protein coding RNA 330 [Source:HGNC Symbol;Acc:42047]	2.35	1.23
ENSG00000103316	CRYM	crystallin. mu [Source:HGNC Symbol;Acc:2418]	2.34	1.23
ENSG00000242317			2.34	1.23
ENSG00000079385	CEACAM1	carcinoembryonic antigen-related cell adhesion molecule 1 (biliary glycoprotein) [Source:HGNC Symbol;Acc:1814]	2.32	1.22
ENSG00000134827	TCN1	transcobalamin I (vitamin B12 binding protein. R binder family) [Source:HGNC Symbol;Acc:11652]	2.31	1.21
ENSG00000105929	ATP6V0A4	ATPase. H+ transporting. lysosomal V0 subunit a4 [Source:HGNC Symbol;Acc:866]	2.28	1.19
ENSG00000112303	VNN2	vanin 2 [Source:HGNC Symbol;Acc:12706]	2.28	1.19

ENSG00000153292	GPR110	G protein-coupled receptor 110 [Source:HGNC Symbol;Acc:18990]	2.28	1.19
ENSG00000073605	GSDMB	gasdermin B [Source:HGNC Symbol;Acc:23690]	2.27	1.19
ENSG00000004468	CD38	CD38 molecule [Source:HGNC Symbol;Acc:1667]	2.25	1.17
ENSG00000016602	CLCA4	chloride channel accessory 4 [Source:HGNC Symbol;Acc:2018]	2.24	1.17
ENSG00000196167	COLCA1	colorectal cancer associated 1 [Source:HGNC Symbol;Acc:33789]	2.24	1.16
ENSG00000261175			2.23	1.16
ENSG00000239819	IGKV1D-8	immunoglobulin kappa variable 1D-8 [Source:HGNC Symbol;Acc:5759]	2.22	1.15
ENSG00000249096			2.2	1.14
ENSG00000080031	PTPRH	protein tyrosine phosphatase. receptor type. H [Source:HGNC Symbol;Acc:9672]	2.19	1.13
ENSG00000090104	RGS1	regulator of G-protein signaling 1 [Source:HGNC Symbol;Acc:9991]	2.18	1.12
ENSG00000159618	GPR114	G protein-coupled receptor 114 [Source:HGNC Symbol;Acc:19010]	2.18	1.12
ENSG00000179913	B3GNT3	UDP-GlcNAc:betaGal beta-1.3-N-acetylglucosaminyltransferase 3 [Source:HGNC Symbol;Acc:13528]	2.16	1.11
ENSG00000103569	AQP9	aquaporin 9 [Source:HGNC Symbol;Acc:643]	2.15	1.11
ENSG00000268621			2.15	1.1
ENSG00000171236	LRG1	leucine-rich alpha-2-glycoprotein 1 [Source:HGNC Symbol;Acc:29480]	2.15	1.1
ENSG00000241351	IGKV3-11	immunoglobulin kappa variable 3-11 [Source:HGNC Symbol;Acc:5815]	2.14	1.1
ENSG00000096006	CRISP3	cysteine-rich secretory protein 3 [Source:HGNC Symbol;Acc:16904]	2.14	1.1
ENSG00000086548	CEACAM6	carcinoembryonic antigen-related cell adhesion molecule 6 (non-specific cross reacting antigen) [Source:HGNC Symbol;Acc:1818]	2.13	1.09
ENSG00000233999	IGKV3OR2-268	immunoglobulin kappa variable 3/OR2-268 (non-functional) [Source:HGNC Symbol;Acc:5830]	2.13	1.09
ENSG00000124731	TREM1	triggering receptor expressed on myeloid cells 1 [Source:HGNC Symbol;Acc:17760]	2.11	1.08
ENSG00000140379	BCL2A1	BCL2-related protein A1 [Source:HGNC Symbol;Acc:991]	2.11	1.08
ENSG00000112299	VNN1	vanin 1 [Source:HGNC Symbol;Acc:12705]	2.1	1.07
ENSG00000081041	CXCL2	chemokine (C-X-C motif) ligand 2 [Source:HGNC Symbol;Acc:4603]	2.09	1.06
ENSG00000094755	GABRP	gamma-aminobutyric acid (GABA) A receptor. pi [Source:HGNC Symbol;Acc:4089]	2.09	1.06
ENSG00000104371	DKK4	dickkopf WNT signaling pathway inhibitor 4 [Source:HGNC Symbol;Acc:2894]	2.09	1.06

ENSG00000007306	CEACAM7	carcinoembryonic antigen-related cell adhesion molecule 7 [Source:HGNC Symbol;Acc:1819]	2.09	1.06
ENSG00000171051	FPR1	formyl peptide receptor 1 [Source:HGNC Symbol;Acc:3826]	2.07	1.05
ENSG00000140297	GCNT3	glucosaminyl (N-acetyl) transferase 3. mucin type [Source:HGNC Symbol;Acc:4205]	2.06	1.05
ENSG00000162873	KLHDC8A	kelch domain containing 8A [Source:HGNC Symbol;Acc:25573]	2.06	1.05
ENSG00000115523	GNLY	granulysin [Source:HGNC Symbol;Acc:4414]	2.06	1.04
ENSG00000237683		Uncharacterized protein [Source:UniProtKB/TrEMBL;Acc:R4GN28]	2.05	1.04
ENSG00000129514	FOXA1	forkhead box A1 [Source:HGNC Symbol;Acc:5021]	2.04	1.03
ENSG00000042980	ADAM28	ADAM metallopeptidase domain 28 [Source:HGNC Symbol;Acc:206]	2.03	1.02
ENSG00000165685	TMEM52B	transmembrane protein 52B [Source:HGNC Symbol;Acc:26438]	2.03	1.02
ENSG00000187527	ATP13A5	ATPase type 13A5 [Source:HGNC Symbol;Acc:31789]	2.02	1.02
ENSG00000113070	HBEGF	heparin-binding EGF-like growth factor [Source:HGNC Symbol;Acc:3059]	2.02	1.02
ENSG00000105289	TJP3	tight junction protein 3 [Source:HGNC Symbol;Acc:11829]	2.02	1.02
ENSG00000187116	LILRA5	leukocyte immunoglobulin-like receptor. subfamily A (with TM domain). member 5 [Source:HGNC Symbol;Acc:16309]	2.00	1.00
ENSG00000124143	ARHGAP40	Rho GTPase activating protein 40 [Source:HGNC Symbol;Acc:16226]	2.00	1.00
ENSG00000214290	COLCA2	colorectal cancer associated 2 [Source:HGNC Symbol;Acc:26978]	1.99	0.99
ENSG00000239571	IGKV2D-30	immunoglobulin kappa variable 2D-30 [Source:HGNC Symbol;Acc:5801]	1.99	0.99
ENSG00000248663	LINC00992	long intergenic non-protein coding RNA 992 [Source:HGNC Symbol;Acc:48943]	1.99	0.99
ENSG00000243466	IGKV1-5	immunoglobulin kappa variable 1-5 [Source:HGNC Symbol;Acc:5741]	1.98	0.99
ENSG00000086696	HSD17B2	hydroxysteroid (17-beta) dehydrogenase 2 [Source:HGNC Symbol;Acc:5211]	1.97	0.98
ENSG00000167772	ANGPTL4	angiopoietin-like 4 [Source:HGNC Symbol;Acc:16039]	1.97	0.97
ENSG00000075035	WSCD2	WSC domain containing 2 [Source:HGNC Symbol;Acc:29117]	1.96	0.97
ENSG00000148346	LCN2	lipocalin 2 [Source:HGNC Symbol;Acc:6526]	1.96	0.97
ENSG00000164691	TAGAP	T-cell activation RhoGTPase activating protein [Source:HGNC Symbol;Acc:15669]	1.96	0.97
ENSG00000168702	LRP1B	low density lipoprotein receptor-related protein 1B [Source:HGNC Symbol;Acc:6693]	1.96	0.97
ENSG00000255398	HCAR3	hydroxycarboxylic acid receptor 3 [Source:HGNC Symbol;Acc:16824]	1.95	0.96
ENSG00000002587	HS3ST1	heparan sulfate (glucosamine) 3-O-sulfotransferase 1 [Source:HGNC Symbol;Acc:5194]	1.93	0.95

ENSG00000158125	XDH	xanthine dehydrogenase [Source:HGNC Symbol;Acc:12805]	1.93	0.95
ENSG00000099958	DERL3	derlin 3 [Source:HGNC Symbol;Acc:14236]	1.92	0.94
ENSG00000236915			1.92	0.94
ENSG00000230373	GOLGA6L5P	golgin A6 family-like 5. pseudogene [Source:HGNC Symbol;Acc:30472]	1.92	0.94
ENSG00000162747	FCGR3B	Fc fragment of IgG. low affinity IIIb. receptor (CD16b) [Source:HGNC Symbol;Acc:3620]	1.91	0.94
ENSG00000135116	HRK	harakiri. BCL2 interacting protein (contains only BH3 domain) [Source:HGNC Symbol;Acc:5185]	1.91	0.93
ENSG00000019102	VSIG2	V-set and immunoglobulin domain containing 2 [Source:HGNC Symbol;Acc:17149]	1.9	0.93
ENSG00000170345	FOS	FBJ murine osteosarcoma viral oncogene homolog [Source:HGNC Symbol;Acc:3796]	1.9	0.93
ENSG00000072858	SIDT1	SID1 transmembrane family. member 1 [Source:HGNC Symbol;Acc:25967]	1.9	0.92
ENSG00000104974	LILRA1	leukocyte immunoglobulin-like receptor. subfamily A (with TM domain). member 1 [Source:HGNC Symbol;Acc:6602]	1.9	0.92
ENSG00000153234	NR4A2	nuclear receptor subfamily 4. group A. member 2 [Source:HGNC Symbol;Acc:7981]	1.89	0.92
ENSG00000163874	ZC3H12A	zinc finger CCCH-type containing 12A [Source:HGNC Symbol;Acc:26259]	1.89	0.92
ENSG00000115919	KYNU	kynureninase [Source:HGNC Symbol;Acc:6469]	1.89	0.92
ENSG00000124920	MYRF	myelin regulatory factor [Source:HGNC Symbol;Acc:1181]	1.89	0.92
ENSG00000153923	CLCA3P	chloride channel accessory 3. pseudogene [Source:HGNC Symbol;Acc:2017]	1.89	0.92
ENSG00000260402			1.89	0.92
ENSG00000158286	RNF207	ring finger protein 207 [Source:HGNC Symbol;Acc:32947]	1.89	0.91
ENSG00000197249	SERPINA1	serpin peptidase inhibitor. clade A (alpha-1 antiproteinase. antitrypsin). member 1 [Source:HGNC Symbol;Acc:8941]	1.88	0.91
ENSG00000172828	CES3	carboxylesterase 3 [Source:HGNC Symbol;Acc:1865]	1.88	0.91
ENSG00000175147	TMEM51-AS1	TMEM51 antisense RNA 1 [Source:HGNC Symbol;Acc:26301]	1.88	0.91
ENSG00000273301			1.88	0.91
ENSG00000111291	GPRC5D	G protein-coupled receptor. family C. group 5. member D [Source:HGNC Symbol;Acc:13310]	1.88	0.91
ENSG00000258077			1.88	0.91
ENSG00000115607	IL18RAP	interleukin 18 receptor accessory protein [Source:HGNC Symbol;Acc:5989]	1.87	0.91
ENSG00000119938	PPP1R3C	protein phosphatase 1. regulatory subunit 3C [Source:HGNC Symbol;Acc:9293]	1.86	0.9

ENSG00000184669	OR7E14P	olfactory receptor. family 7. subfamily E. member 14 pseudogene [Source:HGNC Symbol;Acc:8385]	1.86	0.89
ENSG00000124256	ZBP1	Z-DNA binding protein 1 [Source:HGNC Symbol;Acc:16176]	1.86	0.89
ENSG00000136231	IGF2BP3	insulin-like growth factor 2 mRNA binding protein 3 [Source:HGNC Symbol;Acc:28868]	1.84	0.88
ENSG00000130768	SMPDL3B	sphingomyelin phosphodiesterase. acid-like 3B [Source:HGNC Symbol;Acc:21416]	1.84	0.88
ENSG00000196684	HSH2D	hematopoietic SH2 domain containing [Source:HGNC Symbol;Acc:24920]	1.84	0.88
ENSG00000008513	ST3GAL1	ST3 beta-galactoside alpha-2.3-sialyltransferase 1 [Source:HGNC Symbol;Acc:10862]	1.84	0.88
ENSG00000122877	EGR2	early growth response 2 [Source:HGNC Symbol;Acc:3239]	1.84	0.88
ENSG00000234678			1.83	0.87
ENSG00000205403	CFI	complement factor I [Source:HGNC Symbol;Acc:5394]	1.83	0.87
ENSG00000198574	SH2D1B	SH2 domain containing 1B [Source:HGNC Symbol;Acc:30416]	1.83	0.87
ENSG00000163406	SLC15A2	solute carrier family 15 (oligopeptide transporter). member 2 [Source:HGNC Symbol;Acc:10921]	1.82	0.87
ENSG00000077092	RARB	retinoic acid receptor. beta [Source:HGNC Symbol;Acc:9865]	1.82	0.86
ENSG00000162931	TRIM17	tripartite motif containing 17 [Source:HGNC Symbol;Acc:13430]	1.81	0.86
ENSG00000134242	PTPN22	protein tyrosine phosphatase. non-receptor type 22 (lymphoid) [Source:HGNC Symbol;Acc:9652]	1.81	0.85
ENSG00000232680			1.8	0.85
ENSG00000156966	B3GNT7	UDP-GlcNAc:betaGal beta-1.3-N-acetylglucosaminyltransferase 7 [Source:HGNC Symbol;Acc:18811]	1.8	0.84
ENSG00000005001	PRSS22	protease. serine. 22 [Source:HGNC Symbol;Acc:14368]	1.79	0.84
ENSG00000143369	ECM1	extracellular matrix protein 1 [Source:HGNC Symbol;Acc:3153]	1.78	0.83
ENSG00000105388	CEACAM5	carcinoembryonic antigen-related cell adhesion molecule 5 [Source:HGNC Symbol;Acc:1817]	1.78	0.83
ENSG00000214145	LINC00887	long intergenic non-protein coding RNA 887 [Source:HGNC Symbol;Acc:48574]	1.78	0.83
ENSG00000231412			1.78	0.83
ENSG00000064655	EYA2	eyes absent homolog 2 (Drosophila) [Source:HGNC Symbol;Acc:3520]	1.77	0.82
ENSG00000128285	MCHR1	melanin-concentrating hormone receptor 1 [Source:HGNC Symbol;Acc:4479]	1.77	0.82
ENSG00000248485	PCP4L1	Purkinje cell protein 4 like 1 [Source:HGNC Symbol;Acc:20448]	1.77	0.82
ENSG00000099954	CECR2	cat eye syndrome chromosome region. candidate 2 [Source:HGNC Symbol;Acc:1840]	1.76	0.82
ENSG00000139572	GPR84	G protein-coupled receptor 84 [Source:HGNC Symbol;Acc:4535]	1.76	0.81

ENSG00000214425	LRRC37A4P	leucine rich repeat containing 37. member A4. pseudogene [Source:HGNC Symbol;Acc:25479]	1.75	0.81
ENSG00000239998	LILRA2	leukocyte immunoglobulin-like receptor. subfamily A (with TM domain). member 2 [Source:HGNC Symbol;Acc:6603]	1.75	0.81
ENSG00000170835	CEL	carboxyl ester lipase [Source:HGNC Symbol;Acc:1848]	1.75	0.81
ENSG00000184012	TMPRSS2	transmembrane protease. serine 2 [Source:HGNC Symbol;Acc:11876]	1.75	0.81
ENSG00000139289	PHLDA1	pleckstrin homology-like domain. family A. member 1 [Source:HGNC Symbol;Acc:8933]	1.75	0.8
ENSG00000127507	EMR2	egf-like module containing. mucin-like. hormone receptor-like 2 [Source:HGNC Symbol;Acc:3337]	1.74	0.8
ENSG00000149260	CAPN5	calpain 5 [Source:HGNC Symbol;Acc:1482]	1.74	0.8
ENSG00000184254	ALDH1A3	aldehyde dehydrogenase 1 family. member A3 [Source:HGNC Symbol;Acc:409]	1.74	0.8
ENSG00000179593	ALOX15B	arachidonate 15-lipoxygenase. type B [Source:HGNC Symbol;Acc:434]	1.74	0.8
ENSG00000104368	PLAT	plasminogen activator. tissue [Source:HGNC Symbol;Acc:9051]	1.74	0.8
ENSG00000162078	ZG16B	zymogen granule protein 16B [Source:HGNC Symbol;Acc:30456]	1.74	0.79
ENSG00000132182	NUP210	nucleoporin 210kDa [Source:HGNC Symbol;Acc:30052]	1.73	0.79
ENSG00000205744	DENND1C	DENN/MADD domain containing 1C [Source:HGNC Symbol;Acc:26225]	1.73	0.79
ENSG00000136694	IL36A	interleukin 36. alpha [Source:HGNC Symbol;Acc:15562]	1.72	0.78
ENSG00000214049	UCA1	urothelial cancer associated 1 (non-protein coding) [Source:HGNC Symbol;Acc:37126]	1.72	0.78
ENSG00000163435	ELF3	E74-like factor 3 (ets domain transcription factor. epithelial-specific) [Source:HGNC Symbol;Acc:3318]	1.71	0.78
ENSG00000172927	MYEOV	myeloma overexpressed [Source:HGNC Symbol;Acc:7563]	1.71	0.77
ENSG00000164707	SLC13A4	solute carrier family 13 (sodium/sulfate symporter). member 4 [Source:HGNC Symbol;Acc:15827]	1.7	0.77
ENSG00000143382	ADAMTSL4	ADAMTS-like 4 [Source:HGNC Symbol;Acc:19706]	1.7	0.76
ENSG00000204544	MUC21	mucin 21. cell surface associated [Source:HGNC Symbol;Acc:21661]	1.7	0.76
ENSG00000235576			1.7	0.76
ENSG00000166920	C15orf48	chromosome 15 open reading frame 48 [Source:HGNC Symbol;Acc:29898]	1.69	0.76
ENSG00000138166	DUSP5	dual specificity phosphatase 5 [Source:HGNC Symbol;Acc:3071]	1.69	0.75
ENSG00000176945	MUC20	mucin 20. cell surface associated [Source:HGNC Symbol;Acc:23282]	1.69	0.75
ENSG00000101307	SIRPB1	signal-regulatory protein beta 1 [Source:HGNC Symbol;Acc:15928]	1.68	0.75

ENSG00000254109	RBPMS-AS1	RBPMS antisense RNA 1 [Source:HGNC Symbol;Acc:48721]	1.67	0.74
ENSG00000180644	PRF1	perforin 1 (pore forming protein) [Source:HGNC Symbol;Acc:9360]	1.67	0.74
ENSG00000261971			1.67	0.74
ENSG00000261804			1.67	0.74
ENSG00000172602	RND1	Rho family GTPase 1 [Source:HGNC Symbol;Acc:18314]	1.66	0.73
ENSG00000140519	RHCG	Rh family. C glycoprotein [Source:HGNC Symbol;Acc:18140]	1.65	0.73
ENSG00000145283	SLC10A6	solute carrier family 10 (sodium/bile acid cotransporter). member 6 [Source:HGNC Symbol;Acc:30603]	1.65	0.72
ENSG00000198643	FAM3D	family with sequence similarity 3. member D [Source:HGNC Symbol;Acc:18665]	1.65	0.72
ENSG00000167608	TMC4	transmembrane channel-like 4 [Source:HGNC Symbol;Acc:22998]	1.64	0.72
ENSG00000069535	MAOB	monoamine oxidase B [Source:HGNC Symbol;Acc:6834]	1.64	0.72
ENSG00000157111	TMEM171	transmembrane protein 171 [Source:HGNC Symbol;Acc:27031]	1.64	0.72
ENSG00000128016	ZFP36	ZFP36 ring finger protein [Source:HGNC Symbol;Acc:12862]	1.64	0.71
ENSG00000211663	IGLV3-19	immunoglobulin lambda variable 3-19 [Source:HGNC Symbol;Acc:5903]	1.64	0.71
ENSG00000116741	RGS2	regulator of G-protein signaling 2. 24kDa [Source:HGNC Symbol;Acc:9998]	1.64	0.71
ENSG00000233947			1.63	0.71
ENSG00000120756	PLS1	plastin 1 [Source:HGNC Symbol;Acc:9090]	1.63	0.71
ENSG00000065923	SLC9A7	solute carrier family 9. subfamily A (NHE7. cation proton antiporter 7). member 7 [Source:HGNC Symbol;Acc:17123]	1.63	0.71
ENSG00000072274	TFRC	transferrin receptor [Source:HGNC Symbol;Acc:11763]	1.63	0.7
ENSG00000129646	QRICH2	glutamine rich 2 [Source:HGNC Symbol;Acc:25326]	1.62	0.7
ENSG00000254750	CASP1P2	caspase 1. apoptosis-related cysteine peptidase pseudogene 2 [Source:HGNC Symbol;Acc:43776]	1.62	0.7
ENSG00000163898	LIPH	lipase. member H [Source:HGNC Symbol;Acc:18483]	1.62	0.69
ENSG00000167851	CD300A	CD300a molecule [Source:HGNC Symbol;Acc:19319]	1.61	0.69
ENSG00000130775	THEMIS2	thymocyte selection associated family member 2 [Source:HGNC Symbol;Acc:16839]	1.61	0.69
ENSG00000160190	SLC37A1	solute carrier family 37 (glucose-6-phosphate transporter). member 1 [Source:HGNC Symbol;Acc:11024]	1.61	0.69
ENSG00000178690	DYNAP	dynactin associated protein [Source:HGNC Symbol;Acc:26808]	1.61	0.69

ENSG00000156413	FUT6	fucosyltransferase 6 (alpha (1.3) fucosyltransferase) [Source:HGNC Symbol;Acc:4017]	1.61	0.68
ENSG00000196730	DAPK1	death-associated protein kinase 1 [Source:HGNC Symbol;Acc:2674]	1.6	0.68
ENSG00000267709			1.6	0.68
ENSG00000115956	PLEK	pleckstrin [Source:HGNC Symbol;Acc:9070]	1.6	0.67
ENSG00000064687	ABCA7	ATP-binding cassette. sub-family A (ABC1). member 7 [Source:HGNC Symbol;Acc:37]	1.59	0.67
ENSG00000158517	NCF1	neutrophil cytosolic factor 1 [Source:HGNC Symbol;Acc:7660]	1.59	0.67
ENSG00000121236	TRIM6	tripartite motif containing 6 [Source:HGNC Symbol;Acc:16277]	1.59	0.66
ENSG00000163687	DNASE1L3	deoxyribonuclease I-like 3 [Source:HGNC Symbol;Acc:2959]	1.59	0.66
ENSG00000084734	GCKR	glucokinase (hexokinase 4) regulator [Source:HGNC Symbol;Acc:4196]	1.58	0.66
ENSG00000244040	IL12A-AS1	IL12A antisense RNA 1 [Source:HGNC Symbol;Acc:49094]	1.58	0.66
ENSG00000140459	CYP11A1	cytochrome P450. family 11. subfamily A. polypeptide 1 [Source:HGNC Symbol;Acc:2590]	1.58	0.66
ENSG00000105409	ATP1A3	ATPase. Na+/K+ transporting. alpha 3 polypeptide [Source:HGNC Symbol;Acc:801]	1.58	0.66
ENSG00000124593	PRICKLE4	prickle homolog 4 (Drosophila) [Source:HGNC Symbol;Acc:16805]	1.58	0.66
ENSG00000134324	LPIN1	lipin 1 [Source:HGNC Symbol;Acc:13345]	1.57	0.65
ENSG00000121064	SCPEP1	serine carboxypeptidase 1 [Source:HGNC Symbol;Acc:29507]	1.57	0.65
ENSG00000135114	OASL	2'-5'-oligoadenylate synthetase-like [Source:HGNC Symbol;Acc:8090]	1.57	0.65
ENSG00000181885	CLDN7	claudin 7 [Source:HGNC Symbol;Acc:2049]	1.57	0.65
ENSG00000126262	FFAR2	free fatty acid receptor 2 [Source:HGNC Symbol;Acc:4501]	1.57	0.65
ENSG00000054690	PLEKHH1	pleckstrin homology domain containing. family H (with MyTH4 domain) member 1 [Source:HGNC Symbol;Acc:17733]	1.57	0.65
ENSG00000125657	TNFSF9	tumor necrosis factor (ligand) superfamily. member 9 [Source:HGNC Symbol;Acc:11939]	1.56	0.65
ENSG00000108950	FAM20A	family with sequence similarity 20. member A [Source:HGNC Symbol;Acc:23015]	1.56	0.64
ENSG00000144802	NFKBIZ	nuclear factor of kappa light polypeptide gene enhancer in B-cells inhibitor. zeta [Source:HGNC Symbol;Acc:29805]	1.56	0.64
ENSG00000186074	CD300LF	CD300 molecule-like family member f [Source:HGNC Symbol;Acc:29883]	1.56	0.64
ENSG00000121446	RGSL1	regulator of G-protein signaling like 1 [Source:HGNC Symbol;Acc:18636]	1.56	0.64
ENSG00000227619			1.56	0.64

ENSG00000254087	LYN	v-yes-1 Yamaguchi sarcoma viral related oncogene homolog [Source:HGNC Symbol;Acc:6735]	1.56	0.64
ENSG00000171345	KRT19	keratin 19 [Source:HGNC Symbol;Acc:6436]	1.56	0.64
ENSG00000078081	LAMP3	lysosomal-associated membrane protein 3 [Source:HGNC Symbol;Acc:14582]	1.55	0.63
ENSG00000094963	FMO2	flavin containing monooxygenase 2 (non-functional) [Source:HGNC Symbol;Acc:3770]	1.55	0.63
ENSG00000135838	NPL	N-acetylneuraminate pyruvate lyase (dihydrodipicolinate synthase) [Source:HGNC Symbol;Acc:16781]	1.54	0.63
ENSG00000143536	CRNN	cornulin [Source:HGNC Symbol;Acc:1230]	1.54	0.62
ENSG00000250103			1.54	0.62
ENSG00000188761	BCL2L15	BCL2-like 15 [Source:HGNC Symbol;Acc:33624]	1.54	0.62
ENSG00000140534	TICRR	TOPBP1-interacting checkpoint and replication regulator [Source:HGNC Symbol;Acc:28704]	1.53	0.62
ENSG00000197416	FABP12	fatty acid binding protein 12 [Source:HGNC Symbol;Acc:34524]	1.53	0.61
ENSG00000154165	GPR15	G protein-coupled receptor 15 [Source:HGNC Symbol;Acc:4469]	1.53	0.61
ENSG00000161265	U2AF1L4	U2 small nuclear RNA auxiliary factor 1-like 4 [Source:HGNC Symbol;Acc:23020]	1.53	0.61
ENSG00000147168	IL2RG	interleukin 2 receptor. gamma [Source:HGNC Symbol;Acc:6010]	1.52	0.61
ENSG00000178723	GLULP4	glutamate-ammonia ligase (glutamine synthetase) pseudogene 4 [Source:HGNC Symbol;Acc:4345]	1.52	0.61
ENSG00000170298	LGALS9B	lectin. galactoside-binding. soluble. 9B [Source:HGNC Symbol;Acc:24842]	1.52	0.61
ENSG00000006059	KRT33A	keratin 33A [Source:HGNC Symbol;Acc:6450]	1.52	0.6
ENSG00000060140	STYK1	serine/threonine/tyrosine kinase 1 [Source:HGNC Symbol;Acc:18889]	1.52	0.6
ENSG00000214331			1.52	0.6
ENSG00000134539	KLRD1	killer cell lectin-like receptor subfamily D. member 1 [Source:HGNC Symbol;Acc:6378]	1.52	0.6
ENSG00000269899			1.51	0.6
ENSG00000179163	FUCA1	fucosidase. alpha-L- 1. tissue [Source:HGNC Symbol;Acc:4006]	1.51	0.6
ENSG00000261040			1.51	0.6
ENSG00000221963	APOL6	apolipoprotein L. 6 [Source:HGNC Symbol;Acc:14870]	1.51	0.6
ENSG00000072954	TMEM38A	transmembrane protein 38A [Source:HGNC Symbol;Acc:28462]	1.51	0.59
ENSG00000150551	LYPD1	LY6/PLAUR domain containing 1 [Source:HGNC Symbol;Acc:28431]	1.5	0.59
ENSG00000144959	NCEH1	neutral cholesterol ester hydrolase 1 [Source:HGNC Symbol;Acc:29260]	1.5	0.58

ENSG00000116514	RNF19B	ring finger protein 19B [Source:HGNC Symbol;Acc:26886]	1.5	0.58
ENSG00000173805	HAP1	huntingtin-associated protein 1 [Source:HGNC Symbol;Acc:4812]	1.49	0.57
ENSG00000106688	SLC1A1	solute carrier family 1 (neuronal/epithelial high affinity glutamate transporter. system Xag). member 1 [Source:HGNC Symbol;Acc:10939]	1.49	0.57
ENSG00000143153	ATP1B1	ATPase. Na+/K+ transporting. beta 1 polypeptide [Source:HGNC Symbol;Acc:804]	1.49	0.57
ENSG00000143850	PLEKHA6	pleckstrin homology domain containing. family A member 6 [Source:HGNC Symbol;Acc:17053]	1.49	0.57
ENSG00000197142	ACSL5	acyl-CoA synthetase long-chain family member 5 [Source:HGNC Symbol;Acc:16526]	1.49	0.57
ENSG00000154556	SORBS2	sorbin and SH3 domain containing 2 [Source:HGNC Symbol;Acc:24098]	1.49	0.57
ENSG00000189377	CXCL17	chemokine (C-X-C motif) ligand 17 [Source:HGNC Symbol;Acc:19232]	1.48	0.57
ENSG00000198223	CSF2RA	colony stimulating factor 2 receptor. alpha. low-affinity (granulocyte-macrophage) [Source:HGNC Symbol;Acc:2435]	1.48	0.57
ENSG00000162654	GBP4	guanylate binding protein 4 [Source:HGNC Symbol;Acc:20480]	1.48	0.57
ENSG00000240859			1.48	0.57
ENSG00000177409	SAMD9L	sterile alpha motif domain containing 9-like [Source:HGNC Symbol;Acc:1349]	1.48	0.57
ENSG00000250511			1.48	0.56
ENSG00000140284	SLC27A2	solute carrier family 27 (fatty acid transporter). member 2 [Source:HGNC Symbol;Acc:10996]	1.48	0.56
ENSG00000183208	GDPGP1	GDP-D-glucose phosphorylase 1 [Source:HGNC Symbol;Acc:34360]	1.47	0.56
ENSG00000100342	APOL1	apolipoprotein L. 1 [Source:HGNC Symbol;Acc:618]	1.47	0.56
ENSG00000233834			1.47	0.55
ENSG00000234262			1.47	0.55
ENSG00000156675	RAB11FIP1	RAB11 family interacting protein 1 (class I) [Source:HGNC Symbol;Acc:30265]	1.47	0.55
ENSG00000134326	CMPK2	cytidine monophosphate (UMP-CMP) kinase 2. mitochondrial [Source:HGNC Symbol;Acc:27015]	1.46	0.55
ENSG00000186377	CYP4X1	cytochrome P450. family 4. subfamily X. polypeptide 1 [Source:HGNC Symbol;Acc:20244]	1.46	0.55
ENSG00000243069	ARHGEF26-AS1	ARHGEF26 antisense RNA 1 [Source:HGNC Symbol;Acc:41048]	1.46	0.55
ENSG00000012779	ALOX5	arachidonate 5-lipoxygenase [Source:HGNC Symbol;Acc:435]	1.46	0.54
ENSG00000069399	BCL3	B-cell CLL/lymphoma 3 [Source:HGNC Symbol;Acc:998]	1.46	0.54
ENSG00000135476	ESPL1	extra spindle pole bodies homolog 1 (S. cerevisiae) [Source:HGNC Symbol;Acc:16856]	1.46	0.54

ENSG00000167759	KLK13	kallikrein-related peptidase 13 [Source:HGNC Symbol;Acc:6361]	1.46	0.54
ENSG00000173193	PARP14	poly (ADP-ribose) polymerase family. member 14 [Source:HGNC Symbol;Acc:29232]	1.45	0.54
ENSG00000057657	PRDM1	PR domain containing 1. with ZNF domain [Source:HGNC Symbol;Acc:9346]	1.45	0.54
ENSG00000143507	DUSP10	dual specificity phosphatase 10 [Source:HGNC Symbol;Acc:3065]	1.45	0.54
ENSG00000117335	CD46	CD46 molecule. complement regulatory protein [Source:HGNC Symbol;Acc:6953]	1.45	0.53
ENSG00000163959	SLC51A	solute carrier family 51. alpha subunit [Source:HGNC Symbol;Acc:29955]	1.45	0.53
ENSG00000185761	ADAMTSL5	ADAMTS-like 5 [Source:HGNC Symbol;Acc:27912]	1.45	0.53
ENSG00000186529	CYP4F3	cytochrome P450. family 4. subfamily F. polypeptide 3 [Source:HGNC Symbol;Acc:2646]	1.44	0.53
ENSG00000236893	ASS1P7	argininosuccinate synthetase 1 pseudogene 7 [Source:HGNC Symbol;Acc:770]	1.44	0.53
ENSG00000008086	CDKL5	cyclin-dependent kinase-like 5 [Source:HGNC Symbol;Acc:11411]	1.44	0.53
ENSG00000132530	XAF1	XIAP associated factor 1 [Source:HGNC Symbol;Acc:30932]	1.44	0.53
ENSG00000135960	EDAR	ectodysplasin A receptor [Source:HGNC Symbol;Acc:2895]	1.44	0.53
ENSG00000122359	ANXA11	annexin A11 [Source:HGNC Symbol;Acc:535]	1.44	0.53
ENSG00000166979	EVA1C	eva-1 homolog C (C. elegans) [Source:HGNC Symbol;Acc:13239]	1.44	0.53
ENSG00000137965	IFI44	interferon-induced protein 44 [Source:HGNC Symbol;Acc:16938]	1.44	0.52
ENSG00000088367	EPB41L1	erythrocyte membrane protein band 4.1-like 1 [Source:HGNC Symbol;Acc:3378]	1.43	0.52
ENSG00000188641	DPYD	dihydropyrimidine dehydrogenase [Source:HGNC Symbol;Acc:3012]	1.43	0.52
ENSG00000230039			1.43	0.52
ENSG00000135821	GLUL	glutamate-ammonia ligase [Source:HGNC Symbol;Acc:4341]	1.43	0.52
ENSG00000179299	NSUN7	NOP2/Sun domain family. member 7 [Source:HGNC Symbol;Acc:25857]	1.43	0.51
ENSG00000198807	PAX9	paired box 9 [Source:HGNC Symbol;Acc:8623]	1.43	0.51
ENSG00000100219	XBP1	X-box binding protein 1 [Source:HGNC Symbol;Acc:12801]	1.42	0.5
ENSG00000130707	ASS1	argininosuccinate synthase 1 [Source:HGNC Symbol;Acc:758]	1.42	0.5
ENSG00000250479	CHCHD10	coiled-coil-helix-coiled-coil-helix domain containing 10 [Source:HGNC Symbol;Acc:15559]	1.42	0.5
ENSG00000101850	GPR143	G protein-coupled receptor 143 [Source:HGNC Symbol;Acc:20145]	1.41	0.5
ENSG00000132965	ALOX5AP	arachidonate 5-lipoxygenase-activating protein [Source:HGNC Symbol;Acc:436]	1.41	0.5

ENSG00000186472	PCLO	piccolo presynaptic cytomatrix protein [Source:HGNC Symbol;Acc:13406]	1.41	0.5
ENSG00000121039	RDH10	retinol dehydrogenase 10 (all-trans) [Source:HGNC Symbol;Acc:19975]	1.41	0.5
ENSG00000151575	TEX9	testis expressed 9 [Source:HGNC Symbol;Acc:29585]	1.41	0.5
ENSG00000130066	SAT1	spermidine/spermine N1-acetyltransferase 1 [Source:HGNC Symbol;Acc:10540]	1.41	0.5
ENSG00000182752	PAPPA	pregnancy-associated plasma protein A. pappalysin 1 [Source:HGNC Symbol;Acc:8602]	1.41	0.5
ENSG00000188185	LINC00265	long intergenic non-protein coding RNA 265 [Source:HGNC Symbol;Acc:28019]	1.41	0.49
ENSG00000173890	GPR160	G protein-coupled receptor 160 [Source:HGNC Symbol;Acc:23693]	1.41	0.49
ENSG00000137648	TMPRSS4	transmembrane protease. serine 4 [Source:HGNC Symbol;Acc:11878]	1.4	0.49
ENSG00000148926	ADM	adrenomedullin [Source:HGNC Symbol;Acc:259]	1.4	0.48
ENSG00000147100	SLC16A2	solute carrier family 16. member 2 (thyroid hormone transporter) [Source:HGNC Symbol;Acc:10923]	1.4	0.48
ENSG00000172005	MAL	mal. T-cell differentiation protein [Source:HGNC Symbol;Acc:6817]	1.4	0.48
ENSG00000110080	ST3GAL4	ST3 beta-galactoside alpha-2.3-sialyltransferase 4 [Source:HGNC Symbol;Acc:10864]	1.39	0.48
ENSG00000117115	PADI2	peptidyl arginine deiminase. type II [Source:HGNC Symbol;Acc:18341]	1.39	0.48
ENSG00000138771	SHROOM3	shroom family member 3 [Source:HGNC Symbol;Acc:30422]	1.39	0.48
ENSG00000112699	GMDS	GDP-mannose 4.6-dehydratase [Source:HGNC Symbol;Acc:4369]	1.39	0.48
ENSG00000185215	TNFAIP2	tumor necrosis factor. alpha-induced protein 2 [Source:HGNC Symbol;Acc:11895]	1.39	0.48
ENSG00000134531	EMP1	epithelial membrane protein 1 [Source:HGNC Symbol;Acc:3333]	1.39	0.47
ENSG00000125648	SLC25A23	solute carrier family 25 (mitochondrial carrier; phosphate carrier). member 23 [Source:HGNC Symbol;Acc:19375]	1.39	0.47
ENSG00000046604	DSG2	desmoglein 2 [Source:HGNC Symbol;Acc:3049]	1.38	0.47
ENSG00000133321	RARRES3	retinoic acid receptor responder (tazarotene induced) 3 [Source:HGNC Symbol;Acc:9869]	1.38	0.47
ENSG00000101447	FAM83D	family with sequence similarity 83. member D [Source:HGNC Symbol;Acc:16122]	1.38	0.46
ENSG00000260711			1.38	0.46
ENSG00000170581	STAT2	signal transducer and activator of transcription 2. 113kDa [Source:HGNC Symbol;Acc:11363]	1.37	0.46
ENSG00000125772	GPCPD1	glycerophosphocholine phosphodiesterase GDE1 homolog (S. cerevisiae) [Source:HGNC Symbol;Acc:26957]	1.37	0.45
ENSG00000182010	RTKN2	rhotekin 2 [Source:HGNC Symbol;Acc:19364]	1.37	0.45

ENSG00000013588	GPRC5A	G protein-coupled receptor. family C. group 5. member A [Source:HGNC Symbol;Acc:9836]	1.37	0.45
ENSG00000167600	CYP2S1	cytochrome P450. family 2. subfamily S. polypeptide 1 [Source:HGNC Symbol;Acc:15654]	1.37	0.45
ENSG00000187650	VMAC	vimentin-type intermediate filament associated coiled-coil protein [Source:HGNC Symbol;Acc:33803]	1.37	0.45
ENSG00000054598	FOXC1	forkhead box C1 [Source:HGNC Symbol;Acc:3800]	1.36	0.45
ENSG00000120129	DUSP1	dual specificity phosphatase 1 [Source:HGNC Symbol;Acc:3064]	1.36	0.45
ENSG00000139531	SUOX	sulfite oxidase [Source:HGNC Symbol;Acc:11460]	1.36	0.45
ENSG00000125434	SLC25A35	solute carrier family 25. member 35 [Source:HGNC Symbol;Acc:31921]	1.36	0.45
ENSG00000197580	BCO2	beta-carotene oxygenase 2 [Source:HGNC Symbol;Acc:18503]	1.36	0.45
ENSG00000003400	CASP10	caspase 10. apoptosis-related cysteine peptidase [Source:HGNC Symbol;Acc:1500]	1.36	0.44
ENSG00000197930	ERO1L	ERO1-like (S. cerevisiae) [Source:HGNC Symbol;Acc:13280]	1.36	0.44
ENSG00000103226	NOMO3	NODAL modulator 3 [Source:HGNC Symbol;Acc:25242]	1.36	0.44
ENSG00000089327	FXYD5	FXYD domain containing ion transport regulator 5 [Source:HGNC Symbol;Acc:4029]	1.35	0.44
ENSG00000142627	EPHA2	EPH receptor A2 [Source:HGNC Symbol;Acc:3386]	1.35	0.44
ENSG00000272636	DOC2B	double C2-like domains. beta [Source:HGNC Symbol;Acc:2986]	1.35	0.44
ENSG00000115415	STAT1	signal transducer and activator of transcription 1. 91kDa [Source:HGNC Symbol;Acc:11362]	1.35	0.44
ENSG00000144655	CSRNP1	cysteine-serine-rich nuclear protein 1 [Source:HGNC Symbol;Acc:14300]	1.35	0.43
ENSG00000163209	SPRR3	small proline-rich protein 3 [Source:HGNC Symbol;Acc:11268]	1.35	0.43
ENSG00000166444	ST5	suppression of tumorigenicity 5 [Source:HGNC Symbol;Acc:11350]	1.35	0.43
ENSG00000130396	MLLT4	myeloid/lymphoid or mixed-lineage leukemia (trithorax homolog. Drosophila); translocated to. 4 [Source:HGNC Symbol;Acc:7137]	1.34	0.43
ENSG00000204138	PHACTR4	phosphatase and actin regulator 4 [Source:HGNC Symbol;Acc:25793]	1.34	0.42
ENSG00000115271	GCA	grancalcin. EF-hand calcium binding protein [Source:HGNC Symbol;Acc:15990]	1.34	0.42
ENSG00000112715	VEGFA	vascular endothelial growth factor A [Source:HGNC Symbol;Acc:12680]	1.34	0.42
ENSG00000134070	IRAK2	interleukin-1 receptor-associated kinase 2 [Source:HGNC Symbol;Acc:6113]	1.34	0.42
ENSG00000197818	SLC9A8	solute carrier family 9. subfamily A (NHE8. cation proton antiporter 8). member 8 [Source:HGNC Symbol;Acc:20728]	1.34	0.42
ENSG00000240040			1.34	0.42

ENSG00000166145	SPINT1	serine peptidase inhibitor. Kunitz type 1 [Source:HGNC Symbol;Acc:11246]	1.34	0.42
ENSG00000188732	FAM221A	family with sequence similarity 221. member A [Source:HGNC Symbol;Acc:27977]	1.34	0.42
ENSG00000188313	PLSCR1	phospholipid scramblase 1 [Source:HGNC Symbol;Acc:9092]	1.33	0.41
ENSG00000125347	IRF1	interferon regulatory factor 1 [Source:HGNC Symbol;Acc:6116]	1.33	0.41
ENSG00000184584	TMEM173	transmembrane protein 173 [Source:HGNC Symbol;Acc:27962]	1.33	0.41
ENSG00000050344	NFE2L3	nuclear factor. erythroid 2-like 3 [Source:HGNC Symbol;Acc:7783]	1.33	0.41
ENSG00000087128	TMPRSS11E	transmembrane protease. serine 11E [Source:HGNC Symbol;Acc:24465]	1.33	0.41
ENSG00000138496	PARP9	poly (ADP-ribose) polymerase family. member 9 [Source:HGNC Symbol;Acc:24118]	1.33	0.41
ENSG00000076685	NT5C2	5'-nucleotidase. cytosolic II [Source:HGNC Symbol;Acc:8022]	1.32	0.4
ENSG00000101040	ZMYND8	zinc finger. MYND-type containing 8 [Source:HGNC Symbol;Acc:9397]	1.32	0.4
ENSG00000140832	MARVELD3	MARVEL domain containing 3 [Source:HGNC Symbol;Acc:30525]	1.32	0.4
ENSG00000138764	CCNG2	cyclin G2 [Source:HGNC Symbol;Acc:1593]	1.32	0.4
ENSG00000106537	TSPAN13	tetraspanin 13 [Source:HGNC Symbol;Acc:21643]	1.32	0.4
ENSG00000008311	AASS	aminoadipate-semialdehyde synthase [Source:HGNC Symbol;Acc:17366]	1.32	0.4
ENSG00000162398	C1orf177	chromosome 1 open reading frame 177 [Source:HGNC Symbol;Acc:26854]	1.32	0.4
ENSG00000158089	GALNT14	UDP-N-acetyl-alpha-D-galactosamine:polypeptide N-acetylgalactosaminyltransferase 14 (GalNAc-T14) [Source:HGNC Symbol;Acc:22946]	1.32	0.4
ENSG00000159399	HK2	hexokinase 2 [Source:HGNC Symbol;Acc:4923]	1.32	0.4
ENSG00000196187	TMEM63A	transmembrane protein 63A [Source:HGNC Symbol;Acc:29118]	1.32	0.4
ENSG00000153094	BCL2L11	BCL2-like 11 (apoptosis facilitator) [Source:HGNC Symbol;Acc:994]	1.31	0.39
ENSG00000140199	SLC12A6	solute carrier family 12 (potassium/chloride transporter). member 6 [Source:HGNC Symbol;Acc:10914]	1.31	0.39
ENSG00000135334	AKIRIN2	akirin 2 [Source:HGNC Symbol;Acc:21407]	1.31	0.39
ENSG00000231925	TAPBP	TAP binding protein (tapasin) [Source:HGNC Symbol;Acc:11566]	1.31	0.39
ENSG00000162298	SYVN1	synovial apoptosis inhibitor 1. synoviolin [Source:HGNC Symbol;Acc:20738]	1.31	0.39
ENSG00000100647	KIAA0247	KIAA0247 [Source:HGNC Symbol;Acc:19956]	1.31	0.39
ENSG00000145103	ILDR1	immunoglobulin-like domain containing receptor 1 [Source:HGNC Symbol;Acc:28741]	1.31	0.38

ENSG00000165795	NDRG2	NDRG family member 2 [Source:HGNC Symbol;Acc:14460]	1.3	0.38
ENSG00000176834	VSIG10	V-set and immunoglobulin domain containing 10 [Source:HGNC Symbol;Acc:26078]	1.3	0.38
ENSG00000184292	TACSTD2	tumor-associated calcium signal transducer 2 [Source:HGNC Symbol;Acc:11530]	1.3	0.38
ENSG00000051596	THOC3	THO complex 3 [Source:HGNC Symbol;Acc:19072]	1.3	0.38
ENSG00000159216	RUNX1	runt-related transcription factor 1 [Source:HGNC Symbol;Acc:10471]	1.3	0.38
ENSG00000189221	MAOA	monoamine oxidase A [Source:HGNC Symbol;Acc:6833]	1.3	0.38
ENSG00000114770	ABCC5	ATP-binding cassette. sub-family C (CFTR/MRP). member 5 [Source:HGNC Symbol;Acc:56]	1.3	0.38
ENSG00000180871	CXCR2	chemokine (C-X-C motif) receptor 2 [Source:HGNC Symbol;Acc:6027]	1.3	0.38
ENSG00000047346	FAM214A	family with sequence similarity 214. member A [Source:HGNC Symbol;Acc:25609]	1.3	0.38
ENSG00000110911	SLC11A2	solute carrier family 11 (proton-coupled divalent metal ion transporter). member 2 [Source:HGNC Symbol;Acc:10908]	1.3	0.38
ENSG00000124782	RREB1	ras responsive element binding protein 1 [Source:HGNC Symbol;Acc:10449]	1.3	0.38
ENSG00000259419	HNRNPCP3	heterogeneous nuclear ribonucleoprotein C pseudogene 3 [Source:HGNC Symbol;Acc:48815]	1.3	0.37
ENSG00000104687	GSR	glutathione reductase [Source:HGNC Symbol;Acc:4623]	1.3	0.37
ENSG00000115112	TFCP2L1	transcription factor CP2-like 1 [Source:HGNC Symbol;Acc:17925]	1.29	0.37
ENSG00000158201	ABHD3	abhydrolase domain containing 3 [Source:HGNC Symbol;Acc:18718]	1.29	0.37
ENSG00000162231	NXF1	nuclear RNA export factor 1 [Source:HGNC Symbol;Acc:8071]	1.29	0.37
ENSG00000163249	CCNYL1	cyclin Y-like 1 [Source:HGNC Symbol;Acc:26868]	1.29	0.37
ENSG00000105281	SLC1A5	solute carrier family 1 (neutral amino acid transporter). member 5 [Source:HGNC Symbol;Acc:10943]	1.29	0.37
ENSG00000134107	BHLHE40	basic helix-loop-helix family. member e40 [Source:HGNC Symbol;Acc:1046]	1.29	0.37
ENSG00000135046	ANXA1	annexin A1 [Source:HGNC Symbol;Acc:533]	1.29	0.37
ENSG00000013016	EHD3	EH-domain containing 3 [Source:HGNC Symbol;Acc:3244]	1.29	0.36
ENSG00000174125	TLR1	toll-like receptor 1 [Source:HGNC Symbol;Acc:11847]	1.29	0.36
ENSG00000173821	RNF213	ring finger protein 213 [Source:HGNC Symbol;Acc:14539]	1.28	0.36
ENSG00000108423	TUBD1	tubulin. delta 1 [Source:HGNC Symbol;Acc:16811]	1.28	0.36
ENSG00000178607	ERN1	endoplasmic reticulum to nucleus signaling 1 [Source:HGNC Symbol;Acc:3449]	1.28	0.36
ENSG00000080823	MOK	MOK protein kinase [Source:HGNC Symbol;Acc:9833]	1.28	0.36

ENSG00000107968	MAP3K8	mitogen-activated protein kinase kinase kinase 8 [Source:HGNC Symbol;Acc:6860]	1.28	0.36
ENSG00000170485	NPAS2	neuronal PAS domain protein 2 [Source:HGNC Symbol;Acc:7895]	1.28	0.36
ENSG00000188811	NHLRC3	NHL repeat containing 3 [Source:HGNC Symbol;Acc:33751]	1.28	0.36
ENSG00000111912	NCOA7	nuclear receptor coactivator 7 [Source:HGNC Symbol;Acc:21081]	1.28	0.36
ENSG00000146411	SLC2A12	solute carrier family 2 (facilitated glucose transporter). member 12 [Source:HGNC Symbol;Acc:18067]	1.28	0.36
ENSG00000149177	PTPRJ	protein tyrosine phosphatase. receptor type. J [Source:HGNC Symbol;Acc:9673]	1.28	0.36
ENSG00000158470	B4GALT5	UDP-Gal:betaGlcNAc beta 1.4- galactosyltransferase. polypeptide 5 [Source:HGNC Symbol;Acc:928]	1.28	0.36
ENSG00000228612	HK2P1	hexokinase 2 pseudogene 1 [Source:HGNC Symbol;Acc:4924]	1.28	0.36
ENSG00000128487	SPECC1	sperm antigen with calponin homology and coiled-coil domains 1 [Source:HGNC Symbol;Acc:30615]	1.28	0.35
ENSG00000154760	SLFN13	schlafen family member 13 [Source:HGNC Symbol;Acc:26481]	1.28	0.35
ENSG00000269374			1.28	0.35
ENSG00000182158	CREB3L2	cAMP responsive element binding protein 3-like 2 [Source:HGNC Symbol;Acc:23720]	1.28	0.35
ENSG00000253677	UBE2HP1	ubiquitin-conjugating enzyme E2H pseudogene 1 [Source:HGNC Symbol;Acc:31079]	1.28	0.35
ENSG00000000003	TSPAN6	tetraspanin 6 [Source:HGNC Symbol;Acc:11858]	1.27	0.35
ENSG00000118515	SGK1	serum/glucocorticoid regulated kinase 1 [Source:HGNC Symbol;Acc:10810]	1.27	0.35
ENSG00000044574	HSPA5	heat shock 70kDa protein 5 (glucose-regulated protein. 78kDa) [Source:HGNC Symbol;Acc:5238]	1.27	0.34
ENSG00000114315	HES1	hes family bHLH transcription factor 1 [Source:HGNC Symbol;Acc:5192]	1.27	0.34
ENSG00000136155	SCEL	sciellin [Source:HGNC Symbol;Acc:10573]	1.27	0.34
ENSG00000156587	UBE2L6	ubiquitin-conjugating enzyme E2L 6 [Source:HGNC Symbol;Acc:12490]	1.27	0.34
ENSG00000172575	RASGRP1	RAS guanyl releasing protein 1 (calcium and DAG-regulated) [Source:HGNC Symbol;Acc:9878]	1.27	0.34
ENSG00000229644	NAMPTL	nicotinamide phosphoribosyltransferase-like [Source:HGNC Symbol;Acc:17633]	1.27	0.34
ENSG00000023909	GCLM	glutamate-cysteine ligase. modifier subunit [Source:HGNC Symbol;Acc:4312]	1.27	0.34
ENSG00000225131	PSME2P2	proteasome activator subunit 2 pseudogene 2 [Source:HGNC Symbol;Acc:30160]	1.27	0.34
ENSG00000157107	FCHO2	FCH domain only 2 [Source:HGNC Symbol;Acc:25180]	1.26	0.34
ENSG00000142867	BCL10	B-cell CLL/lymphoma 10 [Source:HGNC Symbol;Acc:989]	1.26	0.33
ENSG00000120217	CD274	CD274 molecule [Source:HGNC Symbol;Acc:17635]	1.26	0.33

ENSG00000143375	CGN	cingulin [Source:HGNC Symbol;Acc:17429]	1.26	0.33
ENSG00000197442	MAP3K5	mitogen-activated protein kinase kinase kinase 5 [Source:HGNC Symbol;Acc:6857]	1.26	0.33
ENSG00000134909	ARHGAP32	Rho GTPase activating protein 32 [Source:HGNC Symbol;Acc:17399]	1.25	0.33
ENSG00000163611	SPICE1	spindle and centriole associated protein 1 [Source:HGNC Symbol;Acc:25083]	1.25	0.33
ENSG00000104067	TJP1	tight junction protein 1 [Source:HGNC Symbol;Acc:11827]	1.25	0.32
ENSG00000148175	STOM	stomatin [Source:HGNC Symbol;Acc:3383]	1.25	0.32
ENSG00000174640	SLCO2A1	solute carrier organic anion transporter family. member 2A1 [Source:HGNC Symbol;Acc:10955]	1.25	0.32
ENSG00000163399	ATP1A1	ATPase. Na+/K+ transporting. alpha 1 polypeptide [Source:HGNC Symbol;Acc:799]	1.25	0.32
ENSG00000135899	SP110	SP110 nuclear body protein [Source:HGNC Symbol;Acc:5401]	1.25	0.32
ENSG00000171729	TMEM51	transmembrane protein 51 [Source:HGNC Symbol;Acc:25488]	1.25	0.32
ENSG00000185404	SP140L	SP140 nuclear body protein-like [Source:HGNC Symbol;Acc:25105]	1.25	0.32
ENSG00000256737	RBBP4P5	retinoblastoma binding protein 4 pseudogene 5 [Source:HGNC Symbol;Acc:42372]	1.25	0.32
ENSG00000171056	SOX7	SRY (sex determining region Y)-box 7 [Source:HGNC Symbol;Acc:18196]	1.24	0.31
ENSG00000111057	KRT18	keratin 18 [Source:HGNC Symbol;Acc:6430]	1.24	0.31
ENSG00000143036	SLC44A3	solute carrier family 44. member 3 [Source:HGNC Symbol;Acc:28689]	1.24	0.31
ENSG00000250026			1.24	0.31
ENSG00000082438	COBLL1	cordon-bleu WH2 repeat protein-like 1 [Source:HGNC Symbol;Acc:23571]	1.24	0.31
ENSG00000115159	GPD2	glycerol-3-phosphate dehydrogenase 2 (mitochondrial) [Source:HGNC Symbol;Acc:4456]	1.24	0.31
ENSG00000117054	ACADM	acyl-CoA dehydrogenase. C-4 to C-12 straight chain [Source:HGNC Symbol;Acc:89]	1.24	0.31
ENSG00000059378	PARP12	poly (ADP-ribose) polymerase family. member 12 [Source:HGNC Symbol;Acc:21919]	1.24	0.31
ENSG00000125166	GOT2	glutamic-oxaloacetic transaminase 2. mitochondrial [Source:HGNC Symbol;Acc:4433]	1.24	0.31
ENSG00000213839			1.24	0.31
ENSG00000101294	HM13	histocompatibility (minor) 13 [Source:HGNC Symbol;Acc:16435]	1.24	0.31
ENSG00000125733	TRIP10	thyroid hormone receptor interactor 10 [Source:HGNC Symbol;Acc:12304]	1.24	0.31
ENSG00000142583	SLC2A5	solute carrier family 2 (facilitated glucose/fructose transporter). member 5 [Source:HGNC Symbol;Acc:11010]	1.24	0.31
ENSG00000148429	USP6NL	USP6 N-terminal like [Source:HGNC Symbol;Acc:16858]	1.24	0.3

ENSG00000161921	CXCL16	chemokine (C-X-C motif) ligand 16 [Source:HGNC Symbol;Acc:16642]	1.24	0.3
ENSG00000163659	TIPARP	TCDD-inducible poly(ADP-ribose) polymerase [Source:HGNC Symbol;Acc:23696]	1.24	0.3
ENSG00000205413	SAMD9	sterile alpha motif domain containing 9 [Source:HGNC Symbol;Acc:1348]	1.24	0.3
ENSG00000198380	GFPT1	glutamine--fructose-6-phosphate transaminase 1 [Source:HGNC Symbol;Acc:4241]	1.23	0.3
ENSG00000198399	ITSN2	intersectin 2 [Source:HGNC Symbol;Acc:6184]	1.23	0.3
ENSG00000143801	PSEN2	presenilin 2 (Alzheimer disease 4) [Source:HGNC Symbol;Acc:9509]	1.23	0.3
ENSG00000168564	CDKN2AIP	CDKN2A interacting protein [Source:HGNC Symbol;Acc:24325]	1.23	0.3
ENSG00000166689	PLEKHA7	pleckstrin homology domain containing. family A member 7 [Source:HGNC Symbol;Acc:27049]	1.23	0.3
ENSG00000023330	ALAS1	aminolevulinate. delta-. synthase 1 [Source:HGNC Symbol;Acc:396]	1.23	0.3
ENSG00000162415	ZSWIM5	zinc finger. SWIM-type containing 5 [Source:HGNC Symbol;Acc:29299]	1.23	0.3
ENSG00000162836	ACP6	acid phosphatase 6. lysophosphatidic [Source:HGNC Symbol;Acc:29609]	1.23	0.3
ENSG00000163932	PRKCD	protein kinase C. delta [Source:HGNC Symbol;Acc:9399]	1.23	0.3
ENSG00000223572	CKMT1A	creatine kinase. mitochondrial 1A [Source:HGNC Symbol;Acc:31736]	1.23	0.3
ENSG00000112294	ALDH5A1	aldehyde dehydrogenase 5 family. member A1 [Source:HGNC Symbol;Acc:408]	1.23	0.29
ENSG00000189067	LITAF	lipopolysaccharide-induced TNF factor [Source:HGNC Symbol;Acc:16841]	1.23	0.29
ENSG00000146904	EPHA1	EPH receptor A1 [Source:HGNC Symbol;Acc:3385]	1.23	0.29
ENSG00000176986	SEC24C	SEC24 family member C [Source:HGNC Symbol;Acc:10705]	1.22	0.29
ENSG00000213523	SRA1	steroid receptor RNA activator 1 [Source:HGNC Symbol;Acc:11281]	1.22	0.29
ENSG00000152229	PSTPIP2	proline-serine-threonine phosphatase interacting protein 2 [Source:HGNC Symbol;Acc:9581]	1.22	0.29
ENSG00000101310	SEC23B	Sec23 homolog B (S. cerevisiae) [Source:HGNC Symbol;Acc:10702]	1.22	0.29
ENSG00000259838	TCEB1P2	transcription elongation factor B (SIII). polypeptide 1 pseudogene 2 [Source:HGNC Symbol;Acc:17762]	1.22	0.29
ENSG00000189376	C8orf76	chromosome 8 open reading frame 76 [Source:HGNC Symbol;Acc:25924]	1.22	0.29
ENSG00000132256	TRIM5	tripartite motif containing 5 [Source:HGNC Symbol;Acc:16276]	1.22	0.28
ENSG00000142197	DOPEY2	dopey family member 2 [Source:HGNC Symbol;Acc:1291]	1.22	0.28
ENSG00000187054	TMPRSS11A	transmembrane protease. serine 11A [Source:HGNC Symbol;Acc:27954]	1.22	0.28
ENSG00000136379	ABHD17C	abhydrolase domain containing 17C [Source:HGNC Symbol;Acc:26925]	1.22	0.28

ENSG00000100911	PSME2	proteasome (prosome. macropain) activator subunit 2 (PA28 beta) [Source:HGNC Symbol;Acc:9569]	1.21	0.28
ENSG00000111335	OAS2	2'-5'-oligoadenylate synthetase 2. 69/71kDa [Source:HGNC Symbol;Acc:8087]	1.21	0.28
ENSG00000161533	ACOX1	acyl-CoA oxidase 1. palmitoyl [Source:HGNC Symbol;Acc:119]	1.21	0.28
ENSG00000108669	CYTH1	cytohesin 1 [Source:HGNC Symbol;Acc:9501]	1.21	0.28
ENSG00000153391	INO80C	INO80 complex subunit C [Source:HGNC Symbol;Acc:26994]	1.21	0.28
ENSG00000120519	SLC10A7	solute carrier family 10. member 7 [Source:HGNC Symbol;Acc:23088]	1.21	0.28
ENSG00000126247	CAPNS1	calpain. small subunit 1 [Source:HGNC Symbol;Acc:1481]	1.21	0.28
ENSG00000130449	ZSWIM6	zinc finger. SWIM-type containing 6 [Source:HGNC Symbol;Acc:29316]	1.21	0.28
ENSG00000254013	MAP2K1P1	mitogen-activated protein kinase kinase 1 pseudogene 1 [Source:HGNC Symbol;Acc:6841]	1.21	0.28
ENSG00000141279	NPEPPS	aminopeptidase puromycin sensitive [Source:HGNC Symbol;Acc:7900]	1.21	0.28
ENSG00000105835	NAMPT	nicotinamide phosphoribosyltransferase [Source:HGNC Symbol;Acc:30092]	1.21	0.27
ENSG00000107862	GBF1	golgi brefeldin A resistant guanine nucleotide exchange factor 1 [Source:HGNC Symbol;Acc:4181]	1.21	0.27
ENSG00000110344	UBE4A	ubiquitination factor E4A [Source:HGNC Symbol;Acc:12499]	1.21	0.27
ENSG00000170545	SMAGP	small cell adhesion glycoprotein [Source:HGNC Symbol;Acc:26918]	1.21	0.27
ENSG00000179218	CALR	calreticulin [Source:HGNC Symbol;Acc:1455]	1.21	0.27
ENSG00000111684	LPCAT3	lysophosphatidylcholine acyltransferase 3 [Source:HGNC Symbol;Acc:30244]	1.21	0.27
ENSG00000114541	FRMD4B	FERM domain containing 4B [Source:HGNC Symbol;Acc:24886]	1.2	0.27
ENSG00000137767	SQRDL	sulfide quinone reductase-like (yeast) [Source:HGNC Symbol;Acc:20390]	1.2	0.27
ENSG00000164327	RICTOR	RPTOR independent companion of MTOR. complex 2 [Source:HGNC Symbol;Acc:28611]	1.2	0.27
ENSG00000086062	B4GALT1	UDP-Gal:betaGlcNAc beta 1.4- galactosyltransferase. polypeptide 1 [Source:HGNC Symbol;Acc:924]	1.2	0.27
ENSG00000110583	NAA40	N(alpha)-acetyltransferase 40. NatD catalytic subunit [Source:HGNC Symbol;Acc:25845]	1.2	0.27
ENSG00000163814	CDCP1	CUB domain containing protein 1 [Source:HGNC Symbol;Acc:24357]	1.2	0.27
ENSG00000204267	TAP2	transporter 2. ATP-binding cassette. sub-family B (MDR/TAP) [Source:HGNC Symbol;Acc:44]	1.2	0.27
ENSG00000224186	C5orf66	chromosome 5 open reading frame 66 [Source:HGNC Symbol;Acc:48332]	1.2	0.26
ENSG00000076108	BAZ2A	bromodomain adjacent to zinc finger domain. 2A [Source:HGNC Symbol;Acc:962]	1.2	0.26
ENSG00000122779	TRIM24	tripartite motif containing 24 [Source:HGNC Symbol;Acc:11812]	1.2	0.26

ENSG00000183337	BCOR	BCL6 corepressor [Source:HGNC Symbol;Acc:20893]	1.2	0.26
ENSG00000072201	LNX1	ligand of numb-protein X 1. E3 ubiquitin protein ligase [Source:HGNC Symbol;Acc:6657]	1.2	0.26
ENSG00000113615	SEC24A	SEC24 family member A [Source:HGNC Symbol;Acc:10703]	1.2	0.26
ENSG00000162664	ZNF326	zinc finger protein 326 [Source:HGNC Symbol;Acc:14104]	1.2	0.26
ENSG00000168610	STAT3	signal transducer and activator of transcription 3 (acute-phase response factor) [Source:HGNC Symbol;Acc:11364]	1.2	0.26
ENSG00000178974	FBXO34	F-box protein 34 [Source:HGNC Symbol;Acc:20201]	1.2	0.26
ENSG00000171940	ZNF217	zinc finger protein 217 [Source:HGNC Symbol;Acc:13009]	1.19	0.25
ENSG00000175806	MSRA	methionine sulfoxide reductase A [Source:HGNC Symbol;Acc:7377]	1.19	0.25
ENSG00000170340	B3GNT2	UDP-GlcNAc:betaGal beta-1.3-N-acetylglucosaminyltransferase 2 [Source:HGNC Symbol;Acc:15629]	1.19	0.25
ENSG00000113282	CLINT1	clathrin interactor 1 [Source:HGNC Symbol;Acc:23186]	1.19	0.25
ENSG00000163840	DTX3L	deltex 3-like (Drosophila) [Source:HGNC Symbol;Acc:30323]	1.19	0.25
ENSG00000125107	CNOT1	CCR4-NOT transcription complex. subunit 1 [Source:HGNC Symbol;Acc:7877]	1.19	0.25
ENSG00000133985	TTC9	tetratricopeptide repeat domain 9 [Source:HGNC Symbol;Acc:20267]	1.19	0.25
ENSG00000112079	STK38	serine/threonine kinase 38 [Source:HGNC Symbol;Acc:17847]	1.19	0.25
ENSG00000140396	NCOA2	nuclear receptor coactivator 2 [Source:HGNC Symbol;Acc:7669]	1.19	0.25
ENSG00000153066	TXNDC11	thioredoxin domain containing 11 [Source:HGNC Symbol;Acc:28030]	1.19	0.25
ENSG00000182179	UBA7	ubiquitin-like modifier activating enzyme 7 [Source:HGNC Symbol;Acc:12471]	1.19	0.25
ENSG00000136653	RASSF5	Ras association (RalGDS/AF-6) domain family member 5 [Source:HGNC Symbol;Acc:17609]	1.19	0.25
ENSG00000084676	NCOA1	nuclear receptor coactivator 1 [Source:HGNC Symbol;Acc:7668]	1.19	0.25
ENSG00000124151	NCOA3	nuclear receptor coactivator 3 [Source:HGNC Symbol;Acc:7670]	1.19	0.25
ENSG00000134256	CD101	CD101 molecule [Source:HGNC Symbol;Acc:5949]	1.19	0.25
ENSG00000078747	ITCH	itchy E3 ubiquitin protein ligase [Source:HGNC Symbol;Acc:13890]	1.19	0.24
ENSG00000156804	FBXO32	F-box protein 32 [Source:HGNC Symbol;Acc:16731]	1.18	0.24
ENSG00000157184	CPT2	carnitine palmitoyltransferase 2 [Source:HGNC Symbol;Acc:2330]	1.18	0.24
ENSG00000065357	DGKA	diacylglycerol kinase. alpha 80kDa [Source:HGNC Symbol;Acc:2849]	1.18	0.24

ENSG00000070081	NUCB2	nucleobindin 2 [Source:HGNC Symbol;Acc:8044]	1.18	0.24
ENSG00000175054	ATR	ataxia telangiectasia and Rad3 related [Source:HGNC Symbol;Acc:882]	1.18	0.24
ENSG00000182934	SRPR	signal recognition particle receptor (docking protein) [Source:HGNC Symbol;Acc:11307]	1.18	0.24
ENSG00000082146	STRADB	STE20-related kinase adaptor beta [Source:HGNC Symbol;Acc:13205]	1.18	0.24
ENSG00000141452	C18orf8	chromosome 18 open reading frame 8 [Source:HGNC Symbol;Acc:24326]	1.18	0.24
ENSG00000151689	INPP1	inositol polyphosphate-1-phosphatase [Source:HGNC Symbol;Acc:6071]	1.18	0.24
ENSG00000153214	TMEM87B	transmembrane protein 87B [Source:HGNC Symbol;Acc:25913]	1.18	0.24
ENSG00000189091	SF3B3	splicing factor 3b. subunit 3. 130kDa [Source:HGNC Symbol;Acc:10770]	1.18	0.24
ENSG00000181192	DHTKD1	dehydrogenase E1 and transketolase domain containing 1 [Source:HGNC Symbol;Acc:23537]	1.18	0.24
ENSG00000052841	TTC17	tetratricopeptide repeat domain 17 [Source:HGNC Symbol;Acc:25596]	1.18	0.24
ENSG00000135373	EHF	ets homologous factor [Source:HGNC Symbol;Acc:3246]	1.18	0.24
ENSG00000132906	CASP9	caspase 9. apoptosis-related cysteine peptidase [Source:HGNC Symbol;Acc:1511]	1.18	0.23
ENSG00000167565	SERTAD3	SERTA domain containing 3 [Source:HGNC Symbol;Acc:17931]	1.18	0.23
ENSG00000088970	PLK1S1	polo-like kinase 1 substrate 1 [Source:HGNC Symbol;Acc:15865]	1.18	0.23
ENSG00000146409	SLC18B1	solute carrier family 18. subfamily B. member 1 [Source:HGNC Symbol;Acc:21573]	1.18	0.23
ENSG00000149428	HYOU1	hypoxia up-regulated 1 [Source:HGNC Symbol;Acc:16931]	1.17	0.23
ENSG00000105127	AKAP8	A kinase (PRKA) anchor protein 8 [Source:HGNC Symbol;Acc:378]	1.17	0.23
ENSG00000155287	SLC25A28	solute carrier family 25 (mitochondrial iron transporter). member 28 [Source:HGNC Symbol;Acc:23472]	1.17	0.23
ENSG00000169032	MAP2K1	mitogen-activated protein kinase kinase 1 [Source:HGNC Symbol;Acc:6840]	1.17	0.23
ENSG00000184831	APOO	apolipoprotein O [Source:HGNC Symbol;Acc:28727]	1.17	0.23
ENSG00000102699	PARP4	poly (ADP-ribose) polymerase family. member 4 [Source:HGNC Symbol;Acc:271]	1.17	0.23
ENSG00000166348	USP54	ubiquitin specific peptidase 54 [Source:HGNC Symbol;Acc:23513]	1.17	0.23
ENSG00000132694	ARHGEF11	Rho guanine nucleotide exchange factor (GEF) 11 [Source:HGNC Symbol;Acc:14580]	1.17	0.23
ENSG00000143374	TARS2	threonyl-tRNA synthetase 2. mitochondrial (putative) [Source:HGNC Symbol;Acc:30740]	1.17	0.23
ENSG00000196218	RYR1	ryanodine receptor 1 (skeletal) [Source:HGNC Symbol;Acc:10483]	1.17	0.23
ENSG00000204178	TMEM57	transmembrane protein 57 [Source:HGNC Symbol;Acc:25572]	1.17	0.23

ENSG00000102763	VWA8	von Willebrand factor A domain containing 8 [Source:HGNC Symbol;Acc:29071]	1.17	0.22
ENSG00000162645	GBP2	guanylate binding protein 2. interferon-inducible [Source:HGNC Symbol;Acc:4183]	1.17	0.22
ENSG00000023318	ERP44	endoplasmic reticulum protein 44 [Source:HGNC Symbol;Acc:18311]	1.17	0.22
ENSG00000111144	LTA4H	leukotriene A4 hydrolase [Source:HGNC Symbol;Acc:6710]	1.17	0.22
ENSG00000165219	GAPVD1	GTPase activating protein and VPS9 domains 1 [Source:HGNC Symbol;Acc:23375]	1.17	0.22
ENSG00000165929	TC2N	tandem C2 domains. nuclear [Source:HGNC Symbol;Acc:19859]	1.17	0.22
ENSG00000065665	SEC61A2	Sec61 alpha 2 subunit (S. cerevisiae) [Source:HGNC Symbol;Acc:17702]	1.17	0.22
ENSG00000197555	SIPA1L1	signal-induced proliferation-associated 1 like 1 [Source:HGNC Symbol;Acc:20284]	1.17	0.22
ENSG00000111725	PRKAB1	protein kinase. AMP-activated. beta 1 non-catalytic subunit [Source:HGNC Symbol;Acc:9378]	1.17	0.22
ENSG00000134910	STT3A	STT3A. subunit of the oligosaccharyltransferase complex (catalytic) [Source:HGNC Symbol;Acc:6172]	1.16	0.22
ENSG00000178741	COX5A	cytochrome c oxidase subunit Va [Source:HGNC Symbol;Acc:2267]	1.16	0.22
ENSG00000163694	RBM47	RNA binding motif protein 47 [Source:HGNC Symbol;Acc:30358]	1.16	0.22
ENSG00000225190	PLEKHM1	pleckstrin homology domain containing. family M (with RUN domain) member 1 [Source:HGNC Symbol;Acc:29017]	1.16	0.22
ENSG00000129595	EPB41L4A	erythrocyte membrane protein band 4.1 like 4A [Source:HGNC Symbol;Acc:13278]	1.16	0.21
ENSG00000092010	PSME1	proteasome (prosome. macropain) activator subunit 1 (PA28 alpha) [Source:HGNC Symbol;Acc:9568]	1.16	0.21
ENSG00000198168	SVIP	small VCP/p97-interacting protein [Source:HGNC Symbol;Acc:25238]	1.16	0.21
ENSG00000115241	PPM1G	protein phosphatase. Mg2+/Mn2+ dependent. 1G [Source:HGNC Symbol;Acc:9278]	1.16	0.21
ENSG00000102554	KLF5	Kruppel-like factor 5 (intestinal) [Source:HGNC Symbol;Acc:6349]	1.16	0.21
ENSG00000121073	SLC35B1	solute carrier family 35. member B1 [Source:HGNC Symbol;Acc:20798]	1.16	0.21
ENSG00000168538	TRAPPC11	trafficking protein particle complex 11 [Source:HGNC Symbol;Acc:25751]	1.16	0.21
ENSG00000230673	PABPC1P3	poly(A) binding protein. cytoplasmic 1 pseudogene 3 [Source:HGNC Symbol;Acc:8560]	1.16	0.21
ENSG00000204564	C6orf136	chromosome 6 open reading frame 136 [Source:HGNC Symbol;Acc:21301]	1.15	0.21
ENSG00000050327	ARHGEF5	Rho guanine nucleotide exchange factor (GEF) 5 [Source:HGNC Symbol;Acc:13209]	1.15	0.2
ENSG00000103512	NOMO1	NODAL modulator 1 [Source:HGNC Symbol;Acc:30060]	1.15	0.2
ENSG00000168310	IRF2	interferon regulatory factor 2 [Source:HGNC Symbol;Acc:6117]	1.15	0.2

ENSG00000062485	CS	citrate synthase [Source:HGNC Symbol;Acc:2422]	1.15	0.2
ENSG00000198561	CTNND1	catenin (cadherin-associated protein). delta 1 [Source:HGNC Symbol;Acc:2515]	1.15	0.2
ENSG00000215271	HOMEZ	homeobox and leucine zipper encoding [Source:HGNC Symbol;Acc:20164]	1.15	0.2
ENSG00000113916	BCL6	B-cell CLL/lymphoma 6 [Source:HGNC Symbol;Acc:1001]	1.15	0.2
ENSG00000130254	SAFB2	scaffold attachment factor B2 [Source:HGNC Symbol;Acc:21605]	1.15	0.2
ENSG00000139083	ETV6	ets variant 6 [Source:HGNC Symbol;Acc:3495]	1.15	0.2
ENSG00000003147	ICA1	islet cell autoantigen 1. 69kDa [Source:HGNC Symbol;Acc:5343]	1.15	0.2
ENSG00000044115	CTNNA1	catenin (cadherin-associated protein). alpha 1. 102kDa [Source:HGNC Symbol;Acc:2509]	1.15	0.2
ENSG00000073969	NSF	N-ethylmaleimide-sensitive factor [Source:HGNC Symbol;Acc:8016]	1.15	0.2
ENSG00000163565	IFI16	interferon. gamma-inducible protein 16 [Source:HGNC Symbol;Acc:5395]	1.15	0.2
ENSG00000213593	TMX2	thioredoxin-related transmembrane protein 2 [Source:HGNC Symbol;Acc:30739]	1.14	0.19
ENSG00000065060	UHRF1BP1	UHRF1 binding protein 1 [Source:HGNC Symbol;Acc:21216]	1.14	0.19
ENSG00000158435	CNOT11	CCR4-NOT transcription complex. subunit 11 [Source:HGNC Symbol;Acc:25217]	1.14	0.19
ENSG00000181789	COPG1	coatomer protein complex. subunit gamma 1 [Source:HGNC Symbol;Acc:2236]	1.14	0.19
ENSG00000071205	ARHGAP10	Rho GTPase activating protein 10 [Source:HGNC Symbol;Acc:26099]	1.14	0.19
ENSG00000116478	HDAC1	histone deacetylase 1 [Source:HGNC Symbol;Acc:4852]	1.14	0.19
ENSG00000162909	CAPN2	calpain 2. (m/II) large subunit [Source:HGNC Symbol;Acc:1479]	1.14	0.19
ENSG00000175198	PCCA	propionyl CoA carboxylase. alpha polypeptide [Source:HGNC Symbol;Acc:8653]	1.14	0.19
ENSG00000120053	GOT1	glutamic-oxaloacetic transaminase 1. soluble [Source:HGNC Symbol;Acc:4432]	1.14	0.19
ENSG00000155463	OXA1L	oxidase (cytochrome c) assembly 1-like [Source:HGNC Symbol;Acc:8526]	1.14	0.19
ENSG00000160218	TRAPPC10	trafficking protein particle complex 10 [Source:HGNC Symbol;Acc:11868]	1.14	0.19
ENSG00000148498	PARD3	par-3 family cell polarity regulator [Source:HGNC Symbol;Acc:16051]	1.14	0.19
ENSG00000133835	HSD17B4	hydroxysteroid (17-beta) dehydrogenase 4 [Source:HGNC Symbol;Acc:5213]	1.14	0.19
ENSG00000162736	NCSTN	nicastrin [Source:HGNC Symbol;Acc:17091]	1.14	0.19
ENSG00000118705	RPN2	ribophorin II [Source:HGNC Symbol;Acc:10382]	1.14	0.18
ENSG00000133731	IMPA1	inositol(myo)-1(or 4)-monophosphatase 1 [Source:HGNC Symbol;Acc:6050]	1.14	0.18

ENSG00000143384	MCL1	myeloid cell leukemia sequence 1 (BCL2-related) [Source:HGNC Symbol;Acc:6943]	1.14	0.18
ENSG00000021776	AQR	aquarius intron-binding spliceosomal factor [Source:HGNC Symbol;Acc:29513]	1.14	0.18
ENSG00000102572	STK24	serine/threonine kinase 24 [Source:HGNC Symbol;Acc:11403]	1.14	0.18
ENSG00000103381	CPPED1	calcineurin-like phosphoesterase domain containing 1 [Source:HGNC Symbol;Acc:25632]	1.14	0.18
ENSG00000243646	IL10RB	interleukin 10 receptor. beta [Source:HGNC Symbol;Acc:5965]	1.14	0.18
ENSG00000079739	PGM1	phosphoglucomutase 1 [Source:HGNC Symbol;Acc:8905]	1.13	0.18
ENSG00000119203	CPSF3	cleavage and polyadenylation specific factor 3. 73kDa [Source:HGNC Symbol;Acc:2326]	1.13	0.18
ENSG00000164466	SFXN1	sideroflexin 1 [Source:HGNC Symbol;Acc:16085]	1.13	0.18
ENSG00000167004	PDIA3	protein disulfide isomerase family A. member 3 [Source:HGNC Symbol;Acc:4606]	1.13	0.18
ENSG00000115109	EPB41L5	erythrocyte membrane protein band 4.1 like 5 [Source:HGNC Symbol;Acc:19819]	1.13	0.18
ENSG00000180867	PDIA3P1	protein disulfide isomerase family A. member 3 pseudogene 1 [Source:HGNC Symbol;Acc:4607]	1.13	0.18
ENSG00000185650	ZFP36L1	ZFP36 ring finger protein-like 1 [Source:HGNC Symbol;Acc:1107]	1.13	0.18
ENSG00000205352	PRR13	proline rich 13 [Source:HGNC Symbol;Acc:24528]	1.13	0.18
ENSG00000131844	MCCC2	methylcrotonoyl-CoA carboxylase 2 (beta) [Source:HGNC Symbol;Acc:6937]	1.13	0.18
ENSG00000267809	NDUFV2P1	NADH dehydrogenase (ubiquinone) flavoprotein 2 pseudogene 1 [Source:HGNC Symbol;Acc:7718]	1.13	0.18
ENSG00000090061	CCNK	cyclin K [Source:HGNC Symbol;Acc:1596]	1.13	0.17
ENSG00000163755	HPS3	Hermansky-Pudlak syndrome 3 [Source:HGNC Symbol;Acc:15597]	1.13	0.17
ENSG00000168827	GFM1	G elongation factor. mitochondrial 1 [Source:HGNC Symbol;Acc:13780]	1.13	0.17
ENSG00000150768	DLAT	dihydrolipoamide S-acetyltransferase [Source:HGNC Symbol;Acc:2896]	1.13	0.17
ENSG00000076513	ANKRD13A	ankyrin repeat domain 13A [Source:HGNC Symbol;Acc:21268]	1.13	0.17
ENSG00000123737	EXOSC9	exosome component 9 [Source:HGNC Symbol;Acc:9137]	1.13	0.17
ENSG00000091157	WDR7	WD repeat domain 7 [Source:HGNC Symbol;Acc:13490]	1.12	0.17
ENSG00000084463	WBP11	WW domain binding protein 11 [Source:HGNC Symbol;Acc:16461]	1.12	0.16
ENSG00000131725	WDR44	WD repeat domain 44 [Source:HGNC Symbol;Acc:30512]	1.12	0.16
ENSG00000268032			1.12	0.16
ENSG00000069974	RAB27A	RAB27A. member RAS oncogene family [Source:HGNC Symbol;Acc:9766]	1.12	0.16

ENSG00000184432	COPB2	coatomer protein complex. subunit beta 2 (beta prime) [Source:HGNC Symbol;Acc:2232]	1.12	0.16
ENSG00000107959	PITRM1	pitrilysin metallopeptidase 1 [Source:HGNC Symbol;Acc:17663]	1.12	0.16
ENSG00000155561	NUP205	nucleoporin 205kDa [Source:HGNC Symbol;Acc:18658]	1.12	0.16
ENSG00000006453	BAIAP2L1	BAI1-associated protein 2-like 1 [Source:HGNC Symbol;Acc:21649]	1.12	0.16
ENSG00000136636	KCTD3	potassium channel tetramerization domain containing 3 [Source:HGNC Symbol;Acc:21305]	1.12	0.16
ENSG00000101811	CSTF2	cleavage stimulation factor. 3' pre-RNA. subunit 2. 64kDa [Source:HGNC Symbol;Acc:2484]	1.12	0.16
ENSG00000166889	PATL1	protein associated with topoisomerase II homolog 1 (yeast) [Source:HGNC Symbol;Acc:26721]	1.12	0.16
ENSG00000174437	ATP2A2	ATPase. Ca++ transporting. cardiac muscle. slow twitch 2 [Source:HGNC Symbol;Acc:812]	1.11	0.16
ENSG00000144468	RHBDD1	rhomboid domain containing 1 [Source:HGNC Symbol;Acc:23081]	1.11	0.15
ENSG00000156504	FAM122B	family with sequence similarity 122B [Source:HGNC Symbol;Acc:30490]	1.11	0.15
ENSG00000165209	STRBP	spermatid perinuclear RNA binding protein [Source:HGNC Symbol;Acc:16462]	1.11	0.15
ENSG00000010244	ZNF207	zinc finger protein 207 [Source:HGNC Symbol;Acc:12998]	1.11	0.15
ENSG00000114354	TFG	TRK-fused gene [Source:HGNC Symbol;Acc:11758]	1.11	0.15
ENSG00000143106	PSMA5	proteasome (prosome. macropain) subunit. alpha type. 5 [Source:HGNC Symbol;Acc:9534]	1.11	0.15
ENSG00000170322	NFRKB	nuclear factor related to kappaB binding protein [Source:HGNC Symbol;Acc:7802]	1.11	0.15
ENSG00000198836	OPA1	optic atrophy 1 (autosomal dominant) [Source:HGNC Symbol;Acc:8140]	1.11	0.15
ENSG00000121390	PSPC1	paraspeckle component 1 [Source:HGNC Symbol;Acc:20320]	1.11	0.15
ENSG00000030419	IKZF2	IKAROS family zinc finger 2 (Helios) [Source:HGNC Symbol;Acc:13177]	1.11	0.15
ENSG00000166747	AP1G1	adaptor-related protein complex 1. gamma 1 subunit [Source:HGNC Symbol;Acc:555]	1.11	0.15
ENSG00000120802	TMPO	thymopoietin [Source:HGNC Symbol;Acc:11875]	1.1	0.14
ENSG00000135090	TAOK3	TAO kinase 3 [Source:HGNC Symbol;Acc:18133]	1.1	0.14
ENSG00000172269	DPAGT1	dolichyl-phosphate (UDP-N-acetylglucosamine) N-acetylglucosaminephosphotransferase 1 (GlcNAc-1-P transferase) [Source:HGNC Symbol;Acc:2995]	1.1	0.14
ENSG00000058799	YIPF1	Yip1 domain family. member 1 [Source:HGNC Symbol;Acc:25231]	1.1	0.14
ENSG00000214013	GANC	glucosidase. alpha; neutral C [Source:HGNC Symbol;Acc:4139]	1.1	0.14
ENSG00000108039	XPNPEP1	X-prolyl aminopeptidase (aminopeptidase P) 1. soluble [Source:HGNC Symbol;Acc:12822]	1.1	0.13
ENSG00000117758	STX12	syntaxin 12 [Source:HGNC Symbol;Acc:11430]	1.1	0.13

ENSG00000166908	PIP4K2C	phosphatidylinositol-5-phosphate 4-kinase. type II. gamma [Source:HGNC Symbol;Acc:23786]	1.1	0.13
ENSG00000231043			1.1	0.13
ENSG00000067596	DHX8	DEAH (Asp-Glu-Ala-His) box polypeptide 8 [Source:HGNC Symbol;Acc:2749]	1.09	0.13
ENSG00000100325	ASCC2	activating signal cointegrator 1 complex subunit 2 [Source:HGNC Symbol;Acc:24103]	1.09	0.13
ENSG00000156709	AIFM1	apoptosis-inducing factor. mitochondrion-associated. 1 [Source:HGNC Symbol;Acc:8768]	1.09	0.13
ENSG00000173812	EIF1	eukaryotic translation initiation factor 1 [Source:HGNC Symbol;Acc:3249]	1.09	0.13
ENSG00000168710	AHCYL1	adenosylhomocysteinase-like 1 [Source:HGNC Symbol;Acc:344]	1.09	0.12
ENSG00000164180	TMEM161B	transmembrane protein 161B [Source:HGNC Symbol;Acc:28483]	1.09	0.12
ENSG00000110075	PPP6R3	protein phosphatase 6. regulatory subunit 3 [Source:HGNC Symbol;Acc:1173]	1.09	0.12
ENSG00000110435	PDHX	pyruvate dehydrogenase complex. component X [Source:HGNC Symbol;Acc:21350]	1.08	0.11
ENSG00000131504	DIAPH1	diaphanous-related formin 1 [Source:HGNC Symbol;Acc:2876]	1.08	0.11
ENSG00000149532	CPSF7	cleavage and polyadenylation specific factor 7. 59kDa [Source:HGNC Symbol;Acc:30098]	1.08	0.11
ENSG00000159256	MORC3	MORC family CW-type zinc finger 3 [Source:HGNC Symbol;Acc:23572]	1.08	0.11
ENSG00000149480	MTA2	metastasis associated 1 family. member 2 [Source:HGNC Symbol;Acc:7411]	1.08	0.11
ENSG00000114745	GORASP1	golgi reassembly stacking protein 1. 65kDa [Source:HGNC Symbol;Acc:16769]	1.08	0.11
ENSG00000137947	GTF2B	general transcription factor IIB [Source:HGNC Symbol;Acc:4648]	1.07	0.1
ENSG00000174243	DDX23	DEAD (Asp-Glu-Ala-Asp) box polypeptide 23 [Source:HGNC Symbol;Acc:17347]	1.07	0.1
ENSG00000131269	ABCB7	ATP-binding cassette. sub-family B (MDR/TAP). member 7 [Source:HGNC Symbol;Acc:48]	1.07	0.1
ENSG00000115806	GORASP2	golgi reassembly stacking protein 2. 55kDa [Source:HGNC Symbol;Acc:17500]	1.07	0.1
ENSG00000133961	NUMB	numb homolog (Drosophila) [Source:HGNC Symbol;Acc:8060]	1.07	0.1
ENSG00000006530	AGK	acylglycerol kinase [Source:HGNC Symbol;Acc:21869]	1.07	0.09
ENSG00000138101	DTNB	dystrobrevin. beta [Source:HGNC Symbol;Acc:3058]	1.07	0.09
ENSG00000176953	NFATC2IP	nuclear factor of activated T-cells. cytoplasmic. calcineurin-dependent 2 interacting protein [Source:HGNC Symbol;Acc:25906]	1.05	0.07

Table S2b. Down-regulated genes in periodontitis.

Ensembl ID	Gene symbol	Gene product	Fold change	$\log_2$ fold change
ENSG00000139648	KRT71	keratin 71 [Source:HGNC Symbol;Acc:28927]	< -10	> 10
ENSG00000170374	SP7	Sp7 transcription factor [Source:HGNC Symbol;Acc:17321]	< -10	> 10
ENSG00000131668	BARX1	BARX homeobox 1 [Source:HGNC Symbol;Acc:955]	-4.52	2.18
ENSG00000105664	COMP	cartilage oligomeric matrix protein [Source:HGNC Symbol;Acc:2227]	-4.29	2.1
ENSG00000104435	STMN2	stathmin-like 2 [Source:HGNC Symbol;Acc:10577]	-3.62	1.86
ENSG00000269729			-3.57	1.84
ENSG00000231605			-3.51	1.81
ENSG00000178172	SPINK6	serine peptidase inhibitor, Kazal type 6 [Source:HGNC Symbol;Acc:29486]	-3.45	1.79
ENSG00000146250	PRSS35	protease, serine, 35 [Source:HGNC Symbol;Acc:21387]	-3.4	1.77
ENSG00000130600	H19	H19, imprinted maternally expressed transcript (non-protein coding) [Source:HGNC Symbol;Acc:4713]	-3.27	1.71
ENSG00000108821	COL1A1	collagen, type I, alpha 1 [Source:HGNC Symbol;Acc:2197]	-3.13	1.65
ENSG00000144891	AGTR1	angiotensin II receptor, type 1 [Source:HGNC Symbol;Acc:336]	-3.11	1.63
ENSG00000064205	WISP2	WNT1 inducible signaling pathway protein 2 [Source:HGNC Symbol;Acc:12770]	-3.01	1.59
ENSG00000106819	ASPN	asporin [Source:HGNC Symbol;Acc:14872]	-2.94	1.56
ENSG00000123500	COL10A1	collagen, type X, alpha 1 [Source:HGNC Symbol;Acc:2185]	-2.93	1.55
ENSG00000140873	ADAMTS18	ADAM metallopeptidase with thrombospondin type 1 motif, 18 [Source:HGNC Symbol;Acc:17110]	-2.89	1.53
ENSG00000127083	OMD	osteomodulin [Source:HGNC Symbol;Acc:8134]	-2.86	1.51
ENSG00000112319	EYA4	eyes absent homolog 4 (Drosophila) [Source:HGNC Symbol;Acc:3522]	-2.84	1.51
ENSG00000173641	HSPB7	heat shock 27kDa protein family, member 7 (cardiovascular) [Source:HGNC Symbol;Acc:5249]	-2.83	1.5
ENSG00000224127			-2.83	1.5
ENSG00000104213	PDGFRL	platelet-derived growth factor receptor-like [Source:HGNC Symbol;Acc:8805]	-2.81	1.49
ENSG00000261213			-2.81	1.49
ENSG00000106809	OGN	osteoglycin [Source:HGNC Symbol;Acc:8126]	-2.79	1.48

ENSG00000174473	GALNTL6	UDP-N-acetyl-alpha-D-galactosamine:polypeptide N-acetylgalactosaminyltransferase-like 6 [Source:HGNC Symbol;Acc:33844]	-2.78	1.47
ENSG00000242265	PEG10	paternally expressed 10 [Source:HGNC Symbol;Acc:14005]	-2.75	1.46
ENSG00000157766	ACAN	aggrecan [Source:HGNC Symbol;Acc:319]	-2.73	1.45
ENSG00000006283	CACNA1G	calcium channel, voltage-dependent, T type, alpha 1G subunit [Source:HGNC Symbol;Acc:1394]	-2.72	1.45
ENSG00000060718	COL11A1	collagen, type XI, alpha 1 [Source:HGNC Symbol;Acc:2186]	-2.71	1.44
ENSG00000099953	MMP11	matrix metallopeptidase 11 (stromelysin 3) [Source:HGNC Symbol;Acc:7157]	-2.68	1.42
ENSG00000240694	PNMA2	paraneoplastic Ma antigen 2 [Source:HGNC Symbol;Acc:9159]	-2.66	1.41
ENSG00000170577	SIX2	SIX homeobox 2 [Source:HGNC Symbol;Acc:10888]	-2.59	1.37
ENSG00000221818	EBF2	early B-cell factor 2 [Source:HGNC Symbol;Acc:19090]	-2.59	1.37
ENSG00000224149			-2.59	1.37
ENSG00000126778	SIX1	SIX homeobox 1 [Source:HGNC Symbol;Acc:10887]	-2.54	1.35
ENSG00000182013	PNMAL1	paraneoplastic Ma antigen family-like 1 [Source:HGNC Symbol;Acc:25578]	-2.54	1.34
ENSG00000160801	PTH1R	parathyroid hormone 1 receptor [Source:HGNC Symbol;Acc:9608]	-2.53	1.34
ENSG00000178934	LGALS7B	lectin, galactoside-binding, soluble, 7B [Source:HGNC Symbol;Acc:34447]	-2.51	1.33
ENSG00000137809	ITGA11	integrin, alpha 11 [Source:HGNC Symbol;Acc:6136]	-2.5	1.32
ENSG00000186340	THBS2	thrombospondin 2 [Source:HGNC Symbol;Acc:11786]	-2.49	1.31
ENSG00000106333	PCOLCE	procollagen C-endopeptidase enhancer [Source:HGNC Symbol;Acc:8738]	-2.48	1.31
ENSG00000160161	CILP2	cartilage intermediate layer protein 2 [Source:HGNC Symbol;Acc:24213]	-2.48	1.31
ENSG00000272157			-2.47	1.3
ENSG00000173376	NDNF	neuron-derived neurotrophic factor [Source:HGNC Symbol;Acc:26256]	-2.46	1.3
ENSG00000173705	SUSD5	sushi domain containing 5 [Source:HGNC Symbol;Acc:29061]	-2.46	1.3
ENSG00000092009	CMA1	chymase 1, mast cell [Source:HGNC Symbol;Acc:2097]	-2.45	1.29
ENSG00000170624	SGCD	sarcoglycan, delta (35kDa dystrophin-associated glycoprotein) [Source:HGNC Symbol;Acc:10807]	-2.44	1.29
ENSG00000122420	PTGFR	prostaglandin F receptor (FP) [Source:HGNC Symbol;Acc:9600]	-2.42	1.28
ENSG00000171246	NPTX1	neuronal pentraxin I [Source:HGNC Symbol;Acc:7952]	-2.4	1.26
ENSG00000196557	CACNA1H	calcium channel, voltage-dependent, T type, alpha 1H subunit [Source:HGNC Symbol;Acc:1395]	-2.39	1.26

ENSG00000122121	XPNPEP2	X-prolyl aminopeptidase (aminopeptidase P) 2, membrane-bound [Source:HGNC Symbol;Acc:12823]	-2.38	1.25
ENSG00000163359	COL6A3	collagen, type VI, alpha 3 [Source:HGNC Symbol;Acc:2213]	-2.38	1.25
ENSG00000110076	NRXN2	neurexin 2 [Source:HGNC Symbol;Acc:8009]	-2.36	1.24
ENSG00000124212	PTGIS	prostaglandin I2 (prostacyclin) synthase [Source:HGNC Symbol;Acc:9603]	-2.36	1.24
ENSG00000183160	TMEM119	transmembrane protein 119 [Source:HGNC Symbol;Acc:27884]	-2.36	1.24
ENSG00000143867	OSR1	odd-skipped related transciption factor 1 [Source:HGNC Symbol;Acc:8111]	-2.34	1.23
ENSG00000070193	FGF10	fibroblast growth factor 10 [Source:HGNC Symbol;Acc:3666]	-2.34	1.22
ENSG00000162576	MXRA8	matrix-remodelling associated 8 [Source:HGNC Symbol;Acc:7542]	-2.34	1.22
ENSG00000169783	LINGO1	leucine rich repeat and Ig domain containing 1 [Source:HGNC Symbol;Acc:21205]	-2.34	1.22
ENSG00000204866	IGFL2	IGF-like family member 2 [Source:HGNC Symbol;Acc:32929]	-2.32	1.21
ENSG00000145681	HAPLN1	hyaluronan and proteoglycan link protein 1 [Source:HGNC Symbol;Acc:2380]	-2.3	1.2
ENSG00000188783	PRELP	proline/arginine-rich end leucine-rich repeat protein [Source:HGNC Symbol;Acc:9357]	-2.3	1.2
ENSG00000142156	COL6A1	collagen, type VI, alpha 1 [Source:HGNC Symbol;Acc:2211]	-2.28	1.19
ENSG00000197614	MFAP5	microfibrillar associated protein 5 [Source:HGNC Symbol;Acc:29673]	-2.27	1.18
ENSG00000140285	FGF7	fibroblast growth factor 7 [Source:HGNC Symbol;Acc:3685]	-2.27	1.18
ENSG00000166482	MFAP4	microfibrillar-associated protein 4 [Source:HGNC Symbol;Acc:7035]	-2.27	1.18
ENSG00000205795	CYS1	cystin 1 [Source:HGNC Symbol;Acc:18525]	-2.27	1.18
ENSG00000087245	MMP2	matrix metallopeptidase 2 (gelatinase A, 72kDa gelatinase, 72kDa type IV collagenase) [Source:HGNC Symbol;Acc:7166]	-2.26	1.17
ENSG00000142173	COL6A2	collagen, type VI, alpha 2 [Source:HGNC Symbol;Acc:2212]	-2.25	1.17
ENSG00000166963	MAP1A	microtubule-associated protein 1A [Source:HGNC Symbol;Acc:6835]	-2.23	1.16
ENSG00000172061	LRRC15	leucine rich repeat containing 15 [Source:HGNC Symbol;Acc:20818]	-2.23	1.16
ENSG00000115414	FN1	fibronectin 1 [Source:HGNC Symbol;Acc:3778]	-2.21	1.14
ENSG00000175445	LPL	lipoprotein lipase [Source:HGNC Symbol;Acc:6677]	-2.21	1.14
ENSG00000182771	GRID1	glutamate receptor, ionotropic, delta 1 [Source:HGNC Symbol;Acc:4575]	-2.2	1.14
ENSG00000164692	COL1A2	collagen, type I, alpha 2 [Source:HGNC Symbol;Acc:2198]	-2.19	1.13
ENSG00000127990	SGCE	sarcoglycan, epsilon [Source:HGNC Symbol;Acc:10808]	-2.18	1.13

ENSG00000136274	NACAD	NAC alpha domain containing [Source:HGNC Symbol;Acc:22196]	-2.18	1.13
ENSG00000162624	LHX8	LIM homeobox 8 [Source:HGNC Symbol;Acc:28838]	-2.18	1.13
ENSG00000104725	NEFL	neurofilament, light polypeptide [Source:HGNC Symbol;Acc:7739]	-2.18	1.12
ENSG00000158445	KCNB1	potassium voltage-gated channel, Shab-related subfamily, member 1 [Source:HGNC Symbol;Acc:6231]	-2.18	1.12
ENSG00000122691	TWIST1	twist family bHLH transcription factor 1 [Source:HGNC Symbol;Acc:12428]	-2.16	1.11
ENSG00000130032	PRRG3	proline rich Gla (G-carboxyglutamic acid) 3 (transmembrane) [Source:HGNC Symbol;Acc:30798]	-2.15	1.1
ENSG00000150051	MKX	mohawk homeobox [Source:HGNC Symbol;Acc:23729]	-2.14	1.1
ENSG00000168542	COL3A1	collagen, type III, alpha 1 [Source:HGNC Symbol;Acc:2201]	-2.13	1.09
ENSG00000184347	SLIT3	slit homolog 3 (Drosophila) [Source:HGNC Symbol;Acc:11087]	-2.12	1.09
ENSG00000006016	CRLF1	cytokine receptor-like factor 1 [Source:HGNC Symbol;Acc:2364]	-2.12	1.08
ENSG00000133110	POSTN	periostin, osteoblast specific factor [Source:HGNC Symbol;Acc:16953]	-2.12	1.08
ENSG00000198542	ITGBL1	integrin, beta-like 1 (with EGF-like repeat domains) [Source:HGNC Symbol;Acc:6164]	-2.11	1.08
ENSG00000244486	SCARF2	scavenger receptor class F, member 2 [Source:HGNC Symbol;Acc:19869]	-2.11	1.08
ENSG00000168779	SHOX2	short stature homeobox 2 [Source:HGNC Symbol;Acc:10854]	-2.1	1.07
ENSG00000153902	LGI4	leucine-rich repeat LGI family, member 4 [Source:HGNC Symbol;Acc:18712]	-2.09	1.06
ENSG00000044524	EPHA3	EPH receptor A3 [Source:HGNC Symbol;Acc:3387]	-2.08	1.06
ENSG00000134201	GSTM5	glutathione S-transferase mu 5 [Source:HGNC Symbol;Acc:4637]	-2.08	1.06
ENSG00000166793	YPEL4	yippee-like 4 (Drosophila) [Source:HGNC Symbol;Acc:18328]	-2.08	1.06
ENSG00000137273	FOXF2	forkhead box F2 [Source:HGNC Symbol;Acc:3810]	-2.07	1.05
ENSG00000196338	NLGN3	neuroligin 3 [Source:HGNC Symbol;Acc:14289]	-2.07	1.05
ENSG00000106624	AEBP1	AE binding protein 1 [Source:HGNC Symbol;Acc:303]	-2.07	1.05
ENSG00000197406	DIO3	deiodinase, iodothyronine, type III [Source:HGNC Symbol;Acc:2885]	-2.07	1.05
ENSG00000272761			-2.07	1.05
ENSG00000165617	DACT1	dishevelled-binding antagonist of beta-catenin 1 [Source:HGNC Symbol;Acc:17748]	-2.06	1.04
ENSG00000204262	COL5A2	collagen, type V, alpha 2 [Source:HGNC Symbol;Acc:2210]	-2.06	1.04
ENSG00000105472	CLEC11A	C-type lectin domain family 11, member A [Source:HGNC Symbol;Acc:10576]	-2.06	1.04

ENSG00000250167			-2.06	1.04
ENSG00000140937	CDH11	cadherin 11, type 2, OB-cadherin (osteoblast) [Source:HGNC Symbol;Acc:1750]	-2.04	1.03
ENSG00000183801	OLFML1	olfactomedin-like 1 [Source:HGNC Symbol;Acc:24473]	-2.04	1.03
ENSG00000106236	NPTX2	neuronal pentraxin II [Source:HGNC Symbol;Acc:7953]	-2.04	1.03
ENSG00000111799	COL12A1	collagen, type XII, alpha 1 [Source:HGNC Symbol;Acc:2188]	-2.03	1.02
ENSG00000163145	C1QTNF7	C1q and tumor necrosis factor related protein 7 [Source:HGNC Symbol;Acc:14342]	-2.03	1.02
ENSG00000152377	SPOCK1	sparc/osteonectin, cwcv and kazal-like domains proteoglycan (testican) 1 [Source:HGNC Symbol;Acc:11251]	-2.03	1.02
ENSG00000261327			-2.03	1.02
ENSG00000077782	FGFR1	fibroblast growth factor receptor 1 [Source:HGNC Symbol;Acc:3688]	-2.02	1.02
ENSG00000206052	DOK6	docking protein 6 [Source:HGNC Symbol;Acc:28301]	-2.02	1.01
ENSG00000154025	SLC5A10	solute carrier family 5 (sodium/sugar cotransporter), member 10 [Source:HGNC Symbol;Acc:23155]	-2.01	1.01
ENSG00000223485			-2.01	1.01
ENSG00000118729	CASQ2	calsequestrin 2 (cardiac muscle) [Source:HGNC Symbol;Acc:1513]	-2.01	1.01
ENSG00000135454	B4GALNT1	beta-1,4-N-acetyl-galactosaminyl transferase 1 [Source:HGNC Symbol;Acc:4117]	-2.01	1.01
ENSG00000143387	CTSK	cathepsin K [Source:HGNC Symbol;Acc:2536]	-2.00	1.00
ENSG00000149380	P4HA3	prolyl 4-hydroxylase, alpha polypeptide III [Source:HGNC Symbol;Acc:30135]	-1.98	0.99
ENSG00000153404	PLEKHG4B	pleckstrin homology domain containing, family G (with RhoGef domain) member 4B [Source:HGNC Symbol;Acc:29399]	-1.98	0.99
ENSG00000179954	SSC5D	scavenger receptor cysteine rich domain containing (5 domains) [Source:HGNC Symbol;Acc:26641]	-1.98	0.99
ENSG00000078053	AMPH	amphiphysin [Source:HGNC Symbol;Acc:471]	-1.98	0.98
ENSG00000105048	TNNT1	troponin T type 1 (skeletal, slow) [Source:HGNC Symbol;Acc:11948]	-1.97	0.98
ENSG00000154096	THY1	Thy-1 cell surface antigen [Source:HGNC Symbol;Acc:11801]	-1.97	0.98
ENSG00000172935	MRGPRF	MAS-related GPR, member F [Source:HGNC Symbol;Acc:24828]	-1.97	0.98
ENSG00000120820	GLT8D2	glycosyltransferase 8 domain containing 2 [Source:HGNC Symbol;Acc:24890]	-1.96	0.97
ENSG00000162631	NTNG1	netrin G1 [Source:HGNC Symbol;Acc:23319]	-1.96	0.97
ENSG00000166923	GREM1	gremlin 1, DAN family BMP antagonist [Source:HGNC Symbol;Acc:2001]	-1.96	0.97

ENSG00000233117	LINC00702	long intergenic non-protein coding RNA 702 [Source:HGNC Symbol;Acc:44676]	-1.95	0.97
ENSG00000151388	ADAMTS12	ADAM metallopeptidase with thrombospondin type 1 motif, 12 [Source:HGNC Symbol;Acc:14605]	-1.95	0.96
ENSG00000087116	ADAMTS2	ADAM metallopeptidase with thrombospondin type 1 motif, 2 [Source:HGNC Symbol;Acc:218]	-1.95	0.96
ENSG00000103546	SLC6A2	solute carrier family 6 (neurotransmitter transporter), member 2 [Source:HGNC Symbol;Acc:11048]	-1.93	0.95
ENSG00000113721	PDGFRB	platelet-derived growth factor receptor, beta polypeptide [Source:HGNC Symbol;Acc:8804]	-1.93	0.95
ENSG00000138080	EMILIN1	elastin microfibril interfacer 1 [Source:HGNC Symbol;Acc:19880]	-1.93	0.95
ENSG00000141756	FKBP10	FK506 binding protein 10, 65 kDa [Source:HGNC Symbol;Acc:18169]	-1.93	0.95
ENSG00000180660	MAB21L1	mab-21-like 1 (C. elegans) [Source:HGNC Symbol;Acc:6757]	-1.93	0.95
ENSG00000145244	CORIN	corin, serine peptidase [Source:HGNC Symbol;Acc:19012]	-1.92	0.94
ENSG00000100448	CTSG	cathepsin G [Source:HGNC Symbol;Acc:2532]	-1.92	0.94
ENSG00000146122	DAAM2	dishevelled associated activator of morphogenesis 2 [Source:HGNC Symbol;Acc:18143]	-1.92	0.94
ENSG00000182851	GPIHBP1	glycosylphosphatidylinositol anchored high density lipoprotein binding protein 1 [Source:HGNC Symbol;Acc:24945]	-1.92	0.94
ENSG00000185565	LSAMP	limbic system-associated membrane protein [Source:HGNC Symbol;Acc:6705]	-1.92	0.94
ENSG00000230630	DNM3OS	DNM3 opposite strand/antisense RNA [Source:HGNC Symbol;Acc:41228]	-1.92	0.94
ENSG00000145861	C1QTNF2	C1q and tumor necrosis factor related protein 2 [Source:HGNC Symbol;Acc:14325]	-1.91	0.94
ENSG00000106823	ECM2	extracellular matrix protein 2, female organ and adipocyte specific [Source:HGNC Symbol;Acc:3154]	-1.9	0.93
ENSG00000172260	NEGR1	neuronal growth regulator 1 [Source:HGNC Symbol;Acc:17302]	-1.9	0.93
ENSG00000088882	CPXM1	carboxypeptidase X (M14 family), member 1 [Source:HGNC Symbol;Acc:15771]	-1.9	0.93
ENSG00000103742	IGDCC4	immunoglobulin superfamily, DCC subclass, member 4 [Source:HGNC Symbol;Acc:13770]	-1.9	0.93
ENSG00000144649	FAM198A	family with sequence similarity 198, member A [Source:HGNC Symbol;Acc:24485]	-1.9	0.92
ENSG00000167994	RAB3IL1	RAB3A interacting protein (rabin3)-like 1 [Source:HGNC Symbol;Acc:9780]	-1.9	0.92
ENSG00000142552	RCN3	reticulocalbin 3, EF-hand calcium binding domain [Source:HGNC Symbol;Acc:21145]	-1.89	0.92
ENSG00000162745	OLFML2B	olfactomedin-like 2B [Source:HGNC Symbol;Acc:24558]	-1.89	0.92
ENSG00000172572	PDE3A	phosphodiesterase 3A, cGMP-inhibited [Source:HGNC Symbol;Acc:8778]	-1.89	0.92
ENSG00000100097	LGALS1	lectin, galactoside-binding, soluble, 1 [Source:HGNC Symbol;Acc:6561]	-1.89	0.92
ENSG00000149451	ADAM33	ADAM metallopeptidase domain 33 [Source:HGNC Symbol;Acc:15478]	-1.88	0.91

ENSG00000229867	STEAP3-AS1	STEAP3 antisense RNA 1 [Source:HGNC Symbol;Acc:41053]	-1.88	0.91
ENSG00000168079	SCARA5	scavenger receptor class A, member 5 (putative) [Source:HGNC Symbol;Acc:28701]	-1.88	0.91
ENSG00000102683	SGCG	sarcoglycan, gamma (35kDa dystrophin-associated glycoprotein) [Source:HGNC Symbol;Acc:10809]	-1.88	0.91
ENSG00000157150	TIMP4	TIMP metallopeptidase inhibitor 4 [Source:HGNC Symbol;Acc:11823]	-1.88	0.91
ENSG00000143196	DPT	dermatopontin [Source:HGNC Symbol;Acc:3011]	-1.87	0.91
ENSG00000182492	BGN	biglycan [Source:HGNC Symbol;Acc:1044]	-1.87	0.91
ENSG00000119888	EPCAM	epithelial cell adhesion molecule [Source:HGNC Symbol;Acc:11529]	-1.87	0.9
ENSG00000105419	MEIS3	Meis homeobox 3 [Source:HGNC Symbol;Acc:29537]	-1.85	0.89
ENSG00000149575	SCN2B	sodium channel, voltage-gated, type II, beta subunit [Source:HGNC Symbol;Acc:10589]	-1.85	0.89
ENSG00000152268	SPON1	spondin 1, extracellular matrix protein [Source:HGNC Symbol;Acc:11252]	-1.85	0.89
ENSG00000130635	COL5A1	collagen, type V, alpha 1 [Source:HGNC Symbol;Acc:2209]	-1.85	0.88
ENSG00000087495	PHACTR3	phosphatase and actin regulator 3 [Source:HGNC Symbol;Acc:15833]	-1.84	0.88
ENSG00000196368	NUDT11	nudix (nucleoside diphosphate linked moiety X)-type motif 11 [Source:HGNC Symbol;Acc:18011]	-1.84	0.88
ENSG00000157613	CREB3L1	cAMP responsive element binding protein 3-like 1 [Source:HGNC Symbol;Acc:18856]	-1.83	0.88
ENSG00000225614	ZNF469	zinc finger protein 469 [Source:HGNC Symbol;Acc:23216]	-1.83	0.88
ENSG00000176490	DIRAS1	DIRAS family, GTP-binding RAS-like 1 [Source:HGNC Symbol;Acc:19127]	-1.83	0.87
ENSG00000198963	RORB	RAR-related orphan receptor B [Source:HGNC Symbol;Acc:10259]	-1.83	0.87
ENSG00000102053	ZC3H12B	zinc finger CCCH-type containing 12B [Source:HGNC Symbol;Acc:17407]	-1.82	0.87
ENSG00000152049	KCNE4	potassium voltage-gated channel, Isk-related family, member 4 [Source:HGNC Symbol;Acc:6244]	-1.82	0.87
ENSG00000165124	SVEP1	sushi, von Willebrand factor type A, EGF and pentraxin domain containing 1 [Source:HGNC Symbol;Acc:15985]	-1.82	0.87
ENSG00000169439	SDC2	syndecan 2 [Source:HGNC Symbol;Acc:10659]	-1.82	0.87
ENSG00000112320	SOBP	sine oculis binding protein homolog (Drosophila) [Source:HGNC Symbol;Acc:29256]	-1.82	0.86
ENSG00000120645	IQSEC3	IQ motif and Sec7 domain 3 [Source:HGNC Symbol;Acc:29193]	-1.82	0.86
ENSG00000177098	SCN4B	sodium channel, voltage-gated, type IV, beta subunit [Source:HGNC Symbol;Acc:10592]	-1.81	0.86
ENSG00000117122	MFAP2	microfibrillar-associated protein 2 [Source:HGNC Symbol;Acc:7033]	-1.81	0.86
ENSG00000206538	VGLL3	vestigial like 3 (Drosophila) [Source:HGNC Symbol;Acc:24327]	-1.81	0.86

ENSG00000229645	LINC00341	long intergenic non-protein coding RNA 341 [Source:HGNC Symbol;Acc:20353]	-1.81	0.86
ENSG00000082196	C1QTNF3	C1q and tumor necrosis factor related protein 3 [Source:HGNC Symbol;Acc:14326]	-1.81	0.85
ENSG00000163520	FBLN2	fibulin 2 [Source:HGNC Symbol;Acc:3601]	-1.81	0.85
ENSG00000185532	PRKG1	protein kinase, cGMP-dependent, type I [Source:HGNC Symbol;Acc:9414]	-1.81	0.85
ENSG00000091986	CCDC80	coiled-coil domain containing 80 [Source:HGNC Symbol;Acc:30649]	-1.8	0.85
ENSG00000160539	PPAPDC3	phosphatidic acid phosphatase type 2 domain containing 3 [Source:HGNC Symbol;Acc:28174]	-1.8	0.85
ENSG00000116774	OLFML3	olfactomedin-like 3 [Source:HGNC Symbol;Acc:24956]	-1.8	0.85
ENSG00000237187	NR2F1-AS1	NR2F1 antisense RNA 1 [Source:HGNC Symbol;Acc:48622]	-1.8	0.85
ENSG00000109738	GLRB	glycine receptor, beta [Source:HGNC Symbol;Acc:4329]	-1.8	0.84
ENSG00000135218	CD36	CD36 molecule (thrombospondin receptor) [Source:HGNC Symbol;Acc:1663]	-1.79	0.84
ENSG00000167244	IGF2	insulin-like growth factor 2 (somatomedin A) [Source:HGNC Symbol;Acc:5466]	-1.79	0.84
ENSG00000113140	SPARC	secreted protein, acidic, cysteine-rich (osteonectin) [Source:HGNC Symbol;Acc:11219]	-1.79	0.84
ENSG00000112619	PRPH2	peripherin 2 (retinal degeneration, slow) [Source:HGNC Symbol;Acc:9942]	-1.78	0.83
ENSG00000103175	WFDC1	WAP four-disulfide core domain 1 [Source:HGNC Symbol;Acc:15466]	-1.78	0.83
ENSG00000162614	NEXN	nexilin (F actin binding protein) [Source:HGNC Symbol;Acc:29557]	-1.78	0.83
ENSG00000198795	ZNF521	zinc finger protein 521 [Source:HGNC Symbol;Acc:24605]	-1.78	0.83
ENSG00000151917	BEND6	BEN domain containing 6 [Source:HGNC Symbol;Acc:20871]	-1.78	0.83
ENSG00000163132	MSX1	msh homeobox 1 [Source:HGNC Symbol;Acc:7391]	-1.78	0.83
ENSG00000166073	GPR176	G protein-coupled receptor 176 [Source:HGNC Symbol;Acc:32370]	-1.77	0.83
ENSG00000174807	CD248	CD248 molecule, endosialin [Source:HGNC Symbol;Acc:18219]	-1.77	0.83
ENSG00000197921	HES5	hes family bHLH transcription factor 5 [Source:HGNC Symbol;Acc:19764]	-1.77	0.83
ENSG00000129009	ISLR	immunoglobulin superfamily containing leucine-rich repeat [Source:HGNC Symbol;Acc:6133]	-1.77	0.82
ENSG00000133083	DCLK1	doublecortin-like kinase 1 [Source:HGNC Symbol;Acc:2700]	-1.77	0.82
ENSG00000133216	EPHB2	EPH receptor B2 [Source:HGNC Symbol;Acc:3393]	-1.76	0.81
ENSG00000174348	PODN	podocan [Source:HGNC Symbol;Acc:23174]	-1.75	0.81
ENSG00000084636	COL16A1	collagen, type XVI, alpha 1 [Source:HGNC Symbol;Acc:2193]	-1.75	0.8

ENSG00000164920	OSR2	odd-skipped related transciption factor 2 [Source:HGNC Symbol;Acc:15830]	-1.75	0.8
ENSG00000143341	HMCN1	hemicentin 1 [Source:HGNC Symbol;Acc:19194]	-1.74	0.8
ENSG00000103196	CRISPLD2	cysteine-rich secretory protein LCCL domain containing 2 [Source:HGNC Symbol;Acc:25248]	-1.74	0.8
ENSG00000159713	TPPP3	tubulin polymerization-promoting protein family member 3 [Source:HGNC Symbol;Acc:24162]	-1.74	0.8
ENSG00000169122	FAM110B	family with sequence similarity 110, member B [Source:HGNC Symbol;Acc:28587]	-1.74	0.8
ENSG00000128591	FLNC	filamin C, gamma [Source:HGNC Symbol;Acc:3756]	-1.73	0.79
ENSG00000145362	ANK2	ankyrin 2, neuronal [Source:HGNC Symbol;Acc:493]	-1.73	0.79
ENSG00000078549	ADCYAP1R1	adenylate cyclase activating polypeptide 1 (pituitary) receptor type I [Source:HGNC Symbol;Acc:242]	-1.73	0.79
ENSG00000131711	MAP1B	microtubule-associated protein 1B [Source:HGNC Symbol;Acc:6836]	-1.73	0.79
ENSG00000073712	FERMT2	fermitin family member 2 [Source:HGNC Symbol;Acc:15767]	-1.72	0.79
ENSG00000137070	IL11RA	interleukin 11 receptor, alpha [Source:HGNC Symbol;Acc:5967]	-1.72	0.79
ENSG00000117600		Lipid phosphate phosphatase-related protein type 4 [Source:UniProtKB/Swiss-Prot;Acc:Q7Z2D5]	-1.72	0.78
ENSG00000100968	NFATC4	nuclear factor of activated T-cells, cytoplasmic, calcineurin-dependent 4 [Source:HGNC Symbol;Acc:7778]	-1.72	0.78
ENSG00000139971	C14orf37	chromosome 14 open reading frame 37 [Source:HGNC Symbol;Acc:19846]	-1.72	0.78
ENSG00000143515	ATP8B2	ATPase, aminophospholipid transporter, class I, type 8B, member 2 [Source:HGNC Symbol;Acc:13534]	-1.71	0.78
ENSG00000164932	CTHRC1	collagen triple helix repeat containing 1 [Source:HGNC Symbol;Acc:18831]	-1.71	0.78
ENSG00000238142			-1.71	0.77
ENSG00000136859	ANGPTL2	angiopoietin-like 2 [Source:HGNC Symbol;Acc:490]	-1.7	0.77
ENSG00000116194	ANGPTL1	angiopoietin-like 1 [Source:HGNC Symbol;Acc:489]	-1.7	0.77
ENSG00000185633	NDUFA4L2	NADH dehydrogenase (ubiquinone) 1 alpha subcomplex, 4-like 2 [Source:HGNC Symbol;Acc:29836]	-1.7	0.77
ENSG00000138696	BMPR1B	bone morphogenetic protein receptor, type IB [Source:HGNC Symbol;Acc:1077]	-1.69	0.76
ENSG00000174332	GLIS1	GLIS family zinc finger 1 [Source:HGNC Symbol;Acc:29525]	-1.69	0.76
ENSG00000185634	SHC4	SHC (Src homology 2 domain containing) family, member 4 [Source:HGNC Symbol;Acc:16743]	-1.69	0.76
ENSG00000125378	BMP4	bone morphogenetic protein 4 [Source:HGNC Symbol;Acc:1071]	-1.68	0.75
ENSG00000169515	CCDC8	coiled-coil domain containing 8 [Source:HGNC Symbol;Acc:25367]	-1.68	0.75

ENSG00000162733	DDR2	discoidin domain receptor tyrosine kinase 2 [Source:HGNC Symbol;Acc:2731]	-1.68	0.74
ENSG00000100505	TRIM9	tripartite motif containing 9 [Source:HGNC Symbol;Acc:16288]	-1.66	0.73
ENSG00000017427	IGF1	insulin-like growth factor 1 (somatomedin C) [Source:HGNC Symbol;Acc:5464]	-1.66	0.73
ENSG00000038427	VCAN	versican [Source:HGNC Symbol;Acc:2464]	-1.66	0.73
ENSG00000157399	ARSE	arylsulfatase E (chondrodysplasia punctata 1) [Source:HGNC Symbol;Acc:719]	-1.66	0.73
ENSG00000227496			-1.65	0.72
ENSG00000121743	GJA3	gap junction protein, alpha 3, 46kDa [Source:HGNC Symbol;Acc:4277]	-1.65	0.72
ENSG00000165323	FAT3	FAT atypical cadherin 3 [Source:HGNC Symbol;Acc:23112]	-1.65	0.72
ENSG00000168675	LDLRAD4	low density lipoprotein receptor class A domain containing 4 [Source:HGNC Symbol;Acc:1224]	-1.65	0.72
ENSG00000189129	PLAC9	placenta-specific 9 [Source:HGNC Symbol;Acc:19255]	-1.65	0.72
ENSG00000224592			-1.65	0.72
ENSG00000128606	LRRC17	leucine rich repeat containing 17 [Source:HGNC Symbol;Acc:16895]	-1.65	0.72
ENSG00000163909	HEYL	hes-related family bHLH transcription factor with YRPW motif-like [Source:HGNC Symbol;Acc:4882]	-1.65	0.72
ENSG00000171451	DSEL	dermatan sulfate epimerase-like [Source:HGNC Symbol;Acc:18144]	-1.65	0.72
ENSG00000131471	AOC3	amine oxidase, copper containing 3 [Source:HGNC Symbol;Acc:550]	-1.64	0.72
ENSG00000187955	COL14A1	collagen, type XIV, alpha 1 [Source:HGNC Symbol;Acc:2191]	-1.64	0.72
ENSG00000078098	FAP	fibroblast activation protein, alpha [Source:HGNC Symbol;Acc:3590]	-1.64	0.71
ENSG00000020181	GPR124	G protein-coupled receptor 124 [Source:HGNC Symbol;Acc:17849]	-1.64	0.71
ENSG00000186832	KRT16	keratin 16 [Source:HGNC Symbol;Acc:6423]	-1.64	0.71
ENSG00000026025	VIM	vimentin [Source:HGNC Symbol;Acc:12692]	-1.63	0.71
ENSG00000154277	UCHL1	ubiquitin carboxyl-terminal esterase L1 (ubiquitin thiolesterase) [Source:HGNC Symbol;Acc:12513]	-1.63	0.71
ENSG00000186310	NAP1L3	nucleosome assembly protein 1-like 3 [Source:HGNC Symbol;Acc:7639]	-1.63	0.71
ENSG00000068615	REEP1	receptor accessory protein 1 [Source:HGNC Symbol;Acc:25786]	-1.63	0.71
ENSG00000115468	EFHD1	EF-hand domain family, member D1 [Source:HGNC Symbol;Acc:29556]	-1.63	0.7
ENSG00000273477			-1.63	0.7
ENSG00000152767	FARP1	FERM, RhoGEF (ARHGEF) and pleckstrin domain protein 1 (chondrocyte-derived) [Source:HGNC	-1.63	0.7

		Symbol;Acc:3591]		
ENSG00000125384	PTGER2	prostaglandin E receptor 2 (subtype EP2), 53kDa [Source:HGNC Symbol;Acc:9594]	-1.62	0.7
ENSG00000236256	DIAPH2-AS1	DIAPH2 antisense RNA 1 [Source:HGNC Symbol;Acc:16972]	-1.62	0.7
ENSG00000138944	KIAA1644	KIAA1644 [Source:HGNC Symbol;Acc:29335]	-1.62	0.69
ENSG00000112208	BAG2	BCL2-associated athanogene 2 [Source:HGNC Symbol;Acc:938]	-1.61	0.69
ENSG00000133466	C1QTNF6	C1q and tumor necrosis factor related protein 6 [Source:HGNC Symbol;Acc:14343]	-1.61	0.69
ENSG00000135835	KIAA1614	KIAA1614 [Source:HGNC Symbol;Acc:29327]	-1.61	0.69
ENSG00000109099	PMP22	peripheral myelin protein 22 [Source:HGNC Symbol;Acc:9118]	-1.61	0.69
ENSG00000134760	DSG1	desmoglein 1 [Source:HGNC Symbol;Acc:3048]	-1.61	0.69
ENSG00000165868	HSPA12A	heat shock 70kDa protein 12A [Source:HGNC Symbol;Acc:19022]	-1.61	0.69
ENSG00000132622	HSPA12B	heat shock 70kD protein 12B [Source:HGNC Symbol;Acc:16193]	-1.61	0.68
ENSG00000206190	ATP10A	ATPase, class V, type 10A [Source:HGNC Symbol;Acc:13542]	-1.59	0.67
ENSG00000063438	AHRR	aryl-hydrocarbon receptor repressor [Source:HGNC Symbol;Acc:346]	-1.59	0.67
ENSG00000107821	KAZALD1	Kazal-type serine peptidase inhibitor domain 1 [Source:HGNC Symbol;Acc:25460]	-1.59	0.67
ENSG00000132386	SERPINF1	serpin peptidase inhibitor, clade F (alpha-2 antiplasmin, pigment epithelium derived factor), member 1 [Source:HGNC Symbol;Acc:8824]	-1.59	0.67
ENSG00000104324	CPQ	carboxypeptidase Q [Source:HGNC Symbol;Acc:16910]	-1.58	0.66
ENSG00000150687	PRSS23	protease, serine, 23 [Source:HGNC Symbol;Acc:14370]	-1.58	0.66
ENSG00000147465	STAR	steroidogenic acute regulatory protein [Source:HGNC Symbol;Acc:11359]	-1.58	0.66
ENSG00000137672	TRPC6	transient receptor potential cation channel, subfamily C, member 6 [Source:HGNC Symbol;Acc:12338]	-1.58	0.66
ENSG00000197467	COL13A1	collagen, type XIII, alpha 1 [Source:HGNC Symbol;Acc:2190]	-1.58	0.66
ENSG00000071909	MYO3B	myosin IIIB [Source:HGNC Symbol;Acc:15576]	-1.57	0.66
ENSG00000147027	TMEM47	transmembrane protein 47 [Source:HGNC Symbol;Acc:18515]	-1.57	0.66
ENSG00000198835	GJC2	gap junction protein, gamma 2, 47kDa [Source:HGNC Symbol;Acc:17494]	-1.57	0.65
ENSG00000239492	FAM25HP	family with sequence similarity 25, member H pseudogene [Source:HGNC Symbol;Acc:23591]	-1.57	0.65
ENSG00000107736	CDH23	cadherin-related 23 [Source:HGNC Symbol;Acc:13733]	-1.56	0.65

ENSG00000154856	APCDD1	adenomatosis polyposis coli down-regulated 1 [Source:HGNC Symbol;Acc:15718]	-1.56	0.65
ENSG00000182667	NTM	neurotrimin [Source:HGNC Symbol;Acc:17941]	-1.56	0.65
ENSG00000035862	TIMP2	TIMP metallopeptidase inhibitor 2 [Source:HGNC Symbol;Acc:11821]	-1.56	0.64
ENSG00000164294	GPX8	glutathione peroxidase 8 (putative) [Source:HGNC Symbol;Acc:33100]	-1.56	0.64
ENSG00000255874	LINC00346	long intergenic non-protein coding RNA 346 [Source:HGNC Symbol;Acc:27492]	-1.56	0.64
ENSG00000232040	SCAND3	SCAN domain containing 3 [Source:HGNC Symbol;Acc:13851]	-1.56	0.64
ENSG00000132932	ATP8A2	ATPase, aminophospholipid transporter, class I, type 8A, member 2 [Source:HGNC Symbol;Acc:13533]	-1.56	0.64
ENSG00000166780	C16orf45	chromosome 16 open reading frame 45 [Source:HGNC Symbol;Acc:19213]	-1.56	0.64
ENSG00000119865	CNRIP1	cannabinoid receptor interacting protein 1 [Source:HGNC Symbol;Acc:24546]	-1.55	0.63
ENSG00000130224	LRCH2	leucine-rich repeats and calponin homology (CH) domain containing 2 [Source:HGNC Symbol;Acc:29292]	-1.55	0.63
ENSG00000128594	LRRC4	leucine rich repeat containing 4 [Source:HGNC Symbol;Acc:15586]	-1.55	0.63
ENSG00000156113	KCNMA1	potassium large conductance calcium-activated channel, subfamily M, alpha member 1 [Source:HGNC Symbol;Acc:6284]	-1.55	0.63
ENSG00000175183	CSRP2	cysteine and glycine-rich protein 2 [Source:HGNC Symbol;Acc:2470]	-1.55	0.63
ENSG00000149294	NCAM1	neural cell adhesion molecule 1 [Source:HGNC Symbol;Acc:7656]	-1.54	0.63
ENSG00000117016	RIMS3	regulating synaptic membrane exocytosis 3 [Source:HGNC Symbol;Acc:21292]	-1.54	0.62
ENSG00000152953	STK32B	serine/threonine kinase 32B [Source:HGNC Symbol;Acc:14217]	-1.54	0.62
ENSG00000060709	RIMBP2	RIMS binding protein 2 [Source:HGNC Symbol;Acc:30339]	-1.54	0.62
ENSG00000151778	SERP2	stress-associated endoplasmic reticulum protein family member 2 [Source:HGNC Symbol;Acc:20607]	-1.54	0.62
ENSG00000165633	VSTM4	V-set and transmembrane domain containing 4 [Source:HGNC Symbol;Acc:26470]	-1.54	0.62
ENSG00000166033	HTRA1	HtrA serine peptidase 1 [Source:HGNC Symbol;Acc:9476]	-1.54	0.62
ENSG00000226674	TEX41	testis expressed 41 (non-protein coding) [Source:HGNC Symbol;Acc:48667]	-1.54	0.62
ENSG00000184304	PRKD1	protein kinase D1 [Source:HGNC Symbol;Acc:9407]	-1.53	0.61
ENSG00000196139	AKR1C3	aldo-keto reductase family 1, member C3 [Source:HGNC Symbol;Acc:386]	-1.53	0.61
ENSG00000197256	KANK2	KN motif and ankyrin repeat domains 2 [Source:HGNC Symbol;Acc:29300]	-1.53	0.61
ENSG00000178033	FAM26E	family with sequence similarity 26, member E [Source:HGNC Symbol;Acc:21568]	-1.53	0.61

ENSG00000122707	RECK	reversion-inducing-cysteine-rich protein with kazal motifs [Source:HGNC Symbol;Acc:11345]	-1.52	0.61
ENSG00000145824	CXCL14	chemokine (C-X-C motif) ligand 14 [Source:HGNC Symbol;Acc:10640]	-1.52	0.61
ENSG00000113657	DPYSL3	dihydropyrimidinase-like 3 [Source:HGNC Symbol;Acc:3015]	-1.52	0.6
ENSG00000198910	L1CAM	L1 cell adhesion molecule [Source:HGNC Symbol;Acc:6470]	-1.52	0.6
ENSG00000154027	AK5	adenylate kinase 5 [Source:HGNC Symbol;Acc:365]	-1.51	0.6
ENSG00000172348	RCAN2	regulator of calcineurin 2 [Source:HGNC Symbol;Acc:3041]	-1.51	0.6
ENSG00000183496	MEX3B	mex-3 RNA binding family member B [Source:HGNC Symbol;Acc:25297]	-1.51	0.6
ENSG00000186847	KRT14	keratin 14 [Source:HGNC Symbol;Acc:6416]	-1.51	0.6
ENSG00000198892	SHISA4	shisa family member 4 [Source:HGNC Symbol;Acc:27139]	-1.51	0.59
ENSG00000122870	BICC1	bicaudal C homolog 1 (Drosophila) [Source:HGNC Symbol;Acc:19351]	-1.51	0.59
ENSG00000139329	LUM	lumican [Source:HGNC Symbol;Acc:6724]	-1.51	0.59
ENSG00000183114	FAM43B	family with sequence similarity 43, member B [Source:HGNC Symbol;Acc:31791]	-1.51	0.59
ENSG00000099864	PALM	paralemmin [Source:HGNC Symbol;Acc:8594]	-1.5	0.59
ENSG00000242732	RGAG4	retrotransposon gag domain containing 4 [Source:HGNC Symbol;Acc:29430]	-1.5	0.59
ENSG00000183722	LHFP	lipoma HMGIC fusion partner [Source:HGNC Symbol;Acc:6586]	-1.5	0.59
ENSG00000198121	LPAR1	lysophosphatidic acid receptor 1 [Source:HGNC Symbol;Acc:3166]	-1.5	0.59
ENSG00000154153	FAM134B	family with sequence similarity 134, member B [Source:HGNC Symbol;Acc:25964]	-1.5	0.58
ENSG00000227051	C14orf132	chromosome 14 open reading frame 132 [Source:HGNC Symbol;Acc:20346]	-1.5	0.58
ENSG00000174099	MSRB3	methionine sulfoxide reductase B3 [Source:HGNC Symbol;Acc:27375]	-1.5	0.58
ENSG00000110811	LEPREL2	leprecan-like 2 [Source:HGNC Symbol;Acc:19318]	-1.49	0.58
ENSG00000131386	GALNT15	UDP-N-acetyl-alpha-D-galactosamine:polypeptide N-acetylgalactosaminyltransferase 15 [Source:HGNC Symbol;Acc:21531]	-1.49	0.58
ENSG00000105088	OLFM2	olfactomedin 2 [Source:HGNC Symbol;Acc:17189]	-1.49	0.57
ENSG00000125864	BFSP1	beaded filament structural protein 1, filensin [Source:HGNC Symbol;Acc:1040]	-1.49	0.57
ENSG00000164176	EDIL3	EGF-like repeats and discoidin I-like domains 3 [Source:HGNC Symbol;Acc:3173]	-1.49	0.57
ENSG00000088543	C3orf18	chromosome 3 open reading frame 18 [Source:HGNC Symbol;Acc:24837]	-1.48	0.57
ENSG00000116132	PRRX1	paired related homeobox 1 [Source:HGNC Symbol;Acc:9142]	-1.48	0.56

ENSG00000162944	RFTN2	raftlin family member 2 [Source:HGNC Symbol;Acc:26402]	-1.48	0.56
ENSG00000163083	INHBB	inhibin, beta B [Source:HGNC Symbol;Acc:6067]	-1.48	0.56
ENSG00000183853	KIRREL	kin of IRRE like (Drosophila) [Source:HGNC Symbol;Acc:15734]	-1.48	0.56
ENSG00000079150	FKBP7	FK506 binding protein 7 [Source:HGNC Symbol;Acc:3723]	-1.47	0.56
ENSG00000140682	TGFB1I1	transforming growth factor beta 1 induced transcript 1 [Source:HGNC Symbol;Acc:11767]	-1.47	0.56
ENSG00000126803	HSPA2	heat shock 70kDa protein 2 [Source:HGNC Symbol;Acc:5235]	-1.47	0.55
ENSG00000130956	HABP4	hyaluronan binding protein 4 [Source:HGNC Symbol;Acc:17062]	-1.47	0.55
ENSG00000197380	DACT3	dishevelled-binding antagonist of beta-catenin 3 [Source:HGNC Symbol;Acc:30745]	-1.47	0.55
ENSG00000117020	AKT3	v-akt murine thymoma viral oncogene homolog 3 [Source:HGNC Symbol;Acc:393]	-1.46	0.55
ENSG00000022267	FHL1	four and a half LIM domains 1 [Source:HGNC Symbol;Acc:3702]	-1.45	0.54
ENSG00000159674	SPON2	spondin 2, extracellular matrix protein [Source:HGNC Symbol;Acc:11253]	-1.45	0.54
ENSG00000218305			-1.45	0.54
ENSG00000114853	ZBTB47	zinc finger and BTB domain containing 47 [Source:HGNC Symbol;Acc:26955]	-1.45	0.54
ENSG00000050165	DKK3	dickkopf WNT signaling pathway inhibitor 3 [Source:HGNC Symbol;Acc:2893]	-1.45	0.53
ENSG00000074211	PPP2R2C	protein phosphatase 2, regulatory subunit B, gamma [Source:HGNC Symbol;Acc:9306]	-1.45	0.53
ENSG00000149257	SERPINH1	serpin peptidase inhibitor, clade H (heat shock protein 47), member 1, (collagen binding protein 1) [Source:HGNC Symbol;Acc:1546]	-1.45	0.53
ENSG00000184785	SMIM10	small integral membrane protein 10 [Source:HGNC Symbol;Acc:41913]	-1.45	0.53
ENSG00000185668	POU3F1	POU class 3 homeobox 1 [Source:HGNC Symbol;Acc:9214]	-1.45	0.53
ENSG00000076706	MCAM	melanoma cell adhesion molecule [Source:HGNC Symbol;Acc:6934]	-1.45	0.53
ENSG00000121691	CAT	catalase [Source:HGNC Symbol;Acc:1516]	-1.45	0.53
ENSG00000141753	IGFBP4	insulin-like growth factor binding protein 4 [Source:HGNC Symbol;Acc:5473]	-1.45	0.53
ENSG00000165424	ZCCHC24	zinc finger, CCHC domain containing 24 [Source:HGNC Symbol;Acc:26911]	-1.44	0.53
ENSG00000177453	NIM1K	NIM1 serine/threonine protein kinase [Source:HGNC Symbol;Acc:28646]	-1.44	0.53
ENSG00000163431	LMOD1	leiomodin 1 (smooth muscle) [Source:HGNC Symbol;Acc:6647]	-1.44	0.53
ENSG00000107796	ACTA2	actin, alpha 2, smooth muscle, aorta [Source:HGNC Symbol;Acc:130]	-1.44	0.52
ENSG00000122824	NUDT10	nudix (nucleoside diphosphate linked moiety X)-type motif 10 [Source:HGNC Symbol;Acc:17621]	-1.44	0.52

ENSG00000149591	TAGLN	transgelin [Source:HGNC Symbol;Acc:11553]	-1.44	0.52
ENSG00000109472	CPE	carboxypeptidase E [Source:HGNC Symbol;Acc:2303]	-1.44	0.52
ENSG00000253304	TMEM200B	transmembrane protein 200B [Source:HGNC Symbol;Acc:33785]	-1.44	0.52
ENSG00000166086	JAM3	junctional adhesion molecule 3 [Source:HGNC Symbol;Acc:15532]	-1.43	0.52
ENSG00000144355	DLX1	distal-less homeobox 1 [Source:HGNC Symbol;Acc:2914]	-1.43	0.52
ENSG00000229619	MBNL1-AS1	MBNL1 antisense RNA 1 [Source:HGNC Symbol;Acc:44584]	-1.43	0.52
ENSG00000235257			-1.43	0.52
ENSG00000158747	NBL1	neuroblastoma 1, DAN family BMP antagonist [Source:HGNC Symbol;Acc:7650]	-1.43	0.51
ENSG00000188613	NANOS1	nanos homolog 1 (Drosophila) [Source:HGNC Symbol;Acc:23044]	-1.43	0.51
ENSG00000142227	EMP3	epithelial membrane protein 3 [Source:HGNC Symbol;Acc:3335]	-1.42	0.51
ENSG00000161940	BCL6B	B-cell CLL/lymphoma 6, member B [Source:HGNC Symbol;Acc:1002]	-1.42	0.51
ENSG00000106976	DNM1	dynamin 1 [Source:HGNC Symbol;Acc:2972]	-1.42	0.5
ENSG00000162706	CADM3	cell adhesion molecule 3 [Source:HGNC Symbol;Acc:17601]	-1.42	0.5
ENSG00000259877			-1.42	0.5
ENSG00000264868			-1.42	0.5
ENSG00000003137	CYP26B1	cytochrome P450, family 26, subfamily B, polypeptide 1 [Source:HGNC Symbol;Acc:20581]	-1.42	0.5
ENSG00000107186	MPDZ	multiple PDZ domain protein [Source:HGNC Symbol;Acc:7208]	-1.42	0.5
ENSG00000159899	NPR2	natriuretic peptide receptor B/guanylate cyclase B (atrionatriuretic peptide receptor B) [Source:HGNC Symbol;Acc:7944]	-1.41	0.5
ENSG00000240771	ARHGEF25	Rho guanine nucleotide exchange factor (GEF) 25 [Source:HGNC Symbol;Acc:30275]	-1.41	0.5
ENSG00000141696	LEPREL4	leprecan-like 4 [Source:HGNC Symbol;Acc:16946]	-1.41	0.49
ENSG00000101335	MYL9	myosin, light chain 9, regulatory [Source:HGNC Symbol;Acc:15754]	-1.4	0.49
ENSG00000157680	DGKI	diacylglycerol kinase, iota [Source:HGNC Symbol;Acc:2855]	-1.4	0.49
ENSG00000120471	TP53AIP1	tumor protein p53 regulated apoptosis inducing protein 1 [Source:HGNC Symbol;Acc:29984]	-1.4	0.49
ENSG00000144369	FAM171B	family with sequence similarity 171, member B [Source:HGNC Symbol;Acc:29412]	-1.39	0.48
ENSG00000151617	EDNRA	endothelin receptor type A [Source:HGNC Symbol;Acc:3179]	-1.39	0.47
ENSG00000137267	TUBB2A	tubulin, beta 2A class IIa [Source:HGNC Symbol;Acc:12412]	-1.39	0.47

ENSG00000140416	TPM1	tropomyosin 1 (alpha) [Source:HGNC Symbol;Acc:12010]	-1.39	0.47
ENSG00000162512	SDC3	syndecan 3 [Source:HGNC Symbol;Acc:10660]	-1.39	0.47
ENSG00000180354	MTURN	maturin, neural progenitor differentiation regulator homolog (Xenopus) [Source:HGNC Symbol;Acc:25457]	-1.39	0.47
ENSG00000049323	LTBP1	latent transforming growth factor beta binding protein 1 [Source:HGNC Symbol;Acc:6714]	-1.39	0.47
ENSG00000125170	DOK4	docking protein 4 [Source:HGNC Symbol;Acc:19868]	-1.38	0.47
ENSG00000121898	CPXM2	carboxypeptidase X (M14 family), member 2 [Source:HGNC Symbol;Acc:26977]	-1.38	0.46
ENSG00000067840	PDZD4	PDZ domain containing 4 [Source:HGNC Symbol;Acc:21167]	-1.38	0.46
ENSG00000180694	TMEM64	transmembrane protein 64 [Source:HGNC Symbol;Acc:25441]	-1.38	0.46
ENSG00000155254	MARVELD1	MARVEL domain containing 1 [Source:HGNC Symbol;Acc:28674]	-1.37	0.46
ENSG00000174080	CTSF	cathepsin F [Source:HGNC Symbol;Acc:2531]	-1.37	0.46
ENSG00000213062			-1.37	0.45
ENSG00000138172	CALHM2	calcium homeostasis modulator 2 [Source:HGNC Symbol;Acc:23493]	-1.37	0.45
ENSG00000183578	TNFAIP8L3	tumor necrosis factor, alpha-induced protein 8-like 3 [Source:HGNC Symbol;Acc:20620]	-1.36	0.45
ENSG00000181264	TMEM136	transmembrane protein 136 [Source:HGNC Symbol;Acc:28280]	-1.36	0.45
ENSG00000154330	PGM5	phosphoglucomutase 5 [Source:HGNC Symbol;Acc:8908]	-1.36	0.44
ENSG00000144730	IL17RD	interleukin 17 receptor D [Source:HGNC Symbol;Acc:17616]	-1.35	0.43
ENSG00000175274	TP53I11	tumor protein p53 inducible protein 11 [Source:HGNC Symbol;Acc:16842]	-1.35	0.43
ENSG00000198729	PPP1R14C	protein phosphatase 1, regulatory (inhibitor) subunit 14C [Source:HGNC Symbol;Acc:14952]	-1.35	0.43
ENSG00000080573	COL5A3	collagen, type V, alpha 3 [Source:HGNC Symbol;Acc:14864]	-1.34	0.43
ENSG00000180769	WDFY3-AS2	WDFY3 antisense RNA 2 [Source:HGNC Symbol;Acc:21603]	-1.34	0.43
ENSG00000071967	CYBRD1	cytochrome b reductase 1 [Source:HGNC Symbol;Acc:20797]	-1.34	0.42
ENSG00000142686	C1orf216	chromosome 1 open reading frame 216 [Source:HGNC Symbol;Acc:26800]	-1.34	0.42
ENSG00000178573	MAF	v-maf avian musculoaponeurotic fibrosarcoma oncogene homolog [Source:HGNC Symbol;Acc:6776]	-1.34	0.42
ENSG00000135736	CCDC102A	coiled-coil domain containing 102A [Source:HGNC Symbol;Acc:28097]	-1.34	0.42
ENSG00000148541	FAM13C	family with sequence similarity 13, member C [Source:HGNC Symbol;Acc:19371]	-1.33	0.42
ENSG00000171016	PYGO1	pygopus family PHD finger 1 [Source:HGNC Symbol;Acc:30256]	-1.33	0.41

ENSG00000134986	NREP	neuronal regeneration related protein [Source:HGNC Symbol;Acc:16834]	-1.33	0.41
ENSG00000166974	MAPRE2	microtubule-associated protein, RP/EB family, member 2 [Source:HGNC Symbol;Acc:6891]	-1.33	0.41
ENSG00000100234	TIMP3	TIMP metallopeptidase inhibitor 3 [Source:HGNC Symbol;Acc:11822]	-1.33	0.41
ENSG00000166997	CNPY4	canopy FGF signaling regulator 4 [Source:HGNC Symbol;Acc:28631]	-1.33	0.41
ENSG00000167552	TUBA1A	tubulin, alpha 1a [Source:HGNC Symbol;Acc:20766]	-1.33	0.41
ENSG00000173517	PEAK1	pseudopodium-enriched atypical kinase 1 [Source:HGNC Symbol;Acc:29431]	-1.33	0.41
ENSG00000122786	CALD1	caldesmon 1 [Source:HGNC Symbol;Acc:1441]	-1.32	0.41
ENSG00000101298	SNPH	syntaphilin [Source:HGNC Symbol;Acc:15931]	-1.32	0.4
ENSG00000126947	ARMCX1	armadillo repeat containing, X-linked 1 [Source:HGNC Symbol;Acc:18073]	-1.32	0.4
ENSG00000165698	C9orf9	chromosome 9 open reading frame 9 [Source:HGNC Symbol;Acc:1367]	-1.32	0.4
ENSG00000166402	TUB	tubby bipartite transcription factor [Source:HGNC Symbol;Acc:12406]	-1.32	0.4
ENSG00000072422	RHOBTB1	Rho-related BTB domain containing 1 [Source:HGNC Symbol;Acc:18738]	-1.32	0.4
ENSG00000198774	RASSF9	Ras association (RalGDS/AF-6) domain family (N-terminal) member 9 [Source:HGNC Symbol;Acc:15739]	-1.32	0.4
ENSG00000235217	TSPY26P	testis specific protein, Y-linked 26, pseudogene [Source:HGNC Symbol;Acc:16256]	-1.32	0.4
ENSG00000162694	EXTL2	exostosin-like glycosyltransferase 2 [Source:HGNC Symbol;Acc:3516]	-1.32	0.4
ENSG00000183580	FBXL7	F-box and leucine-rich repeat protein 7 [Source:HGNC Symbol;Acc:13604]	-1.32	0.4
ENSG00000116667	C1orf21	chromosome 1 open reading frame 21 [Source:HGNC Symbol;Acc:15494]	-1.31	0.39
ENSG00000101000	PROCR	protein C receptor, endothelial [Source:HGNC Symbol;Acc:9452]	-1.31	0.39
ENSG00000260966			-1.31	0.39
ENSG00000132000	PODNL1	podocan-like 1 [Source:HGNC Symbol;Acc:26275]	-1.31	0.39
ENSG00000136810	TXN	thioredoxin [Source:HGNC Symbol;Acc:12435]	-1.31	0.39
ENSG00000145431	PDGFC	platelet derived growth factor C [Source:HGNC Symbol;Acc:8801]	-1.31	0.38
ENSG00000149212	SESN3	sestrin 3 [Source:HGNC Symbol;Acc:23060]	-1.31	0.38
ENSG00000100036	SLC35E4	solute carrier family 35, member E4 [Source:HGNC Symbol;Acc:17058]	-1.3	0.38
ENSG00000151692	RNF144A	ring finger protein 144A [Source:HGNC Symbol;Acc:20457]	-1.3	0.38
ENSG00000106003	LFNG	LFNG O-fucosylpeptide 3-beta-N-acetylglucosaminyltransferase [Source:HGNC Symbol;Acc:6560]	-1.3	0.37

ENSG00000155265	GOLGA7B	golgin A7 family, member B [Source:HGNC Symbol;Acc:31668]	-1.3	0.37
ENSG00000162849	KIF26B	kinesin family member 26B [Source:HGNC Symbol;Acc:25484]	-1.3	0.37
ENSG00000065534	MYLK	myosin light chain kinase [Source:HGNC Symbol;Acc:7590]	-1.29	0.37
ENSG00000019144	PHLDB1	pleckstrin homology-like domain, family B, member 1 [Source:HGNC Symbol;Acc:23697]	-1.29	0.37
ENSG00000214357	NEURL1B	neuralized E3 ubiquitin protein ligase 1B [Source:HGNC Symbol;Acc:35422]	-1.29	0.37
ENSG00000107819	SFXN3	sideroflexin 3 [Source:HGNC Symbol;Acc:16087]	-1.29	0.37
ENSG00000107679	PLEKHA1	pleckstrin homology domain containing, family A (phosphoinositide binding specific) member 1 [Source:HGNC Symbol;Acc:14335]	-1.29	0.36
ENSG00000008256	CYTH3	cytohesin 3 [Source:HGNC Symbol;Acc:9504]	-1.28	0.36
ENSG00000177932	ZNF354C	zinc finger protein 354C [Source:HGNC Symbol;Acc:16736]	-1.28	0.36
ENSG00000179743			-1.28	0.36
ENSG00000260244			-1.28	0.36
ENSG00000141447	OSBPL1A	oxysterol binding protein-like 1A [Source:HGNC Symbol;Acc:16398]	-1.28	0.36
ENSG00000106780	MEGF9	multiple EGF-like-domains 9 [Source:HGNC Symbol;Acc:3234]	-1.28	0.35
ENSG00000167291	TBC1D16	TBC1 domain family, member 16 [Source:HGNC Symbol;Acc:28356]	-1.28	0.35
ENSG00000253250	C8orf88	chromosome 8 open reading frame 88 [Source:HGNC Symbol;Acc:44672]	-1.28	0.35
ENSG00000174059	CD34	CD34 molecule [Source:HGNC Symbol;Acc:1662]	-1.27	0.35
ENSG00000082781	ITGB5	integrin, beta 5 [Source:HGNC Symbol;Acc:6160]	-1.27	0.35
ENSG00000152661	GJA1	gap junction protein, alpha 1, 43kDa [Source:HGNC Symbol;Acc:4274]	-1.27	0.34
ENSG00000165659	DACH1	dachshund homolog 1 (Drosophila) [Source:HGNC Symbol;Acc:2663]	-1.27	0.34
ENSG00000237172	B3GNT9	UDP-GlcNAc:betaGal beta-1,3-N-acetylglucosaminyltransferase 9 [Source:HGNC Symbol;Acc:28714]	-1.27	0.34
ENSG00000019485	PRDM11	PR domain containing 11 [Source:HGNC Symbol;Acc:13996]	-1.27	0.34
ENSG00000150764	DIXDC1	DIX domain containing 1 [Source:HGNC Symbol;Acc:23695]	-1.27	0.34
ENSG00000169255	B3GALNT1	beta-1,3-N-acetylgalactosaminyltransferase 1 (globoside blood group) [Source:HGNC Symbol;Acc:918]	-1.26	0.34
ENSG00000273015	LINC00938	long intergenic non-protein coding RNA 938 [Source:HGNC Symbol;Acc:48630]	-1.26	0.33
ENSG00000154065	ANKRD29	ankyrin repeat domain 29 [Source:HGNC Symbol;Acc:27110]	-1.26	0.33

ENSG00000177888	ZBTB41	zinc finger and BTB domain containing 41 [Source:HGNC Symbol;Acc:24819]	-1.25	0.33
ENSG00000168952	STXBP6	syntaxin binding protein 6 (amisyn) [Source:HGNC Symbol;Acc:19666]	-1.25	0.33
ENSG00000150403	TMCO3	transmembrane and coiled-coil domains 3 [Source:HGNC Symbol;Acc:20329]	-1.25	0.32
ENSG00000198467	TPM2	tropomyosin 2 (beta) [Source:HGNC Symbol;Acc:12011]	-1.25	0.32
ENSG00000072840	EVC	Ellis van Creveld syndrome [Source:HGNC Symbol;Acc:3497]	-1.25	0.32
ENSG00000154380	ENAH	enabled homolog (Drosophila) [Source:HGNC Symbol;Acc:18271]	-1.25	0.32
ENSG00000177868	CCDC23	coiled-coil domain containing 23 [Source:HGNC Symbol;Acc:29204]	-1.25	0.32
ENSG00000267414			-1.25	0.32
ENSG00000153558	FBXL2	F-box and leucine-rich repeat protein 2 [Source:HGNC Symbol;Acc:13598]	-1.25	0.32
ENSG00000156535	CD109	CD109 molecule [Source:HGNC Symbol;Acc:21685]	-1.24	0.31
ENSG00000166173	LARP6	La ribonucleoprotein domain family, member 6 [Source:HGNC Symbol;Acc:24012]	-1.24	0.31
ENSG00000182919	C11orf54	chromosome 11 open reading frame 54 [Source:HGNC Symbol;Acc:30204]	-1.24	0.31
ENSG00000057757	PITHD1	PITH (C-terminal proteasome-interacting domain of thioredoxin-like) domain containing 1 [Source:HGNC Symbol;Acc:25022]	-1.24	0.31
ENSG00000109944	C11orf63	chromosome 11 open reading frame 63 [Source:HGNC Symbol;Acc:26288]	-1.24	0.31
ENSG00000197565	COL4A6	collagen, type IV, alpha 6 [Source:HGNC Symbol;Acc:2208]	-1.24	0.31
ENSG00000109854	HTATIP2	HIV-1 Tat interactive protein 2, 30kDa [Source:HGNC Symbol;Acc:16637]	-1.24	0.31
ENSG00000197093	GAL3ST4	galactose-3-O-sulfotransferase 4 [Source:HGNC Symbol;Acc:24145]	-1.24	0.31
ENSG00000253161			-1.24	0.31
ENSG00000127920	GNG11	guanine nucleotide binding protein (G protein), gamma 11 [Source:HGNC Symbol;Acc:4403]	-1.24	0.31
ENSG00000164574	GALNT10	UDP-N-acetyl-alpha-D-galactosamine:polypeptide N-acetylgalactosaminyltransferase 10 (GalNAc-T10) [Source:HGNC Symbol;Acc:19873]	-1.24	0.31
ENSG00000109686	SH3D19	SH3 domain containing 19 [Source:HGNC Symbol;Acc:30418]	-1.24	0.31
ENSG00000186446	ZNF501	zinc finger protein 501 [Source:HGNC Symbol;Acc:23717]	-1.24	0.31
ENSG00000164615	CAMLG	calcium modulating ligand [Source:HGNC Symbol;Acc:1471]	-1.23	0.3
ENSG00000104490	NCALD	neurocalcin delta [Source:HGNC Symbol;Acc:7655]	-1.23	0.3
ENSG00000197381	ADARB1	adenosine deaminase, RNA-specific, B1 [Source:HGNC Symbol;Acc:226]	-1.23	0.3

ENSG00000118960	HS1BP3	HCLS1 binding protein 3 [Source:HGNC Symbol;Acc:24979]	-1.23	0.3
ENSG00000135299	ANKRD6	ankyrin repeat domain 6 [Source:HGNC Symbol;Acc:17280]	-1.23	0.3
ENSG00000156172	C8orf37	chromosome 8 open reading frame 37 [Source:HGNC Symbol;Acc:27232]	-1.23	0.3
ENSG00000164253	WDR41	WD repeat domain 41 [Source:HGNC Symbol;Acc:25601]	-1.23	0.3
ENSG00000197535	MYO5A	myosin VA (heavy chain 12, myoxin) [Source:HGNC Symbol;Acc:7602]	-1.23	0.3
ENSG00000066629	EML1	echinoderm microtubule associated protein like 1 [Source:HGNC Symbol;Acc:3330]	-1.23	0.29
ENSG00000143772	ITPKB	inositol-trisphosphate 3-kinase B [Source:HGNC Symbol;Acc:6179]	-1.23	0.29
ENSG00000163577	EIF5A2	eukaryotic translation initiation factor 5A2 [Source:HGNC Symbol;Acc:3301]	-1.23	0.29
ENSG00000122378	FAM213A	family with sequence similarity 213, member A [Source:HGNC Symbol;Acc:28651]	-1.22	0.29
ENSG00000138434	SSFA2	sperm specific antigen 2 [Source:HGNC Symbol;Acc:11319]	-1.22	0.29
ENSG00000052795	FNIP2	folliculin interacting protein 2 [Source:HGNC Symbol;Acc:29280]	-1.22	0.29
ENSG00000092964	DPYSL2	dihydropyrimidinase-like 2 [Source:HGNC Symbol;Acc:3014]	-1.22	0.29
ENSG00000164237	CMBL	carboxymethylenebutenolidase homolog (Pseudomonas) [Source:HGNC Symbol;Acc:25090]	-1.22	0.29
ENSG00000100422	CERK	ceramide kinase [Source:HGNC Symbol;Acc:19256]	-1.22	0.29
ENSG00000143878	RHOB	ras homolog family member B [Source:HGNC Symbol;Acc:668]	-1.22	0.29
ENSG00000153885	KCTD15	potassium channel tetramerization domain containing 15 [Source:HGNC Symbol;Acc:23297]	-1.22	0.29
ENSG00000196150	ZNF250	zinc finger protein 250 [Source:HGNC Symbol;Acc:13044]	-1.22	0.29
ENSG00000163069	SGCB	sarcoglycan, beta (43kDa dystrophin-associated glycoprotein) [Source:HGNC Symbol;Acc:10806]	-1.22	0.29
ENSG00000166845	C18orf54	chromosome 18 open reading frame 54 [Source:HGNC Symbol;Acc:13796]	-1.22	0.29
ENSG00000177469	PTRF	polymerase I and transcript release factor [Source:HGNC Symbol;Acc:9688]	-1.22	0.28
ENSG00000236773			-1.22	0.28
ENSG00000081377	CDC14B	cell division cycle 14B [Source:HGNC Symbol;Acc:1719]	-1.22	0.28
ENSG00000106348	IMPDH1	IMP (inosine 5'-monophosphate) dehydrogenase 1 [Source:HGNC Symbol;Acc:6052]	-1.22	0.28
ENSG00000182700	IGIP	IgA-inducing protein [Source:HGNC Symbol;Acc:33847]	-1.22	0.28
ENSG00000105974	CAV1	caveolin 1, caveolae protein, 22kDa [Source:HGNC Symbol;Acc:1527]	-1.21	0.28
ENSG00000119326	CTNNAL1	catenin (cadherin-associated protein), alpha-like 1 [Source:HGNC Symbol;Acc:2512]	-1.21	0.28

ENSG00000134901	KDELC1	KDEL (Lys-Asp-Glu-Leu) containing 1 [Source:HGNC Symbol;Acc:19350]	-1.21	0.28
ENSG00000167637	ZNF283	zinc finger protein 283 [Source:HGNC Symbol;Acc:13077]	-1.21	0.28
ENSG00000014914	MTMR11	myotubularin related protein 11 [Source:HGNC Symbol;Acc:24307]	-1.21	0.28
ENSG00000138386	NAB1	NGFI-A binding protein 1 (EGR1 binding protein 1) [Source:HGNC Symbol;Acc:7626]	-1.21	0.28
ENSG00000184319	RPL23AP82	ribosomal protein L23a pseudogene 82 [Source:HGNC Symbol;Acc:33730]	-1.21	0.27
ENSG00000165572	KBTBD6	kelch repeat and BTB (POZ) domain containing 6 [Source:HGNC Symbol;Acc:25340]	-1.21	0.27
ENSG00000196741	CXorf24	chromosome X open reading frame 24 [Source:HGNC Symbol;Acc:27333]	-1.21	0.27
ENSG00000141542	RAB40B	RAB40B, member RAS oncogene family [Source:HGNC Symbol;Acc:18284]	-1.21	0.27
ENSG00000167202	TBC1D2B	TBC1 domain family, member 2B [Source:HGNC Symbol;Acc:29183]	-1.21	0.27
ENSG00000073417	PDE8A	phosphodiesterase 8A [Source:HGNC Symbol;Acc:8793]	-1.2	0.26
ENSG00000171150	SOCS5	suppressor of cytokine signaling 5 [Source:HGNC Symbol;Acc:16852]	-1.2	0.26
ENSG00000176903	PNMA1	paraneoplastic Ma antigen 1 [Source:HGNC Symbol;Acc:9158]	-1.2	0.26
ENSG00000185088	RPS27L	ribosomal protein S27-like [Source:HGNC Symbol;Acc:18476]	-1.2	0.26
ENSG00000070371	CLTCL1	clathrin, heavy chain-like 1 [Source:HGNC Symbol;Acc:2093]	-1.2	0.26
ENSG00000119280	C1orf198	chromosome 1 open reading frame 198 [Source:HGNC Symbol;Acc:25900]	-1.2	0.26
ENSG00000070214	SLC44A1	solute carrier family 44 (choline transporter), member 1 [Source:HGNC Symbol;Acc:18798]	-1.19	0.25
ENSG00000214756	METTL12	methyltransferase like 12 [Source:HGNC Symbol;Acc:33113]	-1.19	0.25
ENSG00000110931	CAMKK2	calcium/calmodulin-dependent protein kinase kinase 2, beta [Source:HGNC Symbol;Acc:1470]	-1.19	0.25
ENSG00000180304	OAZ2	ornithine decarboxylase antizyme 2 [Source:HGNC Symbol;Acc:8096]	-1.19	0.25
ENSG00000217416	ISCA1P1	iron-sulfur cluster assembly 1 pseudogene 1 [Source:HGNC Symbol;Acc:33263]	-1.19	0.25
ENSG00000255529	POLR2M	polymerase (RNA) II (DNA directed) polypeptide M [Source:HGNC Symbol;Acc:14862]	-1.19	0.25
ENSG00000143198	MGST3	microsomal glutathione S-transferase 3 [Source:HGNC Symbol;Acc:7064]	-1.18	0.24
ENSG00000113595	TRIM23	tripartite motif containing 23 [Source:HGNC Symbol;Acc:660]	-1.18	0.24
ENSG00000111802	TDP2	tyrosyl-DNA phosphodiesterase 2 [Source:HGNC Symbol;Acc:17768]	-1.18	0.24
ENSG00000170275	CRTAP	cartilage associated protein [Source:HGNC Symbol;Acc:2379]	-1.18	0.24
ENSG00000144677	CTDSPL	CTD (carboxy-terminal domain, RNA polymerase II, polypeptide A) small phosphatase-like [Source:HGNC Symbol;Acc:16890]	-1.18	0.24

ENSG00000075945	KIFAP3	kinesin-associated protein 3 [Source:HGNC Symbol;Acc:17060]	-1.18	0.24
ENSG00000120963	ZNF706	zinc finger protein 706 [Source:HGNC Symbol;Acc:24992]	-1.18	0.24
ENSG00000131378	RFTN1	raftlin, lipid raft linker 1 [Source:HGNC Symbol;Acc:30278]	-1.18	0.24
ENSG00000069966	GNB5	guanine nucleotide binding protein (G protein), beta 5 [Source:HGNC Symbol;Acc:4401]	-1.18	0.23
ENSG00000102181	CD99L2	CD99 molecule-like 2 [Source:HGNC Symbol;Acc:18237]	-1.18	0.23
ENSG00000110880	CORO1C	coronin, actin binding protein, 1C [Source:HGNC Symbol;Acc:2254]	-1.17	0.23
ENSG00000163697	APBB2	amyloid beta (A4) precursor protein-binding, family B, member 2 [Source:HGNC Symbol;Acc:582]	-1.17	0.23
ENSG00000247556	OIP5-AS1	OIP5 antisense RNA 1 [Source:HGNC Symbol;Acc:43563]	-1.17	0.23
ENSG00000175970	UNC119B	unc-119 homolog B (C. elegans) [Source:HGNC Symbol;Acc:16488]	-1.17	0.23
ENSG00000100320	RBFOX2	RNA binding protein, fox-1 homolog (C. elegans) 2 [Source:HGNC Symbol;Acc:9906]	-1.17	0.23
ENSG00000119185	ITGB1BP1	integrin beta 1 binding protein 1 [Source:HGNC Symbol;Acc:23927]	-1.17	0.23
ENSG00000128923	FAM63B	family with sequence similarity 63, member B [Source:HGNC Symbol;Acc:26954]	-1.17	0.23
ENSG00000133142	TCEAL4	transcription elongation factor A (SII)-like 4 [Source:HGNC Symbol;Acc:26121]	-1.17	0.22
ENSG00000135070	ISCA1	iron-sulfur cluster assembly 1 [Source:HGNC Symbol;Acc:28660]	-1.17	0.22
ENSG00000151748	SAV1	salvador homolog 1 (Drosophila) [Source:HGNC Symbol;Acc:17795]	-1.17	0.22
ENSG00000154059	IMPACT	impact RWD domain protein [Source:HGNC Symbol;Acc:20387]	-1.17	0.22
ENSG00000124098	FAM210B	family with sequence similarity 210, member B [Source:HGNC Symbol;Acc:16102]	-1.16	0.22
ENSG00000149218	ENDOD1	endonuclease domain containing 1 [Source:HGNC Symbol;Acc:29129]	-1.16	0.22
ENSG00000156384	SFR1	SWI5-dependent recombination repair 1 [Source:HGNC Symbol;Acc:29574]	-1.16	0.21
ENSG00000181904	C5orf24	chromosome 5 open reading frame 24 [Source:HGNC Symbol;Acc:26746]	-1.16	0.21
ENSG00000242259	C22orf39	chromosome 22 open reading frame 39 [Source:HGNC Symbol;Acc:27012]	-1.16	0.21
ENSG00000254244	PAICSP4	phosphoribosylaminoimidazole carboxylase, phosphoribosylaminoimidazole succinocarboxamide synthetase pseudogene 4 [Source:HGNC Symbol;Acc:38097]	-1.16	0.21
ENSG00000113638	TTC33	tetratricopeptide repeat domain 33 [Source:HGNC Symbol;Acc:29959]	-1.16	0.21
ENSG00000131171	SH3BGRL	SH3 domain binding glutamic acid-rich protein like [Source:HGNC Symbol;Acc:10823]	-1.16	0.21
ENSG00000185787	MORF4L1	mortality factor 4 like 1 [Source:HGNC Symbol;Acc:16989]	-1.16	0.21
ENSG00000108100	CCNY	cyclin Y [Source:HGNC Symbol;Acc:23354]	-1.15	0.21

ENSG00000166272	WBP1L	WW domain binding protein 1-like [Source:HGNC Symbol;Acc:23510]	-1.15	0.21
ENSG00000145391	SETD7	SET domain containing (lysine methyltransferase) 7 [Source:HGNC Symbol;Acc:30412]	-1.15	0.2
ENSG00000219201			-1.15	0.2
ENSG00000225733	FGD5-AS1	FGD5 antisense RNA 1 [Source:HGNC Symbol;Acc:40410]	-1.15	0.2
ENSG00000152465	NMT2	N-myristoyltransferase 2 [Source:HGNC Symbol;Acc:7858]	-1.15	0.2
ENSG00000091436		Mitogen-activated protein kinase kinase kinase MLT [Source:UniProtKB/Swiss-Prot;Acc:Q9NYL2]	-1.15	0.2
ENSG00000106723	SPIN1	spindlin 1 [Source:HGNC Symbol;Acc:11243]	-1.14	0.19
ENSG00000113658	SMAD5	SMAD family member 5 [Source:HGNC Symbol;Acc:6771]	-1.14	0.19
ENSG00000019549	SNAI2	snail family zinc finger 2 [Source:HGNC Symbol;Acc:11094]	-1.14	0.19
ENSG00000020256	ZFP64	ZFP64 zinc finger protein [Source:HGNC Symbol;Acc:15940]	-1.14	0.19
ENSG00000154640	BTG3	BTG family, member 3 [Source:HGNC Symbol;Acc:1132]	-1.14	0.19
ENSG00000075239	ACAT1	acetyl-CoA acetyltransferase 1 [Source:HGNC Symbol;Acc:93]	-1.14	0.19
ENSG00000112186	CAP2	CAP, adenylate cyclase-associated protein, 2 (yeast) [Source:HGNC Symbol;Acc:20039]	-1.14	0.19
ENSG00000224531	SMIM13	small integral membrane protein 13 [Source:HGNC Symbol;Acc:27356]	-1.14	0.19
ENSG00000159200	RCAN1	regulator of calcineurin 1 [Source:HGNC Symbol;Acc:3040]	-1.14	0.19
ENSG00000107614	TRDMT1	tRNA aspartic acid methyltransferase 1 [Source:HGNC Symbol;Acc:2977]	-1.14	0.19
ENSG00000163428	LRRC58	leucine rich repeat containing 58 [Source:HGNC Symbol;Acc:26968]	-1.14	0.19
ENSG00000177096	FAM109B	family with sequence similarity 109, member B [Source:HGNC Symbol;Acc:27161]	-1.14	0.19
ENSG00000102038	SMARCA1	SWI/SNF related, matrix associated, actin dependent regulator of chromatin, subfamily a, member 1 [Source:HGNC Symbol;Acc:11097]	-1.14	0.18
ENSG00000213753	CENPBD1P1	CENPBD1 pseudogene 1 [Source:HGNC Symbol;Acc:28421]	-1.14	0.18
ENSG00000114491	UMPS	uridine monophosphate synthetase [Source:HGNC Symbol;Acc:12563]	-1.13	0.18
ENSG00000154124	FAM105B	family with sequence similarity 105, member B [Source:HGNC Symbol;Acc:25118]	-1.13	0.18
ENSG00000128989	ARPP19	cAMP-regulated phosphoprotein, 19kDa [Source:HGNC Symbol;Acc:16967]	-1.13	0.18
ENSG00000223768	LINC00205	long intergenic non-protein coding RNA 205 [Source:HGNC Symbol;Acc:16420]	-1.13	0.18
ENSG00000101190	TCFL5	transcription factor-like 5 (basic helix-loop-helix) [Source:HGNC Symbol;Acc:11646]	-1.13	0.18
ENSG00000164983	TMEM65	transmembrane protein 65 [Source:HGNC Symbol;Acc:25203]	-1.13	0.18

ENSG00000165046	LETM2	leucine zipper-EF-hand containing transmembrane protein 2 [Source:HGNC Symbol;Acc:14648]	-1.13	0.18
ENSG00000133059	DSTYK	dual serine/threonine and tyrosine protein kinase [Source:HGNC Symbol;Acc:29043]	-1.13	0.17
ENSG00000186063	AIDA	axin interactor, dorsalization associated [Source:HGNC Symbol;Acc:25761]	-1.13	0.17
ENSG00000198612	COPS8	COP9 signalosome subunit 8 [Source:HGNC Symbol;Acc:24335]	-1.13	0.17
ENSG00000128791	TWSG1	twisted gastrulation BMP signaling modulator 1 [Source:HGNC Symbol;Acc:12429]	-1.13	0.17
ENSG00000101752	MIB1	mindbomb E3 ubiquitin protein ligase 1 [Source:HGNC Symbol;Acc:21086]	-1.12	0.17
ENSG00000124641	MED20	mediator complex subunit 20 [Source:HGNC Symbol;Acc:16840]	-1.12	0.17
ENSG00000157240	FZD1	frizzled family receptor 1 [Source:HGNC Symbol;Acc:4038]	-1.12	0.17
ENSG00000160199	PKNOX1	PBX/knotted 1 homeobox 1 [Source:HGNC Symbol;Acc:9022]	-1.12	0.17
ENSG00000169756	LIMS1	LIM and senescent cell antigen-like domains 1 [Source:HGNC Symbol;Acc:6616]	-1.12	0.17
ENSG00000172667	ZMAT3	zinc finger, matrin-type 3 [Source:HGNC Symbol;Acc:29983]	-1.12	0.17
ENSG00000048471	SNX29	sorting nexin 29 [Source:HGNC Symbol;Acc:30542]	-1.12	0.17
ENSG00000109184	DCUN1D4	DCN1, defective in cullin neddylation 1, domain containing 4 [Source:HGNC Symbol;Acc:28998]	-1.12	0.16
ENSG00000205133	TRIQK	triple QxxK/R motif containing [Source:HGNC Symbol;Acc:27828]	-1.12	0.16
ENSG00000119729	RHOQ	ras homolog family member Q [Source:HGNC Symbol;Acc:17736]	-1.12	0.16
ENSG00000151233	GXYLT1	glucoside xylosyltransferase 1 [Source:HGNC Symbol;Acc:27482]	-1.12	0.16
ENSG00000162520	SYNC	syncoilin, intermediate filament protein [Source:HGNC Symbol;Acc:28897]	-1.12	0.16
ENSG00000185420	SMYD3	SET and MYND domain containing 3 [Source:HGNC Symbol;Acc:15513]	-1.12	0.16
ENSG00000102931	ARL2BP	ADP-ribosylation factor-like 2 binding protein [Source:HGNC Symbol;Acc:17146]	-1.12	0.16
ENSG00000121644	DESI2	desumoylating isopeptidase 2 [Source:HGNC Symbol;Acc:24264]	-1.12	0.16
ENSG00000171862	PTEN	phosphatase and tensin homolog [Source:HGNC Symbol;Acc:9588]	-1.12	0.16
ENSG00000141720	PIP4K2B	phosphatidylinositol-5-phosphate 4-kinase, type II, beta [Source:HGNC Symbol;Acc:8998]	-1.11	0.16
ENSG00000237493			-1.11	0.16
ENSG00000237984	PTENP1	phosphatase and tensin homolog pseudogene 1 [Source:HGNC Symbol;Acc:9589]	-1.11	0.16
ENSG00000106993	CDC37L1	cell division cycle 37-like 1 [Source:HGNC Symbol;Acc:17179]	-1.11	0.15
ENSG00000069275	NUCKS1	nuclear casein kinase and cyclin-dependent kinase substrate 1 [Source:HGNC Symbol;Acc:29923]	-1.11	0.15

ENSG00000218283	MORF4L1P1	mortality factor 4 like 1 pseudogene 1 [Source:HGNC Symbol;Acc:20400]	-1.11	0.15
ENSG00000168246	UBTD2	ubiquitin domain containing 2 [Source:HGNC Symbol;Acc:24463]	-1.11	0.15
ENSG00000168214	RBPJ	recombination signal binding protein for immunoglobulin kappa J region [Source:HGNC Symbol;Acc:5724]	-1.11	0.15
ENSG00000196715	VKORC1L1	vitamin K epoxide reductase complex, subunit 1-like 1 [Source:HGNC Symbol;Acc:21492]	-1.1	0.14
ENSG00000087053	MTMR2	myotubularin related protein 2 [Source:HGNC Symbol;Acc:7450]	-1.1	0.14
ENSG00000155096	AZIN1	antizyme inhibitor 1 [Source:HGNC Symbol;Acc:16432]	-1.1	0.14
ENSG00000188636	LDOC1L	leucine zipper, down-regulated in cancer 1-like [Source:HGNC Symbol;Acc:13343]	-1.1	0.14
ENSG00000079950	STX7	syntaxin 7 [Source:HGNC Symbol;Acc:11442]	-1.1	0.14
ENSG00000108861	DUSP3	dual specificity phosphatase 3 [Source:HGNC Symbol;Acc:3069]	-1.1	0.14
ENSG00000174695	TMEM167A	transmembrane protein 167A [Source:HGNC Symbol;Acc:28330]	-1.1	0.14
ENSG00000119402	FBXW2	F-box and WD repeat domain containing 2 [Source:HGNC Symbol;Acc:13608]	-1.1	0.13
ENSG00000138279	ANXA7	annexin A7 [Source:HGNC Symbol;Acc:545]	-1.1	0.13
ENSG00000100811	YY1	YY1 transcription factor [Source:HGNC Symbol;Acc:12856]	-1.09	0.13
ENSG00000168036	CTNNB1	catenin (cadherin-associated protein), beta 1, 88kDa [Source:HGNC Symbol;Acc:2514]	-1.09	0.13
ENSG00000141646	SMAD4	SMAD family member 4 [Source:HGNC Symbol;Acc:6770]	-1.09	0.12
ENSG00000116747	TROVE2	TROVE domain family, member 2 [Source:HGNC Symbol;Acc:11313]	-1.09	0.12
ENSG00000134759	ELP2	elongator acetyltransferase complex subunit 2 [Source:HGNC Symbol;Acc:18248]	-1.09	0.12
ENSG00000224546	EIF4BP3	eukaryotic translation initiation factor 4B pseudogene 3 [Source:HGNC Symbol;Acc:37936]	-1.08	0.11
ENSG00000120162	MOB3B	MOB kinase activator 3B [Source:HGNC Symbol;Acc:23825]	-1.08	0.11
ENSG00000206562	METTL6	methyltransferase like 6 [Source:HGNC Symbol;Acc:28343]	-1.08	0.11
ENSG00000132405	TBC1D14	TBC1 domain family, member 14 [Source:HGNC Symbol;Acc:29246]	-1.08	0.11
ENSG00000135482	ZC3H10	zinc finger CCCH-type containing 10 [Source:HGNC Symbol;Acc:25893]	-1.08	0.11
ENSG00000078369	GNB1	guanine nucleotide binding protein (G protein), beta polypeptide 1 [Source:HGNC Symbol;Acc:4396]	-1.08	0.11
ENSG00000122550	KLHL7	kelch-like family member 7 [Source:HGNC Symbol;Acc:15646]	-1.07	0.1
ENSG00000135686	KLHL36	kelch-like family member 36 [Source:HGNC Symbol;Acc:17844]	-1.07	0.1
ENSG00000086475	SEPHS1	selenophosphate synthetase 1 [Source:HGNC Symbol;Acc:19685]	-1.07	0.1

ENSG00000205531	NAP1L4	nucleosome assembly protein 1-like 4 [Source:HGNC Symbol;Acc:7640]	-1.07	0.09
ENSG00000161217	PCYT1A	phosphate cytidylyltransferase 1, choline, alpha [Source:HGNC Symbol;Acc:8754]	-1.07	0.09
ENSG00000184743	ATL3	atlastin GTPase 3 [Source:HGNC Symbol;Acc:24526]	-1.05	0.08

Table S3 Protein expression of MUC4 and MMP7 in gingival biopsies from patients with periodontitis and control subjects.

Periodontitis				Healthy subjects			
	MUC4	MMP7			MUC4	MMP7	
Subject	Epithelium[a]	Epithelium (0-3)[b]	Connective tissue (0-3)[b]	Subject	Epithelium[a]	Epithelium (0-3)[b]	Connective tissue (0-3)[b]
**1**	-	2	2	**21**	-	1	1
**2**	+	0	1	**22**	-	2	2
**3**	+	1	1	**23**	-	1	1
**4**	+	1	1	**24**	-	3	2
**5**	+	1	1	**25**	-	1	1
**6**	+	2	3	**26**	-	0	0
**7**	+	3	3	**27**	+	0	0
**8**	+	0	0	**28**	+	3	3
**9**	+	1	1	**29**	-	1	1
**10**	-	0	0	**30**	-	0	0
**11**	+	1	1	**31**	-	2	0
**12**	+	3	3	**32**	-	0	0
**13**	+	2	2	**33**	-	2	2
**14**	+	2	3	**34**	-	0	0
**15**	+	2	2	**35**	-	1	1
**16**	+	3	3	**36**	-	1	1
**17**	+	3	3	**37**	-	1	0
**18**	+	3	3	**38**	-	3	3
**19**	+	2	2	**39**	-	2	2
**20**	+	3	3	**40**	-	2	2
	**Frequency (+/-)**	**Mean value**			**Frequency (+/-)**	**Mean value**	
	18/2[c]	1.6[d]	2.1[e]		2/18[c]	1.3[d]	1.0[e]

[a](+) evidence of MUC4 expression, (-) no evidence of MUC4 expression.
[b]0 = no evidence of inflammatory infiltration, 1 = slight inflammatory infiltration, 2 = moderate inflammatory infiltration and 3 = severe inflammatory infiltration.
[c]$P < 0.0001$ for the difference between periodontitis and controls.
[d]$P = 0.40$ for the difference between periodontitis and controls.
[e]$P < 0.01$ for the difference between periodontitis and controls.
